# ChemoID-guided therapy improves objective response rate in recurrent platinum-resistant ovarian cancer randomized clinical trial

**DOI:** 10.1038/s41698-025-00874-0

**Published:** 2025-03-25

**Authors:** Thomas J. Herzog, Thomas C. Krivak, Stephen Bush, John P. Diaz, Scott Lentz, Navya Nair, Nadim Bou Zgheib, Camille Gunderson-Jackson, Abhijit Barve, Krista L. Denning, Seth T. Lirette, Candace M. Howard, Jagan Valluri, Pier Paolo Claudio

**Affiliations:** 1 Department of Obstetrics and Gynecology, University of Cincinnati Cancer Center, Cincinnati, USA; 2 Division of Gynecologic Oncology, Allegheny Health Network Cancer Institute, Pittsburgh, USA; 3 Department of Obstetrics and Gynecology, Charleston Area Medical Center Vandalia Health, Charleston, USA; 4 Gynecologic Oncology, Baptist Health South Florida, Miami Cancer Institute, Miami, USA; 5 Gynecology Oncology Department, Kaiser Permanente Los Angeles Medical Center, Los Angeles, USA; 6 Department of Obstetrics and Gynecology, Section of Gynecologic Oncology, Louisiana State University, New Orleans, USA; 7 Currently, Division of Gynecologic Oncology, Department of Obstetrics and Gynecology, University of Miami Sylvester Cancer Center, Miami, USA; 8 Edwards Comprehensive Cancer Center, Joan C. Edwards School of Medicine, Marshall University, Huntington, USA; 9 Department of Obstetrics and Gynecology, Section of Gynecologic Oncology, Stephenson Cancer Center, University of Oklahoma Health Sciences Center, Oklahoma City, USA; 10 Currently, Mercy Clinic Gynecologic Oncology, Oklahoma City, USA; 11 Clinical Development & Medical Affairs, Viatris Inc, Canonsburg, USA; 12 Department of Pathology, Joan C. Edwards School of Medicine, Marshall University, Huntington, USA; 13 Department of Data Science, University of Mississippi Medical Center, Translational Research Center, Jackson, USA; 14 Department of Radiology, University of Mississippi Medical Center, Jackson, USA; 15 Cordgenics, LLC, Huntington, USA; 16 Department of Biological Sciences, Marshall University, Huntington, USA; 17 Department of Pharmacology & Toxicology, University of Mississippi Medical Center, Jackson, USA

**Keywords:** Ovarian cancer, Ovarian cancer, Translational research

## Abstract

Patients with recurrent platinum-resistant ovarian cancer (**PROC**) have poor clinical outcomes, owing mainly to the presence of therapy-resistant cancer stem cells (**CSCs**). The **NCT03949283** randomized clinical trial enrolled patients with recurrent PROC to receive ChemoID-guided chemotherapy or the best physician-choice regimen selected from the same list of thirteen mono or combination chemotherapies. The primary outcome was objective response rate (**ORR**) assessed on CT scans using the RECIST 1.1 criteria at 6 months follow-up. Subjects treated with the ChemoID assay had an ORR of 55% (CI_95_ 39% - 73%), compared to 5% (CI_95_ 0% - 11%) for those treated with physician’s choice chemotherapy (*p* <0.0001). Secondary endpoints of duration of response (**DOR**) and progression-free survival (**PFS**) of subjects treated with chemotherapies guided by the ChemoID assay versus physician’s choice chemotherapy were a median of 8 months vs. 5.5 months (*p* <0.0001), and 11.0 months (CI_95_ 8.0– NA) vs 3.0 months (CI_95_ 2.0– 3.5) with 27% of hazard ratio (CI95, 0.15–0.49; *p* <0.001), respectively.

## Introduction

Recurrent epithelial ovarian cancer (EOC) is associated with therapy resistance, with significant mortality and a median survival of only 12–24 months^1^. This is in part attributed to the presence of ovarian cancer stem cells (O-CSCs)^2^. O-CSCs are primarily responsible not only for the growth of ovarian cancer and peritoneal spread but also for the development of chemoresistance and tumor recurrence, thus having profound implications for the treatment of this deadly disease^3^. O-CSCs account for a small subpopulation in the primary tumor, which is enriched in recurrent disease mostly due to the selection of drug-resistant CSCs post-chemotherapy treatment.

Although platinum-based regimens are initially effective in a high percentage of EOC cases, unfortunately, most patients at relapse develop platinum-resistant disease. In relapsed EOC, the guideline to use platinum-based chemotherapy has developed into a somewhat arbitrary time-based approach based on observations that are almost 30 years old^4^. According to this guideline, patients with tumors considered ‘platinum-resistant’ and thus predicted not to respond to platinum-based treatments if the interval is less than 6 months have a limited number of drugs available for treatment. The use of a binary calendar-based cut-off has been recently critiqued, as it has been observed that tumor response to platinum-based chemotherapy increases gradually with treatment-free intervals for platinum-based chemotherapy (TFIp) in a non-categorical fashion^5,6^. Recent evidence has shown that patients with a TFIp of less than 6 months still have a reasonable chance to respond to further platinum-based chemotherapy^7–13^, demonstrating high response, disease control rates, and long-term OS following platinum rechallenge therapy^6–19^.

Objective response rate (ORR) and duration of response (DOR) to chemotherapy for patients with recurrent platinum-resistant disease are significantly lower than those observed in patients with platinum-sensitive disease. In platinum-resistant disease patients treated with single agents, ORRs range from 5%-30%, and the duration of response is typically less than 6 months to chemotherapeutic agents such as pegylated liposomal doxorubicin (PLD), topotecan, taxanes, etoposide, and gemcitabine^20^. However, none of these studies have addressed or explored the idea of reducing the burden of CSCs in PROC to enable a greater and more durable response to therapy. Despite results demonstrating treatment advances, regimens for recurrent PROC are unfortunately not curative and there is an urgent need to develop alternative therapeutic strategies.

Therapeutic regimens to treat recurrent PROC are ordinarily decided upon analysis of individual responses to prior therapies, and the selection of the drugs is usually based on previous drugs administered, previous toxicities experienced by the patient, comorbidities, toxicity profile, and patient preference. Over 20 different regimens are recommended for treatment in patients experiencing recurrence less than 6 months from previous platinum treatment (platinum-resistant recurrent disease)^21–24^, with limited guidance on how to select among the numerous treatment options. Thus, in the absence of specific directives beyond the primary setting, treatment choices for recurrent PROC patients are made primarily empirically^25^.

A strategy to increase survival in cancer patients is to target the cancer stem cells (CSCs) that contribute to therapy resistance and cancer progression^26,27^ by utilizing the cytotoxic chemotherapies routinely covered by Medicare and health insurance plans^28–31^. While there are newer targeted therapies available for ovarian cancer, this trial focused on screening standard-of-care chemotherapies (including platinum-based regimens) that are routinely covered and widely available to community oncology patients globally, where novel agents are not as readily available.

We have developed a chemotherapeutic assay (ChemoID) that is CLIA (Clinical Laboratory Improvement Amendments) and CAP (College of American Pathologists) certified and performed by an independent Hospital Pathology laboratory to help physicians select appropriate chemotherapies for individual patients based on the cytotoxicity profile of CSCs and the bulk of tumor cells response to chemotherapies. The ChemoID assay is a functional precision medicine assay and its goal is twofold: (1) To find the most effective chemotherapies that kill the bulk of tumor cells, causing a reduction in tumor size. (2) To find the most effective drugs that decrease the CSCs load, thereby limiting recurrent disease potential and improving patient outcomes.

The ChemoID assay starts by taking a fresh sterile biopsy from a patient’s tumor which is used to generate a primary cancer cell line from which CSCs are rapidly expanded in a bioreactor and an ex vivo chemosensitivity assay is used to quantitate the percentage of cell kill that is reported as a continuous number from <10% to 100% cell-kill.

The ChemoID assay measures the cytotoxic effect of clinical doses of standard-of-care chemotherapies on CSCs and the bulk of tumor cells with a prioritized list of effective and ineffective chemotherapies. The goal of the assay is to find the most efficacious agents that would reduce the CSC burden in ovarian cancer, thereby limiting metastatic and recurrent disease potential to help improve patients’ outcomes. Real-world clinical studies demonstrated improved PFS and OS of recurrent EOC patients after treatment with cancer stem cell assay-guided chemotherapy regimens (ChemoID) compared to historical data^30,31^. Based on this real-world data, the multi-institutional randomized clinical trial of patients with recurrent PROC was designed to assess the efficacy of chemotherapy regimens selected by the ChemoID assay compared to standard of care (best physician choice).

## Results

### Study design and patients’ characteristics

The study protocol was approved by the Western Institutional Review Board (WIRB) and each of the participating institutions’ independent ethics committees. Written informed consent was obtained from all participants before study enrollment. Female patients with imaging findings concerning for recurrent lesion of ovarian cancer were screened for the clinical trial. The ChemoID study was designed as a randomized clinical trial to assess whether ChemoID assay-guided selection of chemotherapy improved the objective response rate of recurrent platinum-resistant epithelial ovarian cancer patients compared to best physician-choice chemotherapy regimen selection. Patients were blinded to randomization group assignment. Physician investigators were not provided with ChemoID test results for patients randomized to the physician-choice control arm.

**Fig. 1:**
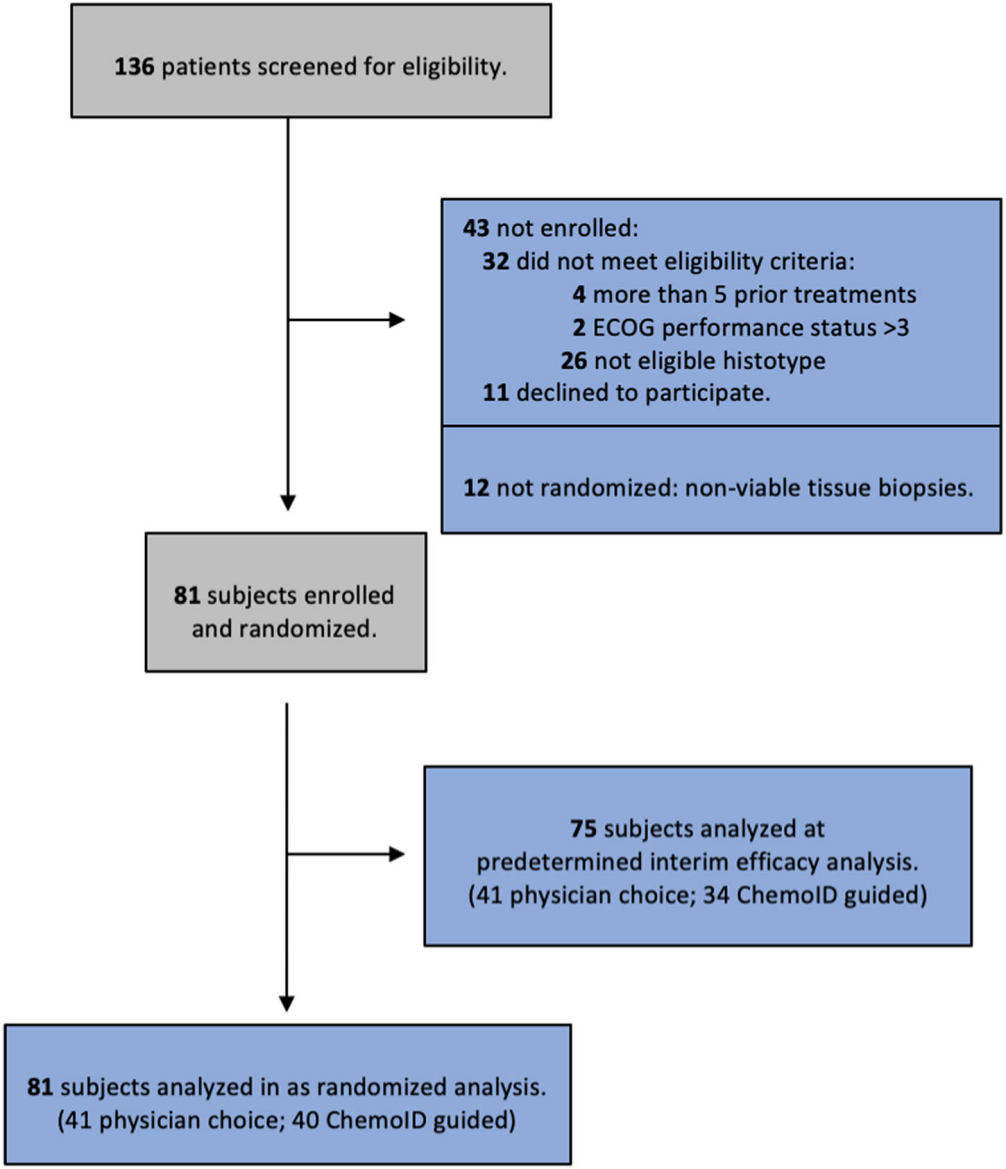
CONSORT diagram of ChemoID study. A total of 136 patients were screened between January 31, 2020, and April 15, 2023; Eighty-one of these patients were randomized to either the ChemoID or physician-choice group. The first prespecified interim analysis was performed when 75 subjects were enrolled in the trial.

**Fig. 2:**
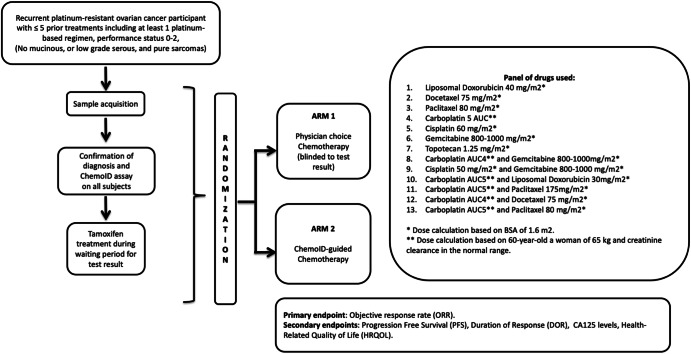
A multi-institutional, randomized clinical trial of patients with recurrent platinum-resistant epithelial ovarian cancer was initiated to assess the efficacy of chemotherapy regimens selected by the ChemoID assay vs. best physician choice. Chemotherapy drugs and doses used in the trial are indicated. The primary endpoint of this trial was objective response rate (ORR). Secondary endpoints were Progression-Free Survival (PFS), duration of response (DOR), measurement of CA125 serum levels, and health-related quality of life.

The IRB protocol was approved on April 11, 2019, and the trial was registered with ClinicalTrials.gov (Identifier NCT03949283) on May 11, 2019. The clinical trial protocol is available in Supplement 1. From January 31, 2020, to April 15, 2023, 136 patients with recurrent PROC were screened and 81 subjects meeting the inclusion and exclusion criteria for study participation (Table 1) were enrolled and randomly assigned 1:1 to either a Physician-choice or a ChemoID-guided treatment group (Consort Diagram - Fig. 1 and RCT schema - Fig. 2).

**Table 1:** Clinical trial inclusion and exclusion criteria

Inclusion Criteria	Exclusion Criteria
1. Informed consent obtained and signed. 2. Participant is willing and able to commit to study procedures including long-term follow-up visit(s); 3. Participant must be a female and at least 18 years of age at the time of enrollment. 4. Negative pregnancy test for women of childbearing potential. 5. Participant has been diagnosed with recurrent platinum resistant epithelial ovarian, peritoneal, or fallopian tube carcinoma. 6. Participant must have measurable disease by imaging or objective physical parameter. 7. Participant has agreed to provide a core biopsy of the primary site, a secondary metastatic site, or a paracentesis or thoracentesis for fluid collection. 8. An adequate fresh sample can be provided to be submitted for ChemoID testing. 9. Participant has disease of one of the following histologic epithelial cell types: high-grade serous adenocarcinoma, endometrioid adenocarcinoma, undifferentiated carcinoma, transitional cell carcinoma, clear cell carcinoma, or adenocarcinoma, not otherwise specified (N.O.S.). Cytologic confirmation of diagnosis is acceptable for participants treated with neoadjuvant therapy who have not had a surgical procedure for a histologic confirmation. Patients with low-grade serous or mucinous adenocarcinoma are not eligible, nor are patients with pure ovarian sarcomas. 10. Participant has received ≤ 5 prior regimens including at least 1 platinum-based regimen for their ovarian, peritoneal, or fallopian tube carcinoma. 11. Participant must have an estimated life expectancy of greater than six months, as determined by the investigator. 12. Participant requires chemotherapy and the investigator plans to administer one of the regimens of interest as deemed by her physician. 13. Participant must have an ECOG Performance Status Score of ≤ 2, KPS ≥ 70, or 0-1 GOG status 14. Adequate laboratory values within 60 days of enrollment to study defined as follows: a. ANC ≥ 1500/mm^3^ b. Hgb ≥ 10 mg/dl c. Hct ≥ 28% d. Platelet count ≥ 100,000/µL e. Serum creatinine ≤ 2.0 mg/dl f. Total bilirubin ≤ 2.5 mg/dl g. AST/SGOT ≤ 3 times ULN. If intrahepatic liver metastases are present, AST and ALT must be ≤ 5 times institutional ULN.	1. Use of Avastin planned to treat participant. 2. Participant has ovarian stromal, germ cell tumors or pure sarcomas. 3. Participant has borderline carcinoma (uncertain malignant potential) mucinous or low-grade serous carcinoma. 4. Participant is pregnant or lactating. 5. Participants of childbearing potential not employing adequate contraception. 6. Participants who are at risk of failure of compliance to the visit schedules and procedures including those with psychiatric disease that would substantially impact compliance 7. Estimated life expectancy of <6 months, as estimated by the investigator in consultation with participating oncologists. 8. Participants with symptomatic cardiac conditions (i.e. NYHA class III/IV or uncompensated angina). 9. Enrollment in another clinical study that precludes allowing the oncologist to select chemotherapy regimens. 10. Previously participated in this study. 11. Any condition that would, in the opinion of the investigator, place the participant at an unacceptable risk, or render the participant unable to meet the requirements of the protocol (including long-term study follow-up). 12. CA-125 only disease without RECIST 1.1 measurable or otherwise evaluable disease. 13. Participant may not use any complementary or alternative medicines including natural herbal products or folk remedies as they may interfere with the effectiveness of the study treatments.

Although all the histotypes listed in the inclusion criteria were considered (high-grade serous adenocarcinoma, endometrioid adenocarcinoma, undifferentiated carcinoma, transitional cell carcinoma, clear cell carcinoma, or adenocarcinoma not otherwise specified), the trial enrolled only participants affected by recurrent high-grade serous carcinoma.

Demographics and baseline clinical characteristics are summarized in Table 2. The median age was 54.6 for the physician-choice group and 55.3 for the ChemoID-guided group. Treatment-free intervals for platinum-based chemotherapy (TFIp) from the last platinum treatment to the documented disease progression of subjects before trial enrollment were balanced between treatment arms (Table 2).

**Table 2:** Patient demographics and baseline characteristics

	Physician Choice (*N* = 41)	ChemoID Guided (*N* = 40)	*p*-value
Age	54.6 (10.8)	55.3 (11.9)	0.709
Race			
Asian	28 (68%)	30 (75%)	0.897
Black	1 (2%)	0 (0%)	
Hispanic	3 (7%)	2 (5%)	
White	9 (22%)	8 (20%)	
Baseline CA125	1140 (1740)	1308 (1294)	0.125
# of Prior Platinum Treatments			
1	22 (54%)	20 (50%)	0.599
2	13 (32%)	13 (33%)	
3	2 (5%)	5 (12%)	
4	4 (8%)	2 (5%)	
ECOG			
0	5 (12%)	4 (10%)	>0.999
1	35 (85%)	36 (90%)	
2	1 (2%)	0 (0%)	
Median TFIp (months)	21.0	20.0	

Three prespecified interim analyses were planned a priori at 75, 100, and 150 at a total sample size of 220. The first interim data analysis was performed at the end of December 2023 when 75 patients had completed the required follow-ups in the trial. Trial enrollment was stopped because of having satisfied the primary endpoint for efficacy after the first interim data analysis, and a total of 81 subjects were analyzed at the final analysis.

### Primary trial endpoint analysis: ChemoID assay-guided therapy improves the ORR of recurrent platinum-resistant EOC patients

**Fig. 3:**
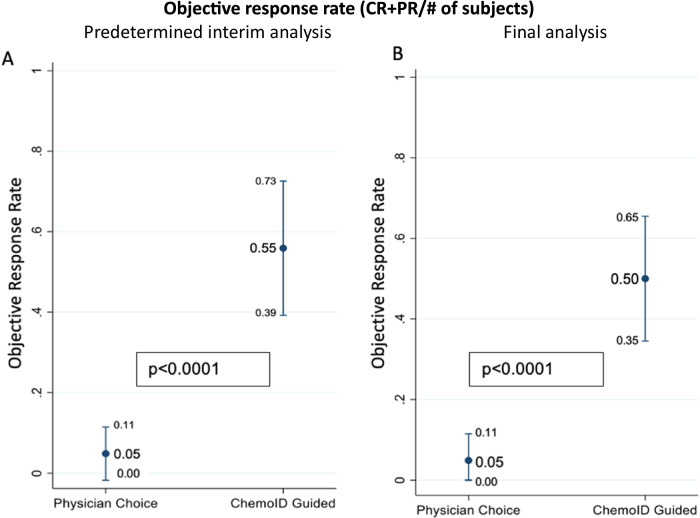
ORR is significantly improved by ChemoID-guided therapy. **A** Prespecified interim analysis of ORR. **B** Final Analysis of ORR.

At the first predetermined interim analysis, the primary outcome of ORR, defined as the proportion of patients with a complete response (CR) or partial response (PR) to treatment according to Response Evaluation Criteria in Solid Tumors - RECIST 1.1, measured at 6 months follow-up visit showed an odds ratio (OR) comparing odds of objective response between the physician choice group (numerator) and the ChemoID Assay group (denominator) of 0.044 with a *p*-value <0.0001. The ORR of subjects treated with chemotherapies guided by the ChemoID assay was 55% (CI_95_ 39–73%) and the ORR of subjects treated with chemotherapies empirically chosen by the physicians was 5% (CI_95_ 0–11%) with a *p*-value <0.0001 (Fig. 3A). ChemoID assay-guided therapy continued to demonstrate meaningful benefit in ORR throughout follow-up. In the final analysis, the ORR of subjects treated with chemotherapies guided by the ChemoID assay was 50% (CI_95_ 35–65%) and the ORR of subjects treated with chemotherapies empirically chosen by the physicians was 5% (CI_95_ 0–11%) with a *p*-value <0.0001 (Fig. 3B).

### Secondary trial endpoint analyses: ChemoID assay-guided therapy improves the PFS, DOD, and CBR, and decreases the CA125 levels of recurrent platinum-resistant EOC patients

**Fig. 4:**
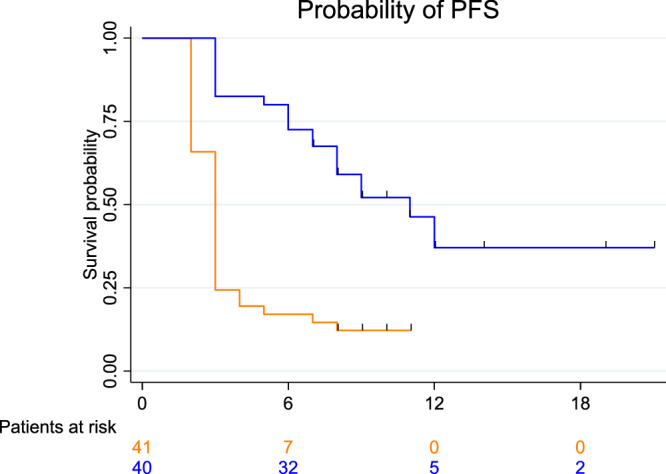
PFS is significantly improved by ChemoID-guided therapy. Final Analysis of PFS. The number of events; median PFS; PFS rates at 0, 6, 12, and 18 months; and the Kaplan–Meier curve for PFS per investigator assessment in patients treated with ChemoID-guided (blue) vs. physician-choice (red) therapies. Symbols indicate censored observations. A Cox proportional hazards model estimated hazard ratios (HRs) and CIs.

In the final analysis, the median progression-free survival (mPFS) was 11.0 months (CI_95_ 8.0–NA) for patients receiving ChemoID assay-guided therapy vs 3.0 months (CI_95_ 2.0–3.5) for physician-choice therapy (HR, 0.27; CI_95_, 0.15–0.49; *p* <0.001) with a log-rank *p* <0.001 (Fig. 4).

**Fig. 5:**
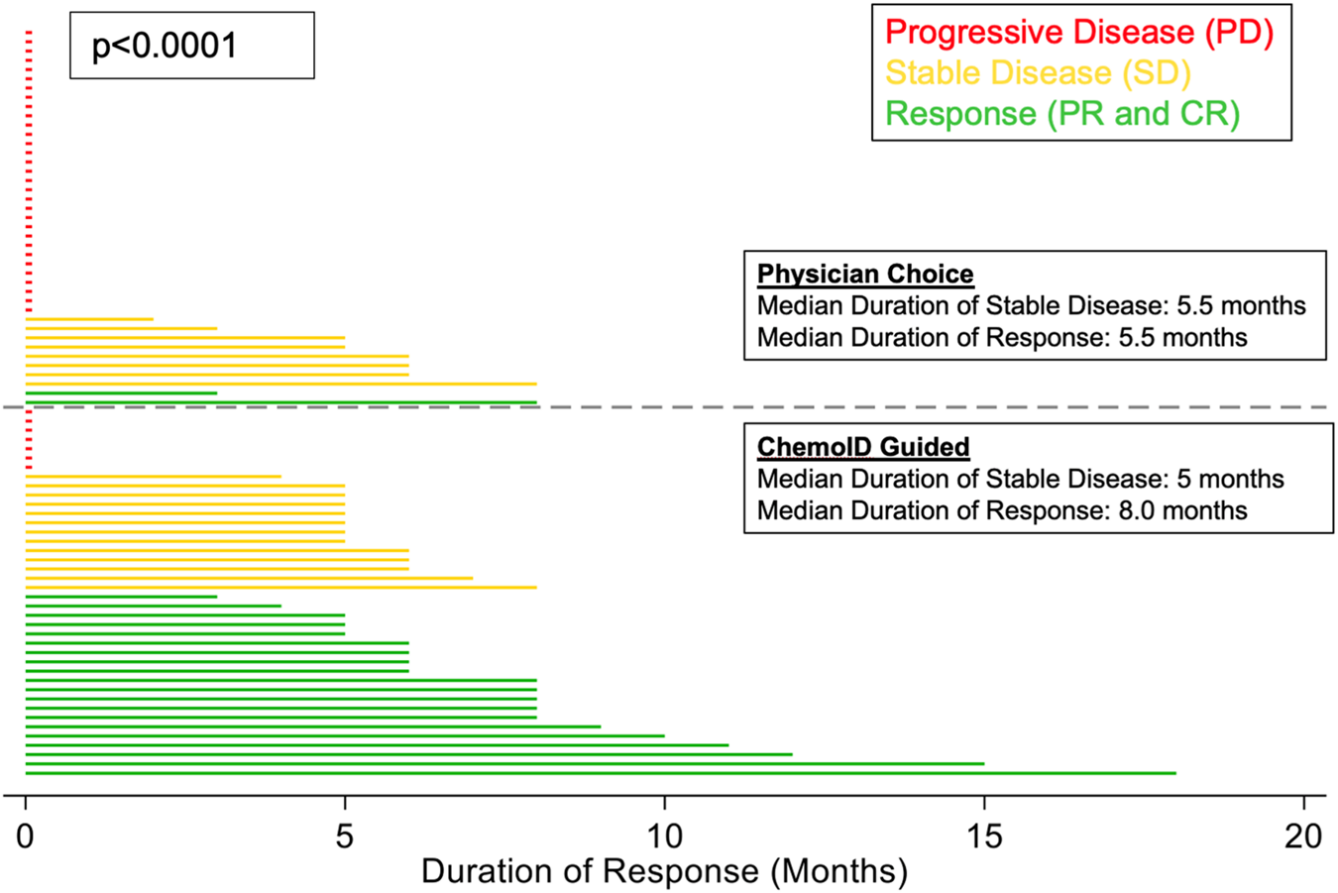
DOR (PR + CR) is significantly improved by ChemoID-guided therapy. Pyramid graph of the duration of response in the two groups. PD is indicated by red bars. SD is indicated by yellow bars. PR and CR are indicated by green bars.

The analysis of duration of response (DOR) showed statistically significant differences when comparing the two groups (*p* <0.0001). Subjects treated with chemotherapies guided by the ChemoID assay showed a mDOR of 8 months and subjects treated with chemotherapies empirically chosen by the physicians had a mDOR of 5.5 months (Fig. 5).

**Fig. 6:**
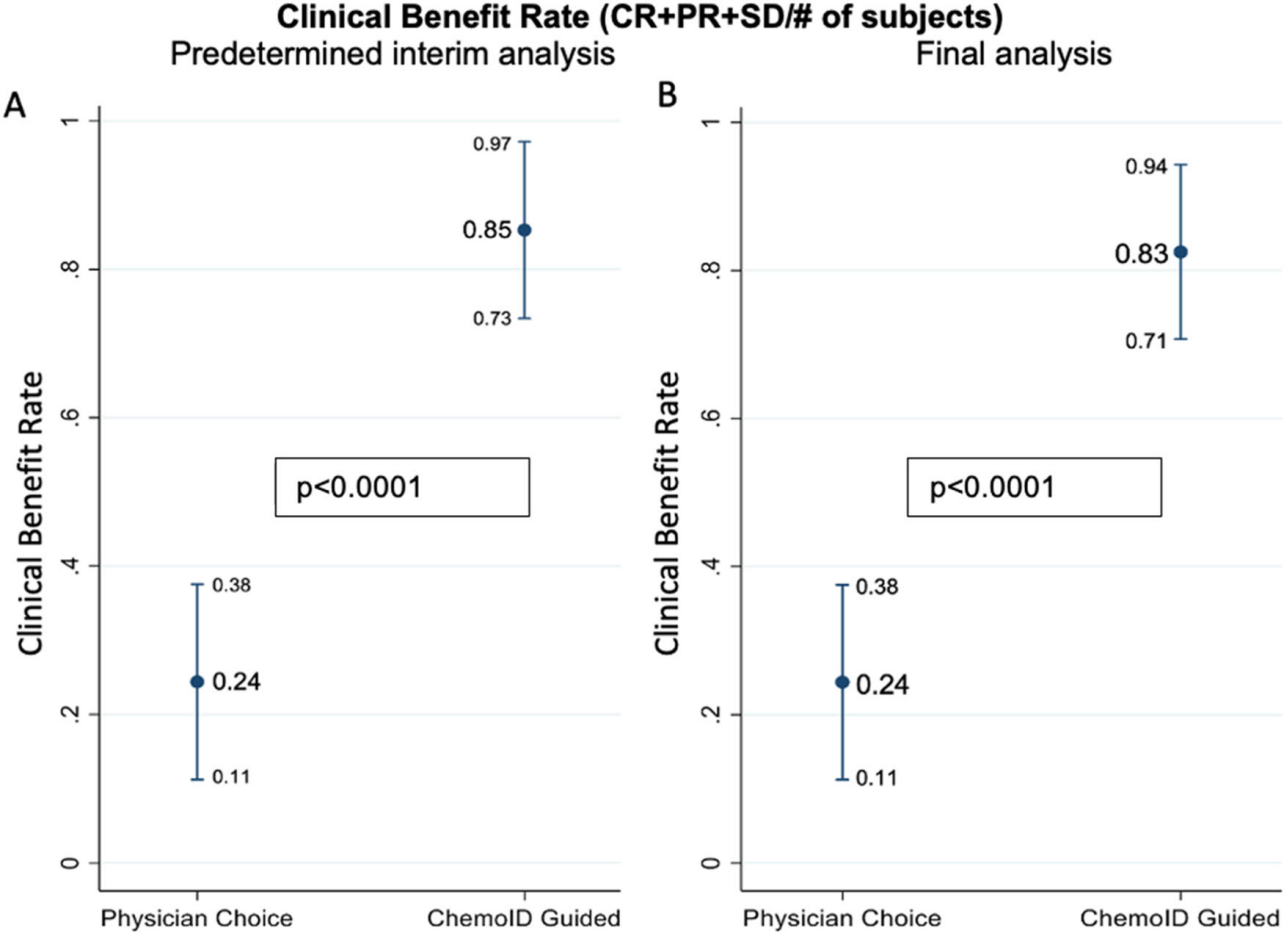
CBR (defined as CR + PR + SD/# of subjects) is significantly improved by ChemoID-guided therapy. **A** Prespecified interim analysis of CBR. **B** Final Analysis of CBR.

In the first interim analysis, the clinical benefit rate (**CBR**) (defined as the proportion of patients with a CR, PR, or stable disease (SD) to treatment according to RECIST 1.1 of subjects treated with chemotherapies guided by the ChemoID assay was 85% (CI_95_ 73–97%) and the CBR of subjects treated with chemotherapies empirically chosen by the physicians was 24% (CI_95_ 11–38%) with a *p*-value <0.0001 (Fig. 6A).

ChemoID assay-guided therapy continued to demonstrate meaningful benefit in CBR throughout follow-up. The CBR of subjects treated with chemotherapies guided by the ChemoID assay was 83% (CI_95_ 71–94%) whereas the CBR of subjects treated with chemotherapies empirically chosen by the physicians was 24% (CI_95_ 11– 38%) with a *p*-value <0.0001 (Fig. 6B).

**Fig. 7:**
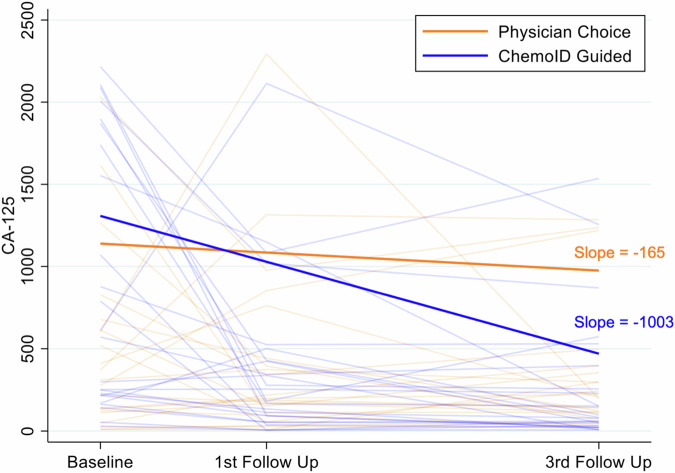
Levels of serum CA125 between screening and third follow-up are improved by ChemoID-guided therapy. Levels of CA125 of patients in the ChemoID-guided group treated with assay-predicted drugs (blue) vs. physician-choice (red) therapies are represented. The slope of the group of patients treated with ChemoID-guided (blue) vs. physician-choice (red) is represented.

The association between treatment groups and tumor response to treatment was not modified by the levels of CA125 in the interim analysis and the final analysis (*p* = 0.199; *p* = 0.185), nor was CA125 itself associated with response (*p* = 0.187; *p* = 0.205). A more rapid change in CA125 levels was observed between the screening visit (baseline) and the first follow-up visit post-treatment in patients in the ChemoID-guided group (slope = −1003) than in patients in the physician-choice group (slope = −165) (Fig. 7).

### Exploratory analyses: ChemoID test predictions correlated with patients’ objective tumor responses

**Fig. 8:**
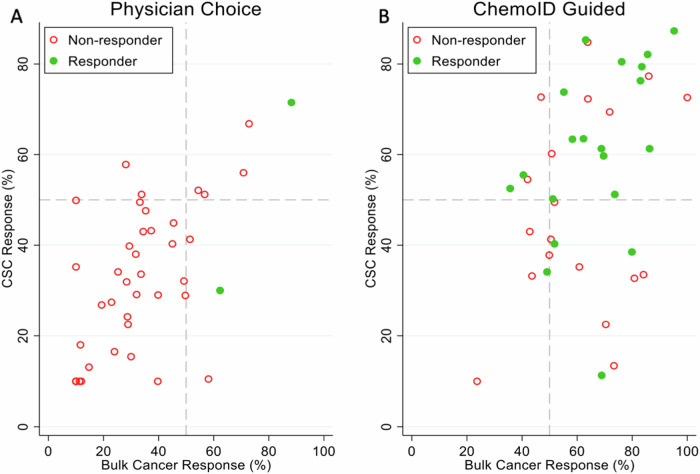
The patient’s response correlated with the cell kill of drugs used during treatment as per the ChemoID test report. **A** Quadrant diagrams of the associative analysis of cell kill percentages (bulk tumor cell and CSCs) vs tumor response post-treatment of subjects in the Physician-Choice group. Referent lines are drawn at 50% cell kill for the bulk of the tumor and CSCs, cluster subjects who had a response. Open red circles, participants who had no response (SD and PD); solid green circles, participants who had a response (PR and CR). **B** Quadrant diagrams of the associative analysis of cell kill percentages (bulk tumor cell and CSCs) vs tumor response post-treatment of subjects in the ChemoID-Guided group. Referent lines are drawn at 50% cell kill for the bulk of the tumor and CSCs, cluster subjects who had a response (PR and CR). Open red circles, participants who had no response (SD and PD); solid green circles, participants who had a response (PR and CR).

Response for each patient in the planned interim analysis was also analyzed as a function of the cell kill of the patient’s cultured tumor cells (both CSCs and bulk tumor cells) in response to the drug(s) used during treatment. Logistic regression models were constructed based on the ChemoID assay report data of patients’ cultured CSCs and bulk tumor cells exposed to the same drug(s) used during their treatment. We observed that the optimal thresholds of tumor cell kill were 50% for both CSCs and bulk tumor cells as per the logistic regression models (see referent lines in Fig. 8).

These thresholds are similar to our previously published data^30–32^. For patients in the physician-choice arm, data points were broadly distributed over both axes as expected given that treating physicians were blinded to the ChemoID data for patients in this arm. In striking contrast, most data points for patients in the ChemoID arm were clustered in the upper right quadrant (i.e., high percentage kill of both CSCs and bulk tumor cells).

### Association of response to the number of prior platinum treatments, age, race, and patients’ ECOG status

Both in the interim and final analyses, no association was found between response and age (*p* = 0.195; *p* = 0.250). In the same analyses, no association was found between response and the number of prior platinum-based treatments (*p* = 0.651; *p* = 0.493), ECOG status (*p* = 0.938; *p* = 0.812), or race (*p* = 0.173; 0.290) respectively (Tables 3, 4 and 5).

**Table 3:** Correlation between response and the number of prior platinum-based treatments

Interim Analysis	Physician-choice		ChemoID-Guided	
*p* = 0.651	No Response	Response	No Response	Response
**# of platinum treatments**				
1	20	2	8	9
2	13	0	5	6
3	2	0	2	2
4	4	0	0	2

Final Analysis	Physician-choice		ChemoID Guided	
*p* = 0.493	No Response	Response	No Response	Response
**# of platinum treatments**				
1	20	2	10	10
2	13	0	7	6
3	2	0	3	2
4	4	0	0	2

**Table 4:** Correlation between response and the ECOG status of the subjects at baseline

Interim Analysis	Physician-choice		ChemoID-Guided	
*p* = 0.938	No Response	Response	No Response	Response
ECOG				
0	5	0	2	2
1	33	2	18	18
2	1	0	0	0

Final Analysis	Physician-choice		ChemoID-Guided	
*p* = 0.812	No Response	Response	No Response	Response
ECOG				
0	5	0	1	2
1	33	2	14	17
2	1	0	0	0

**Table 5:** Correlation between response and race of subjects

Interim Analysis	Physician-choice		ChemoID-Guided	
*p* = 0.173	No Response	Response	No Response	Response
Race				
Asian	26	2	11	16
Black	1	0	0	0
Hispanic	3	0	0	1
White	9	0	4	2

Final Analysis	Physician-choice		ChemoID Guided	
*p* = 0.290	No Response	Response	No Response	Response
Race				
Asian	26	2	14	16
Black	1	0	0	0
Hispanic	3	0	1	1
White	9	0	5	3

**Fig. 9:**
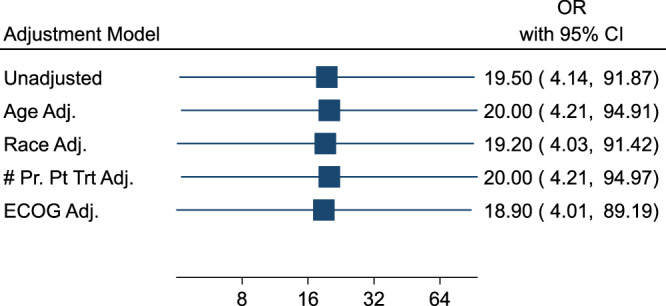
Forest Plot illustrates the lack of association between response and age, race, number of prior platinum treatments, and ECOG.

Due to low response counts in the physician-choice group, it was impossible to calculate odds ratios stratified by age, race, number of prior platinum-based treatments, or ECOG status. However, as a sensitivity analysis, we included each of these as an individual adjustment variable in a series of logistic regressions that also contained an indicator for physician-choice vs ChemoID-guided. The results, compared to an unadjusted model, are shown in Fig. 9. None of these variables had a modifying effect on the relationship between the treatment arm and response.

**Fig. 10:**
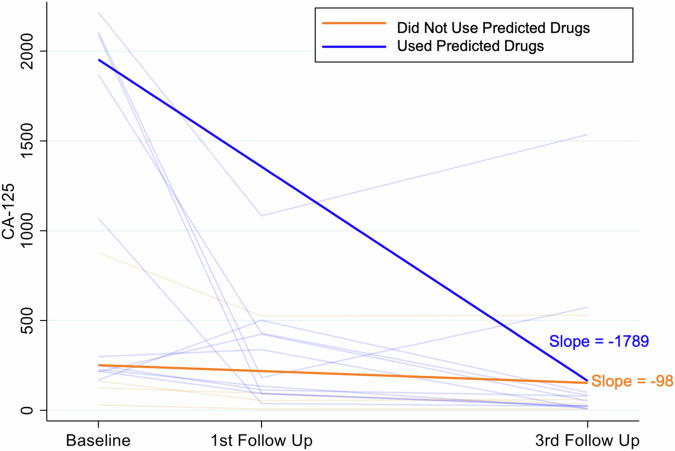
Levels of serum CA125 between screening and third follow-up are improved when subjects are treated with high-cell kill ChemoID-predicted therapy. High cell-kill drug cut-offs are above 50% cell kill for the bulk of the tumor and CSCs. Patients randomized in either of the arms were re-grouped into two groups depending on whether they were treated with high-cell kill predicted drugs or not. The CA125 serum levels of subjects treated using high cell kill predicted drugs vs. patients treated with drugs not predicted by the assay were plotted. The slope of the group of patients who were treated with assay-predicted (blue) vs. not-predicted drugs (red) is represented.

To better understand the impact of high-cell kill predicted drugs ( > 50% cell kill) on CA125 levels between screening visit (baseline) and the first follow-up visit post-treatment, we regrouped subjects from both arms who were treated with high-cell kill predicted drugs in one group and subjects from both arms who were treated with non-predicted drugs in the other group and determined the slope of change between CA125 levels in the screening visit (baseline) and the first follow-up visit post-treatment. We found that an even more rapid change in CA125 levels was observed between the screening visit (baseline) and the first follow-up visit post-treatment in patients treated with predicted drugs (slope = −1789) than patients treated with non-predicted drugs (slope = −98), (Fig. 10).

### Correlation of chemotherapy treatments administered with ChemoID test result predictions and percentage of change in tumor burden

**Fig. 11:**
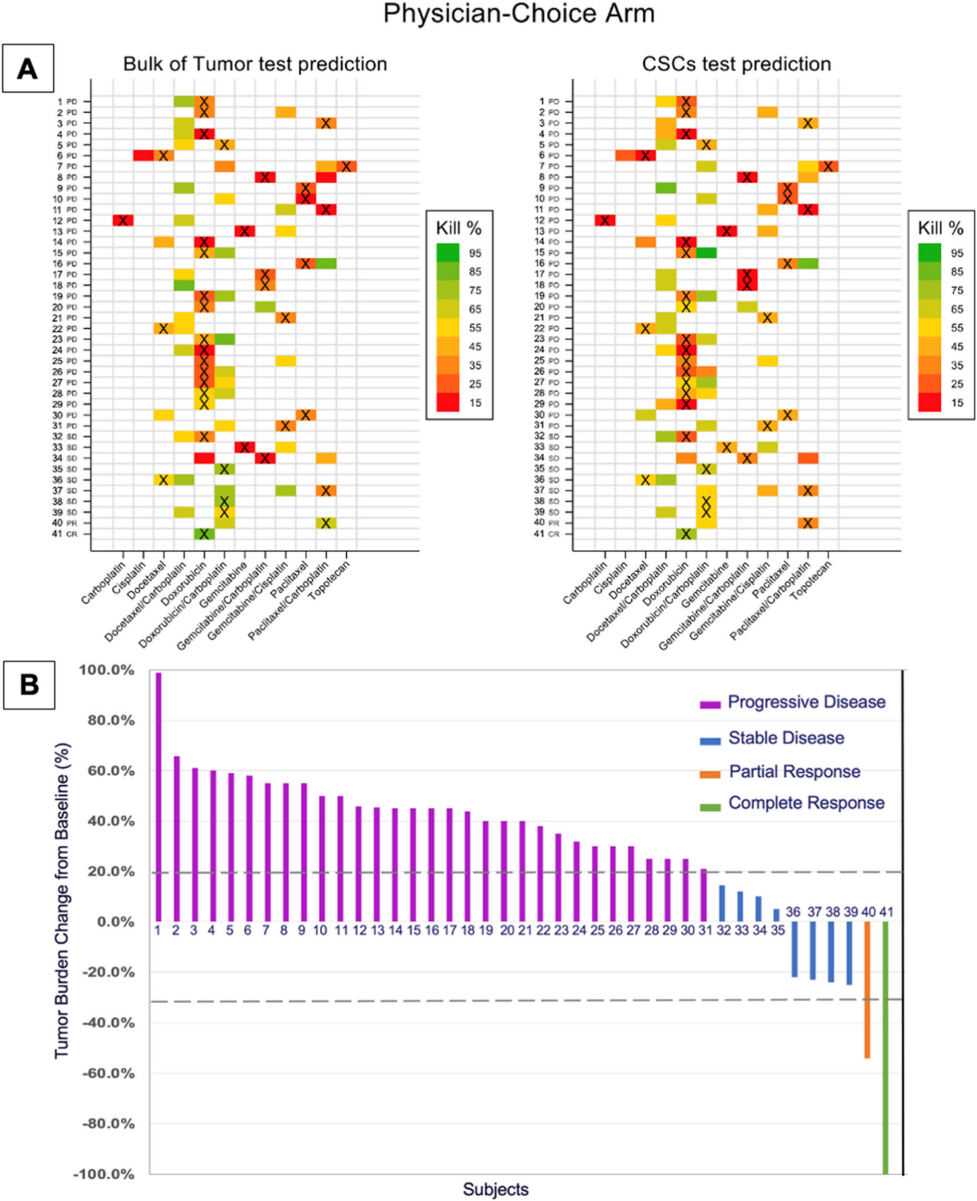
Heatmap of the drug response prediction and treatment received and percent of tumor burden change following therapy from subjects in the physician-choice arm. **A** Heat map of the Drug Response Prediction and Treatment Received. Each row represents a different participant in the Physician-Choice group. Each participant is labeled with a unique progressive number. Subjects labeled “NR” are non-responders (SD and PD), while those labeled “R” are responders (CR and PR) evaluated as per RECIST 1.1. The various drug/drug combinations used in the study are represented in the columns. The treating physicians were blinded to test results. The “X” represents the drug regimen administered to each subject. The color of the cells corresponds to the predicted cell kill % of the drug(s). The colored cells without the “X” show other potential drug regimens that were predicted, but not administered. **B** Percentage of tumor burden change as per RECIST 1.1 measurements following treatment received from subjects in the physician-choice group. Each column represents a different participant. Each participant is labeled with a unique progressive number. Referent lines are drawn to indicate complete response (CR, purple columns, 100% tumor decrease), partial response (PR, orange columns, ≥ 30% tumor decrease), stable disease (SD, blue columns, 30% tumor decrease to 20% tumor increase), and progressive disease (PD, purple columns, ≥ 20% tumor increase).

**Fig. 12:**
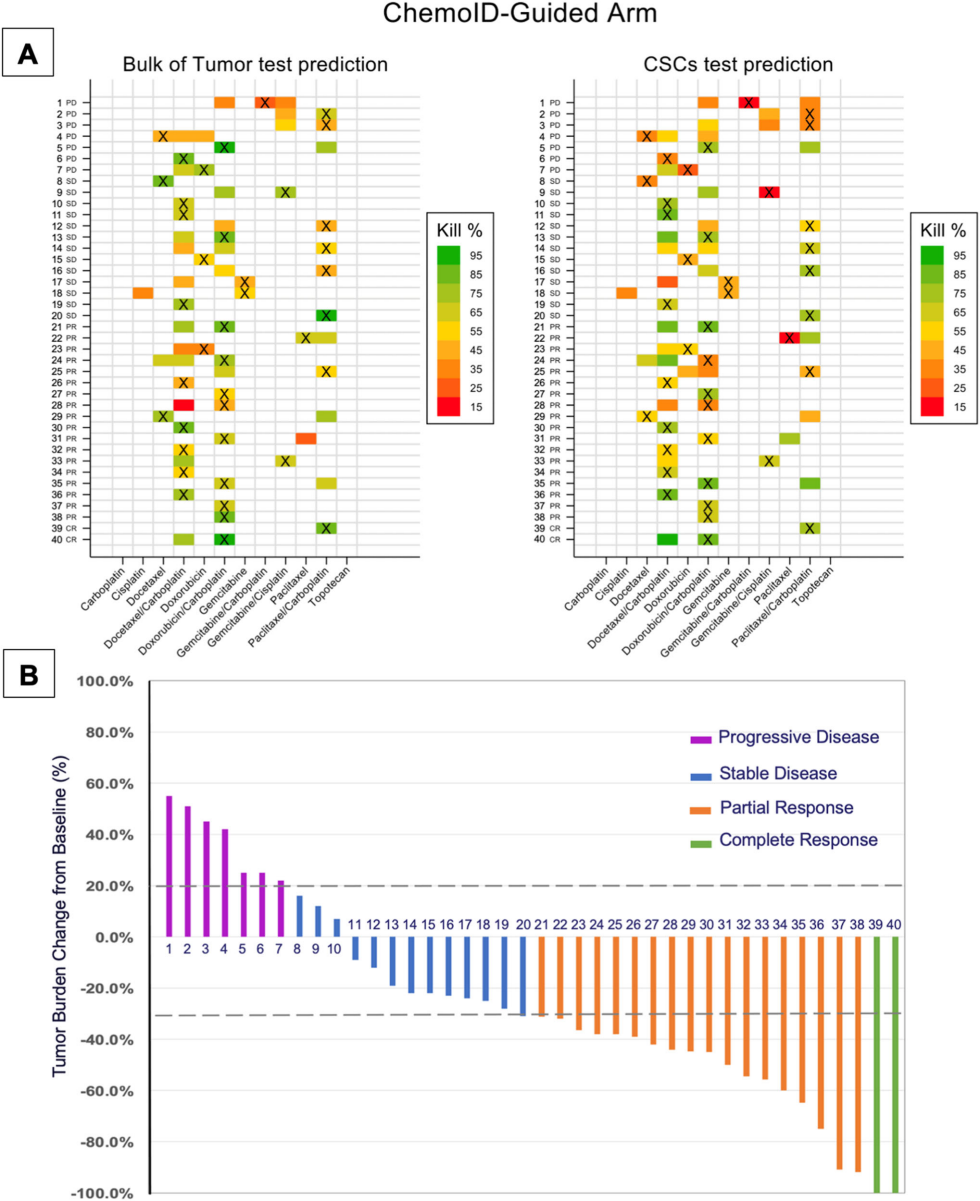
Heatmap of the drug response prediction and treatment received and percent of tumor burden change following therapy from subjects in the ChemoID-guided arm. **A** Heat map of the Drug Response Prediction and Treatment Received. Each row represents a different participant in the ChemoID-guided group. Each participant is labeled with a unique progressive number. Subjects labeled “NR” are non-responders (SD and PD), while those labeled “R” are responders (CR and PR) evaluated as per RECIST 1.1. The various drug/drug combinations used in the study are represented in the columns. Therapy was guided by the test results. The “X” represents the drug regimen administered to each subject. The color of the cells corresponds to the predicted cell kill % of the drug(s). The colored cells without the “X” show other potential drug regimens that were predicted, but not administered. **B** Percentage of tumor burden change as per RECIST 1.1 measurements following treatment received from subjects in the ChemoID-guided group. Each column represents a different participant. Each participant is labeled with a unique progressive number. Referent lines are drawn to indicate complete response (CR, purple columns, 100% tumor decrease), partial response (PR, orange columns, ≥ 30% tumor decrease), stable disease (SD, blue columns, 30% tumor decrease to 20% tumor increase), and progressive disease (PD, purple columns, ≥ 20% tumor increase).

The drug response to each chemotherapy and their combinations were analyzed to determine the proportion of patients who benefitted from a sensitive versus non-sensitive chemotherapy chosen prospectively by the ChemoID assay. A heatmap representation of the percent cell kill of the most cytotoxic drugs found by the ChemoID assay compared to the chemotherapy treatment used for each patient and the percent of change of tumor burden following therapy are shown in Figs. 11 and 12 for the physician-choice and ChemoID-guided groups, respectively. Each participant is labeled with a unique progressive number. Figures 11A and 12A, represent summarized information from the entire dataset presented in Tables 6 and 7. Subjects labeled “NR” are non-responders (SD and PD), while those labeled “R” are responders (CR and PR). The various drug/drug combinations used in the study are indicated in the columns (Figs. 11A and 12A). Optimal therapies with the highest cell kill found by the ChemoID assay are shown in shades of green-yellow colors and orange-red colors indicate low cell kill therapies. The “X” represents the drug regimen administered to each subject. Colored cells without the “X” show potential drug regimen(s) that were predicted but not administered.

**Table 6:** Physician-choice arm Predicted Drugs (treatment was administered blinded to test results)

Patient no.	Treatment received	ChemoID cytotoxic profile for Bulk of Tumor (high cell kill: > 50%; low cell kill: <50%)	ChemoID cytotoxic profile for CSCs (high cell kill: > 50%; low cell kill: <50%)	Response as per RECIST 1.1 criteria
1	Doxorubicin	Docetaxel/Carboplatin 78.7; Docetaxel 73.8; Doxorubicin/Carboplatin 61.6; Paclitaxel/Carboplatin 52.9; Gemcitabine/Cisplatin 40.1; Paclitaxel 32.9; Doxorubicin 32.1; Topotecan 31.7; Cisplatin 30.1; Gemcitabine/Carboplatin 24.0; Carboplatin 12.5; Gemcitabine <10	Docetaxel/Carboplatin 54.1; Doxorubicin/Carboplatin 51.2; Docetaxel 49.8; Paclitaxel/Carboplatin 45.0; Gemcitabine/Cisplatin 40.9; Paclitaxel 40.1; Doxorubicin 29.1; Cisplatin 19.8; Gemcitabine/Carboplatin 17.9; Topotecan 16.9; Carboplatin <10; Gemcitabine <10	PD
2	Doxorubicin	Gemcitabine/Cisplatin 46.6; Docetaxel/Carboplatin 41.6; Doxorubicin/Carboplatin 35.2; Cisplatin 33.3; Doxorubicin 31.7; Paclitaxel 27.3; Paclitaxel/Carboplatin 26.8; Gemcitabine/Carboplatin 21.2 Docetaxel 21.0; Gemcitabine 18.4; Carboplatin 16.5; Topotecan 10.3	Doxorubicin/Carboplatin 69.6; Docetaxel/Carboplatin 63.3; Paclitaxel/Carboplatin 59.4; Gemcitabine/Cisplatin 48.7; Cisplatin 41.3; Topotecan 39.4; Doxorubicin 38.9; Docetaxel 36.2; Paclitaxel 35.0; Gemcitabine/Carboplatin 27.2; Gemcitabine 12.5; Carboplatin <10	PD
3	Paclitaxel/Carboplatin	Docetaxel/Carboplatin 62.1; Docetaxel 61.4; Paclitaxel/Carboplatin 57.3; Paclitaxel 42.8; Doxorubicin/Carboplatin 42.7; Gemcitabine/Cisplatin 41.4; Cisplatin 36.5; Doxorubicin 30.3; Topotecan 22.7; Gemcitabine/Carboplatin 10.2; Carboplatin <10; Gemcitabine <10	Docetaxel/Carboplatin 45.8; Paclitaxel/Carboplatin 43.0; Paclitaxel 42.7; Gemcitabine/Cisplatin 32.1; Cisplatin 30.8; Doxorubicin 27.3; Docetaxel 27.1; Carboplatin 16.2; Gemcitabine 14.1; Doxorubicin/Carboplatin <10; Topotecan <10	PD
4	Doxorubicin	Docetaxel/Carboplatin 63.5; Gemcitabine/Cisplatin 56.0; Paclitaxel/Carboplatin 52.6; Docetaxel 46.0; Cisplatin 39.4; Topotecan 38.2 Doxorubicin/Carboplatin 35.7; Paclitaxel 34.5; Gemcitabine/Carboplatin 22.5; Carboplatin <10; Doxorubicin <10; Gemcitabine <10	Docetaxel/Carboplatin 42.6; Gemcitabine/Cisplatin 33.5; Paclitaxel/Carboplatin 31.2; Docetaxel 26.5; Paclitaxel/Carboplatin 22.3; Cisplatin 21.6; Topotecan 17.0; Doxorubicin/Carboplatin 15.7; Paclitaxel 12.0; Gemcitabine/Carboplatin <10; Carboplatin <10; Doxorubicin <10; Gemcitabine <10	PD
5	Doxorubicin/Carboplatin	Gemcitabine/Cisplatin 68.0; Doxorubicin 52.6; Docetaxel/Carboplatin 50.1; Cisplatin 49.8; Gemcitabine/Carboplatin 46.6; Doxorubicin/Carboplatin 45.0; Doxorubicin 45.0; Docetaxel 30.6; Paclitaxel/Carboplatin 28.8; Gemcitabine 26.5; Paclitaxel 21.8; Topotecan 10.4; Carboplatin <10	Docetaxel/Carboplatin 61.3; Doxorubicin 56.7; Paclitaxel 52.0; Doxorubicin 48.7; Gemcitabine 47.9; Paclitaxel/Carboplatin 47.5; Carboplatin 46.9; Doxorubicin/Carboplatin 40.3; Paclitaxel 39.8; Gemcitabine/Cisplatin 38.4; Docetaxel 37.1; Gemcitabine/Carboplatin 30.9; Cisplatin 28.0; Topotecan 18.6	PD
6	Docetaxel	Topotecan 45.1; Docetaxel/Carboplatin 43.4; Docetaxel 39.7; Paclitaxel 29.9; Paclitaxel/Carboplatin 27.9; Carboplatin 24.0; Doxorubicin 19.0; Carboplatin 16.4; Gemcitabine 16.3; Gemcitabine/Cisplatin 16.1; Doxorubicin/Carboplatin 14.9; Cisplatin 14.8; Gemcitabine/Carboplatin 12.1	Cisplatin 27.3; Doxorubicin 25.8; Paclitaxel/Carboplatin 11.2; Carboplatin <10; Paclitaxel <10; Docetaxel <10; Gemcitabine <10; Docetaxel/Carboplatin <10; Topotecan <10	PD
7	Gemcitabine/Carboplatin	Cisplatin 24.8; Docetaxel/Carboplatin 20.6; Gemcitabine/Cisplatin 19.8; Paclitaxel/Carboplatin 16.7; Doxorubicin 14.2; Doxorubicin/Carboplatin 14.1; Paclitaxel <10; Docetaxel <10; Gemcitabine <10; Carboplatin <10; Topotecan <10; Gemcitabine/Carboplatin <10	Doxorubicin/Carboplatin 49.4; Paclitaxel/Carboplatin 45.7; Docetaxel 41.6; Paclitaxel 36.7; Docetaxel/Carboplatin 33.3; Gemcitabine/Cisplatin 32.5; Paclitaxel 29.7; Topotecan 29.3; Cisplatin 25.1; Doxorubicin 16.5; Carboplatin 10.2; Gemcitabine 10.2; Gemcitabine/Carboplatin <10	PD
8	Topotecan	Paclitaxel/Carboplatin 47.1; Docetaxel/Carboplatin 33.3; Doxorubicin/Carboplatin 32.3; Gemcitabine/Cisplatin 25.2; Docetaxel 24.4; Topotecan 22.9; Gemcitabine/Carboplatin 18.1; Cisplatin 16.8; Carboplatin 14.1; Paclitaxel 11.3; Doxorubicin <10; Paclitaxel <10; Gemcitabine <10	Doxorubicin/Carboplatin 69.0; Docetaxel 59.9; Paclitaxel/Carboplatin 50.9; Doxorubicin 50.4; Cisplatin 42.4; Paclitaxel 32.7; Topotecan 27.4; Carboplatin 20.4	PD
9	Paclitaxel	Doxorubicin/Carboplatin 83.5; Doxorubicin 80.4; Gemcitabine/Cisplatin 79.9; Cisplatin 75.2; Docetaxel/Carboplatin 71.9; Docetaxel 61.4; Paclitaxel/Carboplatin 51.3; Gemcitabine/Carboplatin 45.3; Paclitaxel 28.7; Topotecan 24.2; Carboplatin 12.9; Gemcitabine <10	Docetaxel/Carboplatin 82.3; Docetaxel 81.4; Paclitaxel/Carboplatin 63.8; Doxorubicin/Carboplatin 61.9; Gemcitabine/Cisplatin 52.1; Topotecan 50.4; Cisplatin 44.8; Doxorubicin 41.2; Paclitaxel 29.4; Gemcitabine 10.5; Gemcitabine/Carboplatin 10.2; Carboplatin <10	PD
10	Paclitaxel/Carboplatin	Docetaxel/Carboplatin 76.7; Docetaxel 62.4; Gemcitabine/Cisplatin 62.0; Paclitaxel/Carboplatin 58.3; Gemcitabine/Carboplatin 51.3; Cisplatin 42.7; Doxorubicin/Carboplatin 38.1; Paclitaxel 35.2; Carboplatin 32.4; Doxorubicin 26.8; Topotecan <10; Gemcitabine <10	Gemcitabine/Cisplatin 48.4; Doxorubicin 45.2; Cisplatin 41.4; Paclitaxel 39.9; Carboplatin 39.8; Gemcitabine/Carboplatin 35.5; Gemcitabine 35.2; Docetaxel 27.0; Docetaxel/Carboplatin 23.1; Paclitaxel/Carboplatin 13.1	PD
11	Paclitaxel	Doxorubicin/Carboplatin 51.5; Paclitaxel/Carboplatin 49.1; Topotecan 48.3; Docetaxel/Carboplatin 45.7; Docetaxel 39.9; Gemcitabine/Cisplatin 37.7; Cisplatin 34.9; Gemcitabine/Carboplatin 30.1; Carboplatin 23.8; Gemcitabine 19.7; Paclitaxel 19.3; Carboplatin17.5; Doxorubicin 15.5	Docetaxel/Carboplatin 61.2; Doxorubicin/Carboplatin 60.4; Paclitaxel/Carboplatin 58.1; Topotecan 50.5; Docetaxel 46.7; Cisplatin 46.2; Gemcitabine/Cisplatin 45.5; Paclitaxel/Carboplatin 37.7; Doxorubicin 28.7; Paclitaxel 29.3; Gemcitabine/Carboplatin 27.6; Carboplatin 17.5; Gemcitabine 14.6	PD
12	Carboplatin	Gemcitabine/Cisplatin 68.0; Doxorubicin 62.6; Docetaxel/Carboplatin 60.5; Cisplatin 49.8; Gemcitabine/Carboplatin 45.6; Doxorubicin/Carboplatin 45.0; Docetaxel 27.3; Paclitaxel/Carboplatin 22.8; Gemcitabine 16.3; Paclitaxel 16.0; Topotecan 10.2; Carboplatin <10	Docetaxel/Carboplatin 55.3; Doxorubicin 52.3; Paclitaxel 50.1; Doxorubicin 42.3; Gemcitabine 40.1; Paclitaxel/Carboplatin 37.9; Carboplatin 38.2; Doxorubicin/Carboplatin 37.8; Paclitaxel 36.2; Gemcitabine/Cisplatin 29.3; Docetaxel 29.0; Gemcitabine/Carboplatin 27.8; Cisplatin 12.0; Carboplatin <10; Topotecan <10	PD
13	Gemcitabine	Gemcitabine/Cisplatin 51.6; Doxorubicin 37.9; Docetaxel 34.0; Cisplatin 30.8; Docetaxel/Carboplatin 29.9; Gemcitabine/Carboplatin 26.4; Carboplatin 18.8; Doxorubicin/Carboplatin 15.5; Paclitaxel 14.9; Paclitaxel/Carboplatin 13.7; Gemcitabine 11.3; Topotecan <10	Gemcitabine/Cisplatin 44.3; Doxorubicin 38.9; Docetaxel 37.1; Cisplatin 35.4; Docetaxel/Carboplatin 38.1; Gemcitabine/Carboplatin 30.0; Carboplatin 27.3; Doxorubicin/Carboplatin 22.2; Paclitaxel 11.4; Paclitaxel/Carboplatin 10.3; Gemcitabine <10; Topotecan <10	PD
14	Doxorubicin	Docetaxel 40.9; Paclitaxel/Carboplatin 12.7; Cisplatin 12.7; Doxorubicin 11.9; Paclitaxel 11.7; Carboplatin <10; Gemcitabine <10; Doxorubicin/Carboplatin <10	Docetaxel 31.2; Paclitaxel/Carboplatin 11.4; Cisplatin 11.0; Doxorubicin <10; Paclitaxel <10; Carboplatin <10; Gemcitabine <10; Doxorubicin/Carboplatin <10	PD
15	Doxorubicin	Docetaxel/Carboplatin 74.0; Doxorubicin/Carboplatin 73.9; Docetaxel 73.5; Doxorubicin 49.2; Gemcitabine/Cisplatin 39.9; Paclitaxel/Carboplatin 34.8; Cisplatin 34.4; Paclitaxel 18.9; Carboplatin 11.0; Gemcitabine/Carboplatin 11.0; Topotecan 10.4; Gemcitabine <10	Doxorubicin/Carboplatin 93.6; Paclitaxel/Carboplatin 91.5; Docetaxel/Carboplatin 89.7; Gemcitabine/Cisplatin 83.0; Cisplatin 72.0; Docetaxel 70.5; Gemcitabine/Carboplatin 59.9; Paclitaxel 55.3; Doxorubicin 32.1; Carboplatin 28.0; Gemcitabine 20.0; Topotecan 18.7; Carboplatin <10	PD
16	Paclitaxel	Doxorubicin/Carboplatin 92.4; Gemcitabine/Cisplatin 90.0; Paclitaxel/Carboplatin 89.4; Cisplatin 86.8; Doxorubicin 83.2; Gemcitabine/Carboplatin 70.6; Docetaxel/Carboplatin 69.9; Carboplatin 60.6; Docetaxel 47.4; Paclitaxel 25.3; Topotecan 18.6; Gemcitabine <10	Paclitaxel/Carboplatin 86.5; Gemcitabine/Cisplatin 83.2; Doxorubicin/Carboplatin 82.7; Cisplatin 77.8; Docetaxel/Carboplatin 72.0; Gemcitabine/Carboplatin 64.8; Doxorubicin 64.8; Docetaxel 58.3; Carboplatin 40.7; Paclitaxel 34.1; Topotecan 23.9; Gemcitabine <10	PD
17	Gemcitabine/Carboplatin	Docetaxel/Carboplatin 53.2; Carboplatin 43.1; Doxorubicin 41.4; Gemcitabine 39.4; Paclitaxel/Carboplatin 38.0; Gemcitabine/Cisplatin 34.2; Cisplatin 32.6; Paclitaxel 31.3; Doxorubicin/Carboplatin 28.0; Docetaxel 24.2; Gemcitabine/Carboplatin 24.0; Topotecan <10	Docetaxel/Carboplatin 60.8; Topotecan 52.6; Paclitaxel 48.6; Doxorubicin/Carboplatin 45.2; Paclitaxel/Carboplatin 44.6; Docetaxel 37.3; Doxorubicin 32.9; Gemcitabine/Cisplatin 32.4; Cisplatin 19.7; Gemcitabine/Carboplatin 16.5; Gemcitabine 10.6; Carboplatin <10	PD
18	Gemcitabine/Carboplatin	Docetaxel/Carboplatin 80.1; Docetaxel 70.0; Gemcitabine/Cisplatin 59.9; Doxorubicin/Carboplatin 51.8; Paclitaxel/Carboplatin 50.3; Doxorubicin 45.2; Paclitaxel 43.7; Cisplatin 38.4; Gemcitabine/Carboplatin 30.0; Paclitaxel 29.9; Gemcitabine 19.7; Carboplatin 15.4; Topotecan <10	Docetaxel /Carboplatin 62.4; Docetaxel 52.0; Paclitaxel/Carboplatin 39.7; Doxorubicin 37.1; Gemcitabine 28.9; Docetaxel 24.6; Topotecan 22.0; Paclitaxel 20.6; Gemcitabine/Carboplatin 15.4	PD
19	Doxorubicin	Gemcitabine/Cisplatin 89.0; Paclitaxel/Carboplatin 76.3; Cisplatin 70.4; Doxorubicin/Carboplatin 66.4; Docetaxel 61.6; Docetaxel/Carboplatin 60.1; Paclitaxel/Carboplatin 51.2; Gemcitabine/Carboplatin 49.7; Paclitaxel 49.5; Doxorubicin 33.8; Paclitaxel 26.1; Carboplatin 18.8; Topotecan 18.8; Gemcitabine <10	Doxorubicin/Carboplatin 68.7; Gemcitabine/Carboplatin 67.8; Cisplatin 58.0; Docetaxel 56.8; Paclitaxel/Carboplatin 44.8; Doxorubicin 51.2; Docetaxel/Carboplatin 46.2; Topotecan 44.3; Gemcitabine/Carboplatin 36.9; Paclitaxel 29.3; Gemcitabine 15.6; Carboplatin 15.5	PD
20	Doxorubicin	Doxorubicin/Carboplatin 76.6; Gemcitabine/Cisplatin 642; Docetaxel/Carboplatin 54.4; Cisplatin 42.2; Docetaxel 29.3; Doxorubicin 27.9; Doxorubicin 28.4; Paclitaxel/Carboplatin 27.8; Paclitaxel 19.0; Gemcitabine/Carboplatin 18.8; Paclitaxel 14.3; Carboplatin 10.7; Topotecan <10; Gemcitabine <10	Doxorubicin/Carboplatin 77.9; Cisplatin 62.7; Gemcitabine/Cisplatin 62.5; Docetaxel/Carboplatin 34.8; Doxorubicin 31.9; Docetaxel 26.9; Carboplatin 26.1; Gemcitabine 22.3	PD
21	Gemcitabine/Cisplatin	Docetaxel/Carboplatin 58.8; Doxorubicin/Carboplatin 41.5; Gemcitabine/Cisplatin 37.3; Paclitaxel/Carboplatin 32.2; Doxorubicin 29.7; Cisplatin 29.0; Gemcitabine/Carboplatin 26.5; Docetaxel 25.9; Doxorubicin 20.8; Paclitaxel 20.1; Topotecan 19.7; Carboplatin 13.6; Gemcitabine <10	Docetaxel/Carboplatin 64.3; Doxorubicin/Carboplatin 59.0; Paclitaxel/Carboplatin 53.4; Docetaxel 52.8; Topotecan 47.7; Gemcitabine/Cisplatin 43.2; Paclitaxel 36.1; Doxorubicin 30.4; Cisplatin 27.6; Gemcitabine/Carboplatin 19.5; Gemcitabine <10; Carboplatin <10	PD
22	Docetaxel	Docetaxel/Carboplatin 58.4; Gemcitabine/Cisplatin 51.0; Cisplatin 49.3; Docetaxel 45.4; Doxorubicin/Carboplatin 41.4; Paclitaxel/Carboplatin 35.1; Gemcitabine/Carboplatin 30.3 Topotecan 21.8 Doxorubicin 20.7; Paclitaxel 15.2; Carboplatin <10; Gemcitabine <10	Docetaxel/Carboplatin 69.1; Paclitaxel/Carboplatin 68.1; Gemcitabine/Cisplatin 62.5; Paclitaxel/Carboplatin 45.2; Docetaxel 44.9; Cisplatin 44.0; Doxorubicin/Carboplatin 43.9; Topotecan 40.5; Gemcitabine/Carboplatin 31.0; Paclitaxel 23.0; Doxorubicin 17.8; Gemcitabine 13.0; Carboplatin <10	PD
23	Doxorubicin	Doxorubicin/Carboplatin 89.9; Gemcitabine/Cisplatin 74.9; Cisplatin 74.9; Gemcitabine/Carboplatin 72.1; Paclitaxel/Carboplatin 61.0; Doxorubicin 49.7; Docetaxel/Carboplatin 45.4; Docetaxel 42.1; Paclitaxel 29.4; Topotecan 25.4; Carboplatin <10; Gemcitabine <10	Doxorubicin/Carboplatin 65.4; Gemcitabine/Cisplatin 62.3; Cisplatin 52.3; Gemcitabine/Carboplatin 38.6; Paclitaxel/Carboplatin 37.1; Doxorubicin 28.9; Docetaxel/Carboplatin 22.0; Docetaxel 20.6; Paclitaxel/Carboplatin 20.1; Paclitaxel 18.3; Topotecan <10; Carboplatin <10; Gemcitabine <10	PD
24	Doxorubicin	Docetaxel/Carboplatin 65.2; Doxorubicin/Carboplatin 52.5; Docetaxel 49.7; Paclitaxel/Carboplatin 49.3; Topotecan 41.0; Paclitaxel 29.9; Gemcitabine/Cisplatin 28.6; Paclitaxel/Carboplatin 20.9; Cisplatin 20.8; Doxorubicin 14.6; Paclitaxel 11.6; Gemcitabine/Carboplatin <10; Gemcitabine <10; Carboplatin <10	Docetaxel/Carboplatin 52.9; Docetaxel 50.4; Doxorubicin/Carboplatin 47.2; Paclitaxel/Carboplatin 43.4; Paclitaxel 32.8; Topotecan 23.0; Doxorubicin 19.4; Gemcitabine/Cisplatin 18.7; Cisplatin 14.4; Gemcitabine/Carboplatin <10; Gemcitabine <10; Carboplatin <10	PD
25	Doxorubicin	Doxorubicin/Carboplatin 55.7; Docetaxel 53.0; Docetaxel/Carboplatin 49.8; Paclitaxel/Carboplatin 47.1; Paclitaxel 39.2; Topotecan 36.1; Gemcitabine/Cisplatin 28.9; Doxorubicin 28.1; Cisplatin 26.5; Gemcitabine/Carboplatin 11.2; Carboplatin <10; Gemcitabine <10	Doxorubicin/Carboplatin 71.0; Gemcitabine/Cisplatin 64.5; Docetaxel/Carboplatin 60.8; Doxorubicin 60.7; Docetaxel 50.6; Paclitaxel 49.4; Cisplatin 42.9; Paclitaxel/Carboplatin 40.8; Gemcitabine/Carboplatin 19.6; Carboplatin 12.7; Gemcitabine 10.5; Topotecan <10	PD
26	Doxorubicin	Doxorubicin/Carboplatin 63.4; Gemcitabine/Cisplatin 62.3; Cisplatin 50.6; Gemcitabine/Carboplatin 38.6; Paclitaxel/Carboplatin 37.1; Doxorubicin 28.9; Docetaxel/Carboplatin 22.0; Docetaxel 20.6; Paclitaxel 18.3; Topotecan <10; Carboplatin <10; Gemcitabine <10	Docetaxel/Carboplatin 60.5; Paclitaxel/Carboplatin 56.0; Carboplatin 47.6; Gemcitabine 40.0; Doxorubicin/Carboplatin 39.1; Gemcitabine/Cisplatin 38.6; Docetaxel 37.2; Doxorubicin 22.5; Cisplatin 15.0; Paclitaxel 13.5; Gemcitabine/Carboplatin 13.0; Topotecan <10	PD
27	Doxorubicin	Gemcitabine/Cisplatin 52.2; Cisplatin 46.6; Paclitaxel/Carboplatin 43.6; Gemcitabine/Carboplatin 42.0; Docetaxel/Carboplatin 39.1; Doxorubicin/Carboplatin 36.9; Paclitaxel 35.5; Docetaxel 34.7; Doxorubicin 29.4; Gemcitabine 26.1; Carboplatin 24.6; Topotecan 15.0	Gemcitabine/Cisplatin 55.9; Docetaxel/Carboplatin 54.8; Docetaxel 45.2; Paclitaxel/Carboplatin 44.8; Cisplatin 42.1; Doxorubicin/Carboplatin 41.2; Doxorubicin 39.8; Gemcitabine/Carboplatin 36.4; Gemcitabine 35.3; Paclitaxel 33.1; Topotecan 24.9; Carboplatin 10.5	PD
28	Doxorubicin	Docetaxel/Carboplatin 73.5; Docetaxel 67.2; Doxorubicin/Carboplatin 66.4; Doxorubicin 58.1; Gemcitabine/Cisplatin 53.5; Cisplatin 38.4; Paclitaxel/Carboplatin 27.8; Topotecan 18.6; Gemcitabine/Carboplatin 11.7; Carboplatin <10; Gemcitabine <10; Paclitaxel <10	Docetaxel/Carboplatin 48.6; Docetaxel 40.8; Doxorubicin 10.5; Paclitaxel/Carboplatin <10; Carboplatin <10; Gemcitabine <10; Doxorubicin/Carboplatin <10; Topotecan <10; Paclitaxel <10; Cisplatin <10; Carboplatin <10	PD
29	Paclitaxel	Doxorubicin/Carboplatin 57.8; Docetaxel/Carboplatin 56.6; Docetaxel 56.1; Paclitaxel/Carboplatin 47.5; Gemcitabine/Cisplatin 41.1; Doxorubicin 37.5; Topotecan 33.8; Paclitaxel 33.2; Doxorubicin30.7; Cisplatin 25.5; Gemcitabine/Carboplatin 20.7; Carboplatin 12.6; Gemcitabine <10	Docetaxel 63.9; Paclitaxel/Carboplatin 60.3; Topotecan 57.5; Doxorubicin/Carboplatin 56.2; Docetaxel/Carboplatin 50.9; Paclitaxel 49.5; Gemcitabine/Cisplatin 27.1; Cisplatin 26.5; Doxorubicin 25.6; Carboplatin 16.8; Gemcitabine/Carboplatin <10; Gemcitabine <10	PD
30	Doxorubicin	Docetaxel/Carboplatin 73.0; Gemcitabine/Cisplatin 72.7; Docetaxel 68.2; Cisplatin 63.9; Doxorubicin/Carboplatin 61.6; Doxorubicin 51.4; Paclitaxel/Carboplatin 35.3; Gemcitabine/Carboplatin 26.4; Paclitaxel 25.7; Carboplatin <10; Gemcitabine <10; Topotecan <10	Doxorubicin/Carboplatin 57.3; Docetaxel/Carboplatin 47.4; Cisplatin 42.6; Doxorubicin 41.3; Gemcitabine/Cisplatin 38.1; Paclitaxel/Carboplatin 32.0; Topotecan 31.3; Docetaxel 22.2; Gemcitabine/Carboplatin 11.4; Paclitaxel 10.3; Carboplatin <10; Gemcitabine <10	PD
31	Gemcitabine/Cisplatin	Doxorubicin/Carboplatin 54.2; Docetaxel/Carboplatin 52.2; Paclitaxel/Carboplatin 48.8; Docetaxel 41.0; Gemcitabine/Cisplatin 35.3; Paclitaxel 33.5; Cisplatin 30.6; Gemcitabine/Carboplatin 29.9; Topotecan 22.5; Carboplatin 21.4; Doxorubicin <10; Gemcitabine <10	Doxorubicin/Carboplatin 65.3; Gemcitabine/Cisplatin 47.6; Paclitaxel/Carboplatin 44.5; Docetaxel/Carboplatin 40.2; Cisplatin 35.9; Doxorubicin 26.0; Gemcitabine/Carboplatin 18.3; Paclitaxel 14.6; Docetaxel 12.6; Carboplatin <10; Gemcitabine <10	PD
32	Doxorubicin	Docetaxel/Carboplatin 59.6; Docetaxel 54.9; Doxorubicin 39.8; Gemcitabine/Cisplatin 35.5; Doxorubicin/Carboplatin 32.2; Paclitaxel/Carboplatin <10; Cisplatin <10; Paclitaxel <10; Gemcitabine <10; Carboplatin <10; Gemcitabine/Carboplatin <10; Topotecan <10	Docetaxel/Carboplatin 74.0; Paclitaxel/Carboplatin 60.4; Paclitaxel 47.4; Docetaxel 41.6; Doxorubicin/Carboplatin 41.4; Gemcitabine/Cisplatin 35.7; Cisplatin 32.9; Topotecan 32.2; Doxorubicin 29.0; Gemcitabine/Carboplatin 16.6; Carboplatin <10	SD
33	Gemcitabine	Gemcitabine/Cisplatin 57.7; Docetaxel/Carboplatin 55.9; Doxorubicin/Carboplatin 53.7; Cisplatin 45.5; Paclitaxel/Carboplatin 37.0; Docetaxel 29.6; Gemcitabine/Carboplatin 27.7; Doxorubicin 27.7; Carboplatin 17.1; Topotecan 13.1; Paclitaxel 11.2; Gemcitabine <10	Gemcitabine/Cisplatin 61.0; Doxorubicin/Carboplatin 55.2; Cisplatin 54.0; Docetaxel/Carboplatin 51.6; Gemcitabine 49.9; Doxorubicin 49.5; Gemcitabine/Carboplatin 39.9; Carboplatin 38.0; Docetaxel 32.3; Paclitaxel 31.6; Paclitaxel/Carboplatin 30.8; Topotecan 17.8	SD
34	Gemcitabine/Carboplatin	Paclitaxel/Carboplatin 48.6; Carboplatin 42.7; Paclitaxel 22.5; Docetaxel/Carboplatin 20.3; Doxorubicin 18.6; Cisplatin 16.7; Gemcitabine 13.8; Docetaxel <10; Gemcitabine/Carboplatin <10; Doxorubicin/Carboplatin <10; Gemcitabine/Cisplatin <10; Topotecan <10	Doxorubicin 35.5; Gemcitabine/Cisplatin 35.2; Paclitaxel 29.0; Paclitaxel/Carboplatin 21.9; Cisplatin 20.9; Docetaxel/Carboplatin 20.8; Carboplatin 17.7; Docetaxel 12.8; Gemcitabine 10.2; Gemcitabine/Carboplatin <10	SD
35	Doxorubicin/Carboplatin	Doxorubicin/Carboplatin 72.8; Doxorubicin 57.8; Paclitaxel/Carboplatin 53.8; Gemcitabine/Cisplatin 52.3; Docetaxel 45.6; Topotecan 45.5; Cisplatin 44.7; Paclitaxel/Carboplatin 44.4; Paclitaxel 34.6; Docetaxel/Carboplatin 33.4; Gemcitabine/Carboplatin 27.8; Carboplatin <10; Gemcitabine <10	Doxorubicin/Carboplatin 66.8; Doxorubicin 52.4; Paclitaxel/Carboplatin 48.9; Gemcitabine/Cisplatin 42.1; Docetaxel 36.4; Topotecan 30.0; Cisplatin 29.5; Paclitaxel/Carboplatin 25.6; Paclitaxel 21.0; Docetaxel/Carboplatin 15.3; Gemcitabine/Carboplatin 12.4; Carboplatin <10; Gemcitabine <10	SD
36	Docetaxel	Docetaxel/Carboplatin 74.9; Doxorubicin 65.0; Paclitaxel/Carboplatin 65.0; Doxorubicin 64.9; Paclitaxel/Carboplatin 64.8; Carboplatin 61.9; Doxorubicin/Carboplatin 60.7; Gemcitabine/Cisplatin 59.7; Docetaxel 56.7; Gemcitabine 56.5; Paclitaxel 54.4; Cisplatin 50.7; Gemcitabine/Carboplatin 39.7; Topotecan 37.3	Docetaxel/Carboplatin 70.3; Doxorubicin 65.0; Paclitaxel/Carboplatin 60.3; Doxorubicin 59.6; Paclitaxel/Carboplatin 58.4; Carboplatin 57.5; Doxorubicin/Carboplatin 52.3; Gemcitabine/Cisplatin 51.6; Docetaxel 51.3; Gemcitabine 33.4; Paclitaxel 32.1; Cisplatin 30.0; Gemcitabine/Carboplatin 19.7; Topotecan 17.3	SD
37	Paclitaxel/Carboplatin	Gemcitabine/Cisplatin 78.8; Doxorubicin 78.7; Doxorubicin/Carboplatin 75.9; Docetaxel /Carboplatin 75.1; Cisplatin 63.5; Gemcitabine/Carboplatin 42.6; Gemcitabine 40.4; Paclitaxel/Carboplatin 33.6; Docetaxel 33.2; Carboplatin 32.6; Paclitaxel 27.4; Topotecan <10	Doxorubicin/Carboplatin 58.5; Paclitaxel/Carboplatin 53.8; Docetaxel/Carboplatin 48.2; Gemcitabine/Cisplatin 46.9; Cisplatin 44.5; Docetaxel 35.4; Paclitaxel 33.8; Gemcitabine/Carboplatin 32.3; Doxorubicin 31.9; Topotecan 26.3; Carboplatin 22.1; Gemcitabine <10	SD
38	Doxorubicin/Carboplatin	Doxorubicin/Carboplatin 70.8; Docetaxel/Carboplatin 64.4; Docetaxel 61.3; Gemcitabine/Cisplatin 58.0; Paclitaxel/Carboplatin 56.1; Paclitaxel 50.8; Cisplatin 48.2; Doxorubicin 47.9; Paclitaxel/Carboplatin 40.5; Gemcitabine/Carboplatin 39.3; Topotecan 39.1; Carboplatin 29.2; Gemcitabine 23.0	Doxorubicin/Carboplatin 70.8; Docetaxel/Carboplatin 54.9; Docetaxel 49.1; Paclitaxel/Carboplatin 45.0; Doxorubicin 37.6; Gemcitabine/Cisplatin 28.4; Topotecan 27.0; Paclitaxel 23.2; Cisplatin 20.5; Carboplatin 10.4; Gemcitabine <10; Gemcitabine/Carboplatin <10	SD
39	Doxorubicin/Carboplatin	Docetaxel/Carboplatin 63.4; Doxorubicin 59.9; Paclitaxel/Carboplatin 58.7; Carboplatin 55.2; Doxorubicin/Carboplatin 54.5; Gemcitabine/Cisplatin 45.9; Docetaxel 40.3; Gemcitabine 38.4; Paclitaxel 27.4; Cisplatin 25.7; Gemcitabine/Carboplatin 19.7; Topotecan 17.3	Docetaxel/Carboplatin 62.3; Doxorubicin 56.0; Paclitaxel/Carboplatin 52.3; Paclitaxel/Carboplatin 58.4; Carboplatin 57.5; Doxorubicin/Carboplatin 52.1; Gemcitabine/Cisplatin 51.6; Docetaxel 51.2; Gemcitabine 33.4; Paclitaxel 32.1; Cisplatin 30.0; Gemcitabine/Carboplatin 19.9; Topotecan 17.3	SD
40	Paclitaxel/Carboplatin	Paclitaxel/Carboplatin 62.3; Doxorubicin/Carboplatin 61.2; Gemcitabine/Cisplatin 60.6; Docetaxel 55.0; Paclitaxel 54.8; Cisplatin 53.7; Doxorubicin 50.1; Carboplatin 30.1; Topotecan 23.4; Gemcitabine 20.8; Gemcitabine/Carboplatin <10	Doxorubicin/Carboplatin 50.3; Gemcitabine/Cisplatin 38.9; Docetaxel 37.1; Paclitaxel 35.4; Cisplatin 38.1; Paclitaxel/Carboplatin 30.0; Doxorubicin 27.3; Carboplatin 22.2; Topotecan <10; Gemcitabine <10; Gemcitabine/Carboplatin <10	PR
41	Doxorubicin	Doxorubicin/Carboplatin 91.7; Doxorubicin 88.2; Gemcitabine/Cisplatin 80.9; Cisplatin 70.3; Paclitaxel/Carboplatin 61.4; Gemcitabine/Carboplatin 54.8; Docetaxel/Carboplatin 51.6; Docetaxel 46.1; Paclitaxel 43.6; Paclitaxel/Carboplatin 33.6; Topotecan 31.4; Gemcitabine 20.0; Carboplatin <10	Doxorubicin/Carboplatin 96.1; Docetaxel/Carboplatin 79.5; Paclitaxel/Carboplatin 79.1; Doxorubicin 71.5; Paclitaxel 64.3; Docetaxel 63.1; Gemcitabine/Cisplatin 61.7; Paclitaxel/Carboplatin 48.5; Topotecan 38.7; Cisplatin 34.4; Carboplatin 20.0; Gemcitabine 11.9; Carboplatin <10; Gemcitabine/Carboplatin <10	CR

**Table 7:** ChemoID-guided arm Predicted Drugs (treatment was guided by the test results)

Patient no.	Treatment received	ChemoID cytotoxic profile for Bulk of Tumor (high cell kill: > 50%; low cell kill: <50%)	ChemoID cytotoxic profile for CSCs (high cell kill: > 50%; low cell kill: <50%)	Response as per RECIST 1.1 criteria
1	Gemcitabine/Carboplatin	Gemcitabine/Cisplatin 33.3; Doxorubicin/Carboplatin 32.4; Cisplatin 30.9; Doxorubicin29.1; Gemcitabine 27.9; Carboplatin 25.7; Gemcitabine/Carboplatin 23.7; Paclitaxel/Carboplatin 23.7; Paclitaxel 15.4; Docetaxel/Carboplatin 13.2; Topotecan 13.2; Docetaxel <10; Carboplatin <10	Paclitaxel/Carboplatin 39.7; Doxorubicin/Carboplatin 33.4; Paclitaxel 32.0; Cisplatin 31.0; Docetaxel/Carboplatin 30.8; Docetaxel 29.7; Gemcitabine/Cisplatin 26.5; Topotecan 23.6; Doxorubicin 16.2; Carboplatin 10.6; Gemcitabine <10; Gemcitabine/Carboplatin <10	PD
2	Paclitaxel/Carboplatin	Paclitaxel/Carboplatin 60.8; Docetaxel/Carboplatin54.0; Cisplatin 51.7; Gemcitabine/Cisplatin 49.9; Doxorubicin/Carboplatin 41.2; Paclitaxel 38.7; Docetaxel 38.2; Gemcitabine/Carboplatin 36.0; Gemcitabine 26.6; Doxorubicin 26.1; Carboplatin23.4; Topotecan 11.2	Gemcitabine/Cisplatin 41.9; Paclitaxel/Carboplatin 35.2; Doxorubicin/Carboplatin 20.9; Docetaxel/Carboplatin 20.7; Docetaxel <10; Doxorubicin <10; Paclitaxel <10; Cisplatin Gemcitabine/Carboplatin <10; Carboplatin <10; Topotecan <10; Gemcitabine <10	PD
3	Paclitaxel/Carboplatin	Gemcitabine/Cisplatin 51.0; Paclitaxel/Carboplatin 50.5; Docetaxel 42.9; Doxorubicin/Carboplatin 39.3; Paclitaxel 37.3; Cisplatin 35.7; Docetaxel/Carboplatin 34.6; Doxorubicin 29.2; Gemcitabine/Carboplatin 26.8; Topotecan 24.0; Carboplatin 11.5; Gemcitabine <10	Doxorubicin/Carboplatin 59.6; Paclitaxel/Carboplatin 37.8; Docetaxel 32.5; Paclitaxel/Carboplatin 31.1; Gemcitabine/Cisplatin 31.1; Topotecan 28.4; Docetaxel/Carboplatin 26.5; Doxorubicin 21.5; Paclitaxel 19.9; Cisplatin 17.9; Carboplatin <10; Gemcitabine <10; Gemcitabine/Carboplatin <10	PD
4	Docetaxel	Doxorubicin 48.5; Docetaxel/Carboplatin 46.5; Docetaxel 43.6; Paclitaxel/Carboplatin 43.1; Gemcitabine 41.0; Carboplatin 38.2; Cisplatin 22.8; Paclitaxel 21.4; Doxorubicin/Carboplatin 16.4; Gemcitabine/Carboplatin <10; Topotecan <10; Gemcitabine/Cisplatin <10	Docetaxel/Carboplatin 58.4; Doxorubicin/Carboplatin 47.2; Paclitaxel/Carboplatin 46.9; Paclitaxel 37.9; Doxorubicin 35.4; Docetaxel 33.2; Carboplatin 32.8; Gemcitabine 23.9	PD
5	Docetaxel/Carboplatin	Docetaxel/Carboplatin 80.8; Docetaxel 69.5; Doxorubicin/Carboplatin 37.5; Gemcitabine/Cisplatin 35.5; Cisplatin 33.8; Doxorubicin 28.2; Gemcitabine/Carboplatin 24.9; Gemcitabine 20.7; Paclitaxel/Carboplatin 19.7; Carboplatin <10; Topotecan <10; Paclitaxel <10	Docetaxel/Carboplatin 32.7; Doxorubicin/Carboplatin 29.3; Docetaxel 28.5; Gemcitabine/Cisplatin 22.6; Cisplatin 23.8; Doxorubicin 15.3; Gemcitabine/Carboplatin 12.0; Doxorubicin 10.9; Gemcitabine <10; Paclitaxel/Carboplatin <10; Carboplatin <10; Topotecan <10; Paclitaxel <10	PD
6	Doxorubicin/Carboplatin	Doxorubicin/Carboplatin 95.4; Doxorubicin 89.3; Gemcitabine/Cisplatin 88.9; Paclitaxel 74.9; Paclitaxel/Carboplatin 73.8; Docetaxel/Carboplatin 72.8; Docetaxel 70.6; Cisplatin 69.5; Gemcitabine/Carboplatin 58.9; Topotecan 58.5; Carboplatin 29.4; Gemcitabine 20.8	Doxorubicin/Carboplatin 75.8; Paclitaxel/Carboplatin 74.3; Gemcitabine/Cisplatin 73.4; Docetaxel/Carboplatin 67.4; Paclitaxel 61.9; Doxorubicin 55.4; Cisplatin 48.6; Gemcitabine/Carboplatin 26.0; Docetaxel 24.9; Gemcitabine 13.8; Carboplatin <10; Topotecan <10	PD
7	Doxorubicin	Doxorubicin/Carboplatin 85.1; Gemcitabine/Cisplatin 71.1; Doxorubicin 70.4; Cisplatin 61.3; Docetaxel/Carboplatin 60.6; Paclitaxel/Carboplatin 56.1; Docetaxel 46.0; Topotecan 42.3; Paclitaxel 31.3; Carboplatin 10.1; Gemcitabine/Carboplatin <10; Gemcitabine <10	Docetaxel/Carboplatin 60.5; Paclitaxel/Carboplatin 56.0; Carboplatin 47.6; Gemcitabine 40.0; Doxorubicin/Carboplatin 39.1; Gemcitabine/Cisplatin 38.6; Docetaxel 37.2; Doxorubicin 22.5; Cisplatin 17.2; Paclitaxel 15.3; Gemcitabine/Carboplatin 12.9; Topotecan <10	PD
8	Docetaxel	Docetaxel/Carboplatin 92.2; Docetaxel 84.1; Paclitaxel/Carboplatin 76.8; Paclitaxel 61.3; Doxorubicin 53.6; Cisplatin 52.0; Topotecan 50.9; Doxorubicin/Carboplatin 47.8; Gemcitabine/Cisplatin 47.0; Carboplatin 29.5; Gemcitabine/Carboplatin 25.2; Gemcitabine 11.5	Topotecan 39.3; Paclitaxel/Carboplatin 38.9; Docetaxel/Carboplatin 38.7; Docetaxel 33.5; Paclitaxel 22.6; Doxorubicin/Carboplatin 16.0; Cisplatin 11.0; Gemcitabine/Cisplatin <10; Doxorubicin <10; Carboplatin <10; Gemcitabine <10; Gemcitabine/Carboplatin <10	SD
9	Gemcitabine/Cisplatin	Doxorubicin/Carboplatin 77.7; Gemcitabine/Cisplatin 73.4; Docetaxel/Carboplatin 73.3; Doxorubicin 69.8; Cisplatin 68.9; Paclitaxel/Carboplatin 63.1; Docetaxel 58.9; Gemcitabine/Carboplatin 57.5; Topotecan 55.0; Paclitaxel 33.3; Carboplatin 11.6; Gemcitabine 10.8	Doxorubicin/Carboplatin 79.7; Docetaxel/Carboplatin 77.7; Docetaxel 72.1; Cisplatin 67.1; Doxorubicin 65.2; Paclitaxel/Carboplatin 65.1; Gemcitabine 50.3; Carboplatin 49.1; Paclitaxel 48.6; Topotecan 45.5; Gemcitabine/Carboplatin 20.3; Gemcitabine/Cisplatin 13.4	SD
10	Docetaxel/Carboplatin	Docetaxel/Carboplatin 63.9; Gemcitabine/Cisplatin 48.3; Paclitaxel/Carboplatin 44.7; Paclitaxel 29.3; Docetaxel 25.0; Doxorubicin/Carboplatin 16.0; Cisplatin 12.3; Gemcitabine 11.9; Gemcitabine/Carboplatin 11.8; Doxorubicin <10; Topotecan <10; Carboplatin <10	Docetaxel/Carboplatin 72.3; Gemcitabine/Cisplatin 70.7; Paclitaxel/Carboplatin 60.8; Carboplatin 56.8; Cisplatin 54.9; Gemcitabine/Carboplatin 54.6; Doxorubicin 52.7; Gemcitabine 52.4; Doxorubicin/Carboplatin 48.1; Docetaxel 45.1; Paclitaxel 43.9; Topotecan 42.1	SD
11	Docetaxel/Carboplatin	Docetaxel/Carboplatin 63.8 Doxorubicin 61.8; Doxorubicin/Carboplatin 59.9; Docetaxel 55.9; Gemcitabine/Cisplatin 52.3; Paclitaxel/Carboplatin 51.2; Topotecan 47.6; Paclitaxel 38.1; Cisplatin 26.5; Gemcitabine/Carboplatin 16.2; Gemcitabine <10; Carboplatin <10	Docetaxel/Carboplatin 84.8; Doxorubicin/Carboplatin 75.1; Gemcitabine/Cisplatin 67.5; Docetaxel 66.3; Cisplatin 62.3; Paclitaxel/Carboplatin 55.1; Paclitaxel 39.1; Doxorubicin 33.5; Gemcitabine/Carboplatin 30.1; Carboplatin 23.8; Gemcitabine <10; Topotecan <10	SD
12	Paclitaxel/Carboplatin	Doxorubicin/Carboplatin 46.9; Paclitaxel/Carboplatin 42.0; Docetaxel 36.6; Docetaxel/Carboplatin 33.9; Gemcitabine/Cisplatin 31.0; Topotecan 17.9; Paclitaxel <10; Doxorubicin <10; Gemcitabine/Carboplatin <10; Cisplatin <10; Gemcitabine <10; Carboplatin <10	Paclitaxel/Carboplatin 54.5; Doxorubicin/Carboplatin 48.3; Docetaxel 47.1; Docetaxel/Carboplatin 40.9; Paclitaxel/Carboplatin 33.2; Topotecan 26.5; Paclitaxel 25.3; Doxorubicin 18.8; Gemcitabine/Cisplatin 18.0; Cisplatin <10; Gemcitabine/Carboplatin <10; Gemcitabine <10; Carboplatin <10	SD
13	Doxorubicin/Carboplatin	Doxorubicin/Carboplatin 86.0; Paclitaxel/Carboplatin 74.5; Doxorubicin 70.5; Docetaxel 703; Docetaxel/Carboplatin 62.2; Gemcitabine/Cisplatin 52.8; Cisplatin 40.1; Paclitaxel 28.4; Gemcitabine/Carboplatin 23.1; Topotecan 21.1; Carboplatin <10; Gemcitabine <10	Docetaxel/Carboplatin 81.1; Doxorubicin/Carboplatin 77.3; Doxorubicin 68.3; Docetaxel 50.5; Gemcitabine/Cisplatin 50.3; Paclitaxel/Carboplatin 43.6; Cisplatin 38.1; Paclitaxel 37.4; Topotecan 36.7; Gemcitabine/Carboplatin 10.8; Gemcitabine <10; Carboplatin <10	SD
14	Doxorubicin	Doxorubicin 51.8; Carboplatin 47.8; Paclitaxel 47.2; Docetaxel 43.2; Paclitaxel/Carboplatin 40.5	Doxorubicin 49.5; Carboplatin 48.7; Paclitaxel 44.5; Docetaxel 42.1; Paclitaxel/Carboplatin 36.6	SD
15	Paclitaxel/Carboplatin	Doxorubicin/Carboplatin 64.9; Paclitaxel/Carboplatin 50.8; Docetaxel/Carboplatin 45.8 Gemcitabine/Cisplatin43.6; Docetaxel40.6; Cisplatin 37.2; Topotecan 32.8; Paclitaxel 31.7; Paclitaxel/Carboplatin 31.3; Gemcitabine/Carboplatin 28.8; Doxorubicin 27.4; Gemcitabine 22.2; Carboplatin 15.4	Paclitaxel/Carboplatin 60.2; Docetaxel/Carboplatin 51.3; Doxorubicin/Carboplatin 50.9; Gemcitabine/Cisplatin 39.7; Docetaxel 30.2; Cisplatin 28.7; Topotecan 28.6; Paclitaxel 25.2; Paclitaxel/Carboplatin 22.0; Gemcitabine/Carboplatin 19.4; Doxorubicin 11.7; Gemcitabine <10; Carboplatin <10	SD
16	Paclitaxel/Carboplatin	Doxorubicin/Carboplatin 56.3; Docetaxel 47.8; Paclitaxel/Carboplatin 46.9; Topotecan 43.8; Paclitaxel 36.0; Docetaxel/Carboplatin 34.4; Gemcitabine/Cisplatin 28.9; Cisplatin 27.8; Doxorubicin 26.7; Gemcitabine/Carboplatin 15.6; Carboplatin12.2; Gemcitabine <10	Paclitaxel/Carboplatin 72.7; Doxorubicin 71.2; Cisplatin 66.1; Doxorubicin/Carboplatin 65.6; Paclitaxel 64.5; Carboplatin 63.4; Docetaxel 63.4; Gemcitabine 59.9; Docetaxel/Carboplatin 65.5; Topotecan 44.1	SD
17	Gemcitabine	Docetaxel/Carboplatin 48.1; Gemcitabine 42.8; Doxorubicin 41.9; Paclitaxel/Carboplatin 37.4; Paclitaxel 28.5; Gemcitabine/Cisplatin 23.5; Carboplatin 23.4; Docetaxel 20.3; Cisplatin 20.3; Doxorubicin/Carboplatin 18.2; Gemcitabine/Carboplatin 16.5; Topotecan <10	Gemcitabine 43.0; Paclitaxel 41.3; Cisplatin 40.9; Doxorubicin 36.8; Carboplatin 36.5; Doxorubicin/Carboplatin 31.3; Docetaxel/Carboplatin 29.2; Gemcitabine/Cisplatin 29.0; Paclitaxel/Carboplatin 28.3; Gemcitabine/Carboplatin 22.3; Docetaxel 14.0; Topotecan <10	SD
18	Gemcitabine	Gemcitabine 50.5; Gemcitabine/Cisplatin 37.9; Cisplatin 32.7; Gemcitabine/Carboplatin 27.8; Doxorubicin/Carboplatin 23.7; Doxorubicin 18.2; Docetaxel <10; Carboplatin <10; Paclitaxel/Carboplatin <10; Topotecan <10; Paclitaxel <10; Docetaxel/Carboplatin <10	Gemcitabine 41.3; Cisplatin 33.2; Docetaxel/Carboplatin 30.7; Paclitaxel/Carboplatin 30.6; Docetaxel 28.0; Gemcitabine/Cisplatin 21.0; Paclitaxel 17.3; Doxorubicin 10.5; Topotecan <10; Doxorubicin/Carboplatin <10; Gemcitabine/Carboplatin <10; Carboplatin <10	SD
19	Docetaxel/Carboplatin	Docetaxel/Carboplatin 71.8; Paclitaxel/Carboplatin 55.8; Gemcitabine 42.5; Paclitaxel 34.7; Doxorubicin/Carboplatin 34.0; Gemcitabine/Carboplatin 28.1; Cisplatin 29.2; Gemcitabine/Cisplatin 28.1; Carboplatin 22.0; Docetaxel <10; Doxorubicin <10; Topotecan <10	Docetaxel/Carboplatin 69.4; Paclitaxel/Carboplatin 51.8; Gemcitabine 40.2; Paclitaxel 36.7; Doxorubicin/Carboplatin 30.0; Gemcitabine/Carboplatin 29.6; Cisplatin 29.0; Gemcitabine/Cisplatin 24.1; Carboplatin 12.5; Docetaxel <10; Doxorubicin <10; Topotecan <10	SD
20	Paclitaxel/Carboplatin	Paclitaxel/Carboplatin 100; Cisplatin 61.1; Paclitaxel 57.2; Doxorubicin/Carboplatin 49.8; Carboplatin 47.6; Doxorubicin 47.2	Paclitaxel/Carboplatin 72.6; Cisplatin 52.4; Paclitaxel 44.5; Doxorubicin/Carboplatin 42.1; Carboplatin 36.6; Doxorubicin 30.1	SD
21	Doxorubicin/Carboplatin	Doxorubicin/Carboplatin 85.6; Gemcitabine/Cisplatin 83.6; Doxorubicin 79.8; Docetaxel/Carboplatin 72.0; Cisplatin 70.0; Gemcitabine/Carboplatin 61.0; Paclitaxel/Carboplatin 54.1; Paclitaxel 49.0; Docetaxel 38.2; Gemcitabine 36.7; Topotecan 12.2; Carboplatin 10.9	Docetaxel/Carboplatin 86.9; Doxorubicin/Carboplatin 82.1; Docetaxel 76.2; Paclitaxel/Carboplatin 75.7; Paclitaxel 57.7; Doxorubicin 56.9; Topotecan 53.4; Gemcitabine/Cisplatin 34.6; Cisplatin 32.2; Gemcitabine 17.7; Carboplatin <10; Gemcitabine/Carboplatin <10	PR
22	Paclitaxel	Paclitaxel 68.9; Paclitaxel/Carboplatin 64.4; Doxorubicin 55.4; Gemcitabine/Cisplatin 52.6; Docetaxel/Carboplatin 46.1; Doxorubicin/Carboplatin 43.9; Paclitaxel/Carboplatin 39.9; Cisplatin 38.3; Gemcitabine/Carboplatin 38.3; Paclitaxel 34.9; Docetaxel 29.1; Topotecan <10; Carboplatin <10; Gemcitabine <10	Paclitaxel/Carboplatin 78.8; Doxorubicin/Carboplatin 68.3; Doxorubicin 46.1; Gemcitabine/Cisplatin 45.0; Cisplatin 38.3; Docetaxel/Carboplatin 25.4; Paclitaxel/Carboplatin 23.6; Docetaxel Carboplatin 11.0; Paclitaxel <10; Gemcitabine/Carboplatin <10; Gemcitabine <10	PR
23	Doxorubicin	Doxorubicin 35.7; Docetaxel/Carboplatin 30.4; Paclitaxel 23.0; Doxorubicin/Carboplatin 22.6; Gemcitabine 22.2; Paclitaxel/Carboplatin 17.7; Carboplatin 13.4; Cisplatin 11.5; Docetaxel <10; Gemcitabine/Cisplatin <10; Gemcitabine/Carboplatin <10; Topotecan <10	Docetaxel/Carboplatin 54.7; Doxorubicin 52.5; Doxorubicin/Carboplatin 46.4; Gemcitabine 44.7; Doxorubicin 42.9; Gemcitabine/Cisplatin 41.7; Paclitaxel 41.6; Carboplatin 41.3; Paclitaxel/Carboplatin 38.9; Cisplatin 35.0; Docetaxel 20.1; Gemcitabine/Carboplatin 19.4; Topotecan <10	PR
24	Paclitaxel/Carboplatin	Doxorubicin/Carboplatin 62.5; Docetaxel 54.8; Paclitaxel/Carboplatin 51.8; Gemcitabine/Cisplatin 50.3; Topotecan 45.6; Cisplatin 44.1; Gemcitabine/Carboplatin 36.8; Docetaxel/Carboplatin 36.5; Paclitaxel/Carboplatin 34.6; Paclitaxel 29.9; Carboplatin 21.7; Gemcitabine 18.1; Doxorubicin 13.6	Doxorubicin 40.6; Paclitaxel/Carboplatin 40.3; Docetaxel/Carboplatin 39.7; Doxorubicin/Carboplatin 38.6; Paclitaxel/Carboplatin 38.6; Gemcitabine/Cisplatin 28.9; Docetaxel 28.5; Topotecan 22.0; Paclitaxel 20.6; Gemcitabine/Carboplatin 15.4	PR
25	Doxorubicin/Carboplatin	Doxorubicin/Carboplatin 79.9; Doxorubicin 75.4; Docetaxel/Carboplatin 68.7; Docetaxel 64.4; Gemcitabine/Cisplatin 61.0; Paclitaxel/Carboplatin 59.2; Topotecan 50.5; Cisplatin 50.5; Paclitaxel 31.1; Gemcitabine/Carboplatin 26.8; Carboplatin 11.5; Gemcitabine<10	Docetaxel/Carboplatin 84.9; Docetaxel 62.9; Doxorubicin/Carboplatin 38.5; Gemcitabine/Cisplatin 35.6; Cisplatin 31.2; Paclitaxel/Carboplatin 30.0; Doxorubicin <10; Paclitaxel <10; Gemcitabine/Carboplatin <10; Carboplatin <10; Topotecan <10; Gemcitabine <10	PR
26	Docetaxel/Carboplatin	Docetaxel/Carboplatin 40.5; Gemcitabine/Cisplatin 28.5; Cisplatin 25.6; Docetaxel 18.1; Doxorubicin 13.4; Paclitaxel 12.4; Carboplatin 11.6; Gemcitabine/Carboplatin <10; Gemcitabine <10; Topotecan <10	Docetaxel/Carboplatin 55.5; Gemcitabine/Cisplatin 22.5; Cisplatin 18.6; Docetaxel 18.0; Doxorubicin 11.9; Paclitaxel 10.4; Carboplatin 10.2; Gemcitabine/Carboplatin <10; Gemcitabine <10; Topotecan <10	PR
27	Doxorubicin/Carboplatin	Doxorubicin/Carboplatin 55.2; Paclitaxel/Carboplatin 52.6; Topotecan 37.8; Docetaxel 37.0; Docetaxel/Carboplatin 32.7; Paclitaxel 29.4; Cisplatin 23.8; Gemcitabine/Cisplatin 19.7; Doxorubicin 15.6; Gemcitabine/Carboplatin 14.4; Carboplatin 13.1; Gemcitabine <10	Doxorubicin/Carboplatin 73.8; Gemcitabine/Cisplatin 67.5; Cisplatin 64.0; Doxorubicin 55.7; Paclitaxel/Carboplatin 44.7; Paclitaxel 43.5; Carboplatin 37.1; Gemcitabine 34.9; Docetaxel/Carboplatin 31.2; Topotecan 22.4; Docetaxel 21.0	PR
28	Doxorubicin/Carboplatin	Doxorubicin/Carboplatin 49.1; Doxorubicin 44.0; Gemcitabine/Cisplatin 40.5; Gemcitabine 35.7; Cisplatin 26.9; Carboplatin 23.3; Topotecan 21.1; Paclitaxel/Carboplatin 15.6; Docetaxel/Carboplatin 14.7; Paclitaxel 11.6; Docetaxel <10; Gemcitabine/Carboplatin <10	Docetaxel/Carboplatin 37.5; Doxorubicin/Carboplatin 34.1; Gemcitabine/Cisplatin 33.9; Paclitaxel/Carboplatin 32.4; Paclitaxel 28.9; Doxorubicin 28.2; Carboplatin 25.8; Gemcitabine 21.5; Cisplatin 20.9; Docetaxel 18.8; Gemcitabine/Carboplatin 12.5; Topotecan <10	PR
29	Docetaxel	Paclitaxel/Carboplatin 76.6; Docetaxel 73.7; Docetaxel/Carboplatin 71.3; Paclitaxel/Carboplatin 51.4; Doxorubicin/Carboplatin 50.1; Topotecan 44.4; Paclitaxel 41.6; Cisplatin 27.9; Gemcitabine/Cisplatin 25.0; Gemcitabine/Carboplatin 17.6; Doxorubicin <10; Carboplatin <10; Gemcitabine <10	Docetaxel 51.2; Topotecan 45.5; Docetaxel/Carboplatin 46.2; Paclitaxel/Carboplatin 41.1; Doxorubicin/Carboplatin 37.2; Gemcitabine/Cisplatin 28.5; Paclitaxel 24.2; Carboplatin 14.5; Gemcitabine/Carboplatin <10; Doxorubicin <10; Gemcitabine <10	PR
30	Docetaxel/Carboplatin	Docetaxel/Carboplatin 83.5; Docetaxel 83.2; Doxorubicin/Carboplatin 75.1; Paclitaxel/Carboplatin 71.2; Gemcitabine/Cisplatin 46.2; Doxorubicin 43.0; Cisplatin 42.5; Topotecan 40.7; Gemcitabine/Carboplatin 27.1; Paclitaxel 26.1; Carboplatin <10; Gemcitabine <10	Docetaxel/Carboplatin 79.4; Paclitaxel/Carboplatin 65.2; Docetaxel 63.6; Doxorubicin/Carboplatin 59.9; Doxorubicin 51.2; Paclitaxel 50.2; Carboplatin 41.4	PR
31	Doxorubicin/Carboplatin	Doxorubicin/Carboplatin 69.6; Paclitaxel/Carboplatin 64.9; Docetaxel 60.7; Gemcitabine/Cisplatin 48.6; Cisplatin 47.2; Docetaxel/Carboplatin 44.1; Topotecan 40.6; Paclitaxel 37.6; Doxorubicin 36.1; Gemcitabine/Carboplatin 13.4; Carboplatin <10; Gemcitabine <10	Paclitaxel 79.9; Docetaxel 77.4; Doxorubicin 71.6; Cisplatin 67.4; Topotecan 66.3; Paclitaxel/Carboplatin 65.1; Gemcitabine/Cisplatin 64.5; Docetaxel/Carboplatin 63.9; Gemcitabine 62.8; Carboplatin 61.5; Doxorubicin/Carboplatin 59.7	PR
32	Docetaxel/Carboplatin	Docetaxel/Carboplatin 51.2; Carboplatin 42.7; Cisplatin 40.3; Paclitaxel/Carboplatin 38.5; Doxorubicin 37.1; Doxorubicin/Carboplatin 33.8; Gemcitabine/Carboplatin 28.7; Docetaxel 27.7; Gemcitabine/Cisplatin 27.3; Paclitaxel 27.1; Gemcitabine 24.7; Topotecan <10	Docetaxel/Carboplatin 50.2; Docetaxel 42.2; Paclitaxel/Carboplatin 40.1; Doxorubicin/Carboplatin 29.3; Topotecan 28.8; Cisplatin 26.5; Gemcitabine/Cisplatin 18.9; Paclitaxel 14.5; Doxorubicin <10; Gemcitabine/Carboplatin <10; Gemcitabine <10; Carboplatin <10	PR
33	Gemcitabine/Cisplatin	Docetaxel/Carboplatin 75.6; Gemcitabine/Cisplatin 68.8; Docetaxel 66.2; Cisplatin 63.2; Paclitaxel/Carboplatin 56.9; Doxorubicin/Carboplatin 52.6; Doxorubicin 44.8; Paclitaxel 25.9; Carboplatin 15.2; Gemcitabine 11.1	Gemcitabine/Cisplatin 61.3; Docetaxel/Carboplatin 52.3; Paclitaxel/Carboplatin 51.9; Docetaxel 48.7; Cisplatin 47.6; Doxorubicin/Carboplatin 42.7; Doxorubicin 41.3; Paclitaxel 35.2; Carboplatin 27.8; Gemcitabine 20.3	PR
34	Docetaxel/Carboplatin	Docetaxel/Carboplatin 58.3; Docetaxel 58.1; Paclitaxel/Carboplatin 53.6; Doxorubicin/Carboplatin 52.5; Topotecan 42.4; Cisplatin 38.3; Gemcitabine/Cisplatin 35.1; Paclitaxel 32.4; Doxorubicin 30.2; Gemcitabine/Carboplatin 29.0; Gemcitabine 25.5; Carboplatin 20.7	Docetaxel/Carboplatin 63.4; Docetaxel 45.3; Paclitaxel/Carboplatin 40.2; Doxorubicin/Carboplatin 22.6; Topotecan 23.8; Cisplatin 12.0; Gemcitabine/Cisplatin 10.9; Paclitaxel <10; Doxorubicin <10; Gemcitabine/Carboplatin <10; Gemcitabine <10; Carboplatin <10	PR
35	Doxorubicin/Carboplatin	Paclitaxel/Carboplatin 68.6; Doxorubicin/Carboplatin 63.1; Docetaxel/Carboplatin 60.5; Carboplatin 52.9; Gemcitabine/Cisplatin 52.7; Paclitaxel 46.9; Docetaxel 45.6; Topotecan 45.6; Doxorubicin 40.9; Gemcitabine/Carboplatin 40.6; Carboplatin 38.9; Gemcitabine 36.8; Cisplatin <10	Doxorubicin/Carboplatin 85.3; Paclitaxel/Carboplatin 83.3; Paclitaxel 70.4; Doxorubicin 53.5; Gemcitabine/Cisplatin 42.1; Cisplatin 35.4; Topotecan 34.5; Docetaxel/Carboplatin 30.1; Docetaxel 28.4; Carboplatin 16.4; Gemcitabine/Carboplatin 15.0; Gemcitabine <10	PR
36	Docetaxel/Carboplatin	Docetaxel/Carboplatin 76.2; Docetaxel 72.2; Doxorubicin/Carboplatin 40.0; Topotecan 32.5; Paclitaxel/Carboplatin 31.9; Gemcitabine/Cisplatin 28.1; Cisplatin 22.4; Doxorubicin 21.2; Carboplatin 18.9; Paclitaxel 17.6; Gemcitabine/Carboplatin 14.1; Gemcitabine 11.4	Docetaxel/Carboplatin 80.5; Docetaxel 76.7; Doxorubicin/Carboplatin 55.7; Paclitaxel 48.9; Doxorubicin 48.2; Paclitaxel/Carboplatin 45.9; Carboplatin 29.7; Topotecan<10	PR
37	Doxorubicin/Carboplatin	Doxorubicin/Carboplatin 62.3; Paclitaxel/Carboplatin 53.8; Doxorubicin 50.6; Docetaxel 33.6; Paclitaxel 33.6; Gemcitabine/Cisplatin 31.2; Topotecan 30.1; Docetaxel/Carboplatin 29.6; Cisplatin 26.1; Gemcitabine/Carboplatin 14.3; Carboplatin 13.2; Gemcitabine 12.7	Docetaxel/Carboplatin 70.5; Docetaxel 66.5; Doxorubicin/Carboplatin 63.5; Paclitaxel/Carboplatin 61.8; Paclitaxel 52.5; Topotecan 39.8; Gemcitabine/Cisplatin 30.5; Cisplatin 25.9; Doxorubicin 19.1; Carboplatin 11.5; Gemcitabine/Carboplatin <10; Gemcitabine <10	PR
38	Doxorubicin/Carboplatin	Doxorubicin/Carboplatin 86.3; Doxorubicin 81.1; Docetaxel/Carboplatin 65.8; Paclitaxel/Carboplatin 56.6; Gemcitabine 51.2; Docetaxel 43.3; Paclitaxel 40.0; Gemcitabine/Carboplatin 32.8; Carboplatin 31.1; Cisplatin 27.8; Topotecan <10; Gemcitabine/Cisplatin <10	Doxorubicin/Carboplatin 61.3; Doxorubicin 52.3; Docetaxel/Carboplatin 48.7; Paclitaxel/Carboplatin 47.6; Gemcitabine 42.7; Docetaxel 41.3; Paclitaxel 35.2; Gemcitabine/Carboplatin 30.0; Carboplatin 26.4; Cisplatin 20.3; Topotecan <10; Gemcitabine/Cisplatin <10	PR
39	Paclitaxel/Carboplatin	Paclitaxel/Carboplatin 83.0; Gemcitabine/Cisplatin 43.3; Doxorubicin/Carboplatin 39.2; Docetaxel/Carboplatin 37.0; Paclitaxel/Carboplatin 31.1; Topotecan 29.5; Cisplatin 26.1; Docetaxel <10; Paclitaxel <10; Gemcitabine <10; Carboplatin <10; Doxorubicin <10	Paclitaxel/Carboplatin 76.3; Gemcitabine/Cisplatin 38.9; Doxorubicin/Carboplatin 37.1; Docetaxel/Carboplatin 35.4; Paclitaxel/Carboplatin 38.1; Topotecan 32.0; Cisplatin 31.3; Docetaxel 23.0; Paclitaxel 22.2; Gemcitabine 11.4; Carboplatin <10; Doxorubicin <10	CR
40	Doxorubicin/Carboplatin	Doxorubicin/Carboplatin 95.2; Doxorubicin 88.5; Gemcitabine/Cisplatin 80.9; Docetaxel/Carboplatin 78.7; Paclitaxel/Carboplatin 78.5; Docetaxel 74.2; Cisplatin 71.9; Topotecan 61.0; Paclitaxel 59.9; Gemcitabine/Carboplatin 56.2; Carboplatin 30.4; Gemcitabine 25.7	Docetaxel/Carboplatin 95.3; Docetaxel 88.3; Doxorubicin/Carboplatin 87.3; Paclitaxel/Carboplatin 87.1; Doxorubicin 69.2; Gemcitabine/Cisplatin 62.7; Topotecan 58.2; Cisplatin 55.5; Paclitaxel 51.0; Gemcitabine/Carboplatin 30.6; Gemcitabine 15.7; Carboplatin <10	CR

The percentage of change in tumor burden calculated by RECIST 1.1 criteria of subjects in the Physician-Choice group vs those in the ChemoID-Guided group is shown in Figs. 11B and 12B. Tumor assessments were performed by 2 independent radiologists who agreed 95% of the time on lesion measurements of the CT images and a third senior reader was used to adjudicate 4 disagreements. The majority of subjects in the Physician-Choice group had progressive disease (PD: 32/41, 78%), 8 subjects had stable disease (SD: 8/41, 19.5%), and 2 subjects had a response to treatment (1 PR and 1 CR: 2/41, 2.5%). Conversely, most of the subjects in the ChemoID-Guided group had a response to treatment (18 PR and 2 CR: 20/40, 50%), 13 subjects had stable disease (SD: 13/40, 32.5%), and 7 subjects had progressive disease (PD: 7/40, 17.5%).

Tables 6 and 7 illustrate the complete dataset of all the predicted drugs by the ChemoID assay in the entire cohort of patients (from both arms), the regimen used to treat each subject, and the outcome of treatment(s) administered. Each participant is labeled with a unique progressive number corresponding to the number indicated in Figs. 11 and 12.

## Discussion

Ovarian cancer has a reported response rate of 75–80% with frontline therapy^33^. However, 70% of tumors will recur and eventually become platinum-resistant, which has been defined as disease relapse within 6 months after the last dose of a platinum-based therapy^34,35^, which is associated with significant morbidity and mortality^1^. The choice of which agent to use in the recurrent disease setting is ordinarily based on the toxicity profile of the drug(s), the previous toxicities experienced by the patient, comorbidities, molecular signature, number of prior lines, and patient/physician preference^36^.

Currently, the standard of care for PROC is sequential single-agent non platinum chemotherapy or enrollment in a clinical trial^37^. Nonplatinum chemotherapy for PROC has been associated with low objective response rates (ORRs, <12%), short progression-free survival (PFS, <4 mo) and OS ( <12 mo)^38–41^, and significant adverse effects, which can impair quality of life^38,42^. Several preclinical studies and clinical trials support the notion that patients with platinum-resistant ovarian cancer can regain sensitivity to platinum-based therapies after a platinum-free interval^15,37^. High response, disease control rates, and long-term OS have been observed following platinum rechallenge therapy for patients with platinum-resistant ovarian cancer recurrence, bringing forward the idea that platinum rechallenge therapy for platinum-resistant ovarian cancer may be a viable treatment option^6–19^. Additionally, these studies suggested that their findings further question the use of a 6-month PFI as an arbitrary threshold for subsequent treatment decision-making because several patients considered “platinum-resistant” still derive clinical benefit from platinum-based chemotherapy^6^. For all these reasons, our trial included platinum-based regimens in the panel of drugs tested by the ChemoID assay.

The aggressiveness of recurrent EOC is mostly attributed to the presence of ovarian cancer stem cells (CSCs), which are chemo-resistant and responsible for the recurrence of cancer^43–46^. Individual patient responses to standard-of-care treatments greatly vary and, unfortunately toxicity profiles are extensive for most chemotherapy drugs. For these reasons, there is an unmet need for ways to tailor chemotherapy regimens based on patients’ tumor response profiles to identify treatments that may improve clinical outcomes.

This randomized pivotal study demonstrates the utility of the ChemoID cancer stem cell chemotherapeutics assay for the management of poor prognosis recurrent PROC patients. ChemoID is a functional precision medicine assay that uses an individual patient’s live bulk of tumor cells and CSCs isolated from tumor biopsies or malignant fluid aspirates (peritoneal and/or pleural fluid) to indicate which chemotherapy regimens are most effective. Targeting CSCs alongside the bulk of other cancer cells is a new paradigm in cancer treatment, that has already demonstrated clinical validity in a recent randomized clinical trial by improving mOS of subjects affected by recurrent GBM who were treated with assay-guided therapy vs. standard-of-care from the same panel of first-, second- and third-line conventional cytotoxic chemotherapies^29^. These findings reinforce the importance of using the ChemoID assay, which is an actionable tool for physicians that allows for personalized cancer treatment by selecting the most effective therapies against CSCs from a panel of approved cytotoxic agents.

Recent trials in platinum-resistant ovarian cancer have mostly yielded negative outcomes, with none having a clinically significant effect on progression-free or overall survival since the approval of bevacizumab in combination with chemotherapy^37^.

In the past 5 years MIRASOL trial stands out as the only study demonstrating a significantly improved response rate (ORR of 42%) for mirvetuximab, a targeted therapy against folate receptor alpha (FRα), in platinum-resistant ovarian cancer patients with high-grade serous histology who have received 1–3 prior lines of treatment, when compared to standard investigator-chosen chemotherapy^41^.

The NCT03949283 is the first-in-human randomized trial to demonstrate that prospective interventional use of a functional precision medicine test such as the ChemoID assay to select effective treatments for poor prognosis patients with recurrent PROC resulted in a significantly improved response rate. The ORR of the subjects in the control group of our trial was 5% (CI_95_ 0–11%) and their DOR was 5.5 months, which is consistent with previously published data^47^. The ORR of subjects treated with chemotherapies guided by the ChemoID assay was instead 50% (CI_95_ 35–65%) with an associated *p*-value <0.0001, and their respective DOR was 8.0 months with an associated *p*-value <0.0001.

The novelty of the ChemoID assay is its focus on CSCs, which are implicated in resistance to platinum-based therapies. Previous publications describing older chemosensitivity tests attempted earlier were retrospective studies that only included the bulk of the tumor testing. In this era of personalized medicine, previous prospective and/or retrospective investigations have shown that the ORR for women with platinum-resistant disease ranges from 5% to 30%, and the duration of their response is typically less than 6 months to chemotherapeutic agents such as pegylated liposomal doxorubicin (PLD), topotecan, taxanes, etoposide, and gemcitabine^20,39^.

In our study, we found an objective response rate of 5% (CI_95_ 0–11%) in the comparator arm, which is on the lower end of reported response rates for chemotherapy in platinum-resistant ovarian cancer. While response rates for standard therapies in this setting typically range from 10 to 20%, it is important to consider the specific patient population included in our study. The NCT03949283 trial allowed for up to five prior lines of therapy, which is more than some of the studies referenced. Given that heavily pretreated patients generally have more resistant disease and lower response rates, this may explain the observed response in the comparator arm. Additionally, this response rate may better reflect real-world outcomes in patients who have undergone multiple prior lines of treatment, a population often underrepresented in clinical trials. We recognize that some clinicians may consider a 5% response rate lower than expected for a comparator arm in platinum-resistant ovarian cancer studies. However, given the inclusion of patients with extensive prior treatment histories, the observed response rate may more accurately reflect outcomes in heavily pretreated patients seen in routine clinical practice. This highlights the challenge of treating patients with advanced platinum-resistant disease and underscores the need for more effective therapeutic strategies in this setting.

The ChemoID assay identified high-suppression drugs against CSCs and the bulk of tumor cells contributing to a higher rate of response and a more durable clinical response in a statistically significant manner. This study revealed that patients who were treated with a chemotherapy-sensitive regimen against CSCs had an improvement in the rate of response determined by RECIST criteria and the duration of their response compared to patients who were treated with an empirical choice of regimens from the same list of drug(s). This method of determining the responses of CSCs to available FDA-approved chemotherapies for the treatment of ovarian cancer provided critical information about an individual patient’s likelihood of achieving a response that is more durable before implementing the patient’s treatment plan.

Moreover, the ChemoID assay improved in a statistically significant manner (*p* <0.0001) from 24% to 83% the clinical benefit rate, calculated by including the response of patients who had PR and CR those who had SD, indicating the important impact on the quality of life of these patients affected by platinum-resistant recurrent ovarian cancer. The improved clinical benefit is even more evident in Figs. 11B and 12B, where the response to chemotherapy in each group was ordinated by the change in tumor burden that was observed post-treatment, demonstrating that the majority of patients treated with chemotherapies without the aid of the ChemoID assay had an increase in their tumor burden, while the majority of patients treated with chemotherapies predicted by the ChemoID assay had a sensible decrease of their tumor burden.

CA125 has been used as a biomarker for ovarian cancer, which is elevated in the serum of more than 80% of patients with ovarian cancer. Several studies have shown its utility in monitoring if treatments are effective and/or for screening for cancer recurrence. A phase III study showed that radiological response by RECIST was preceded by a favorable predictive CA125 decrease in a high proportion of patients, suggesting that CA125 evaluation may be an appropriate tool for tumor assessment in patients with ovarian cancer^48^. Additionally, early changes in CA125 levels following primary chemotherapy treatments for EOC predict improvement in platinum sensitivity, PFS, and OS^49^, indicating that serum CA125 levels after the first cycle of chemotherapy and time to normalization were significant prognostic factors for both OS and PFS^48–51^.

Even though in our study the association between treatment groups and tumor response was not modified by CA125, in agreement with previous reports^48–51^ we observed a more rapid change in CA125 levels (5-fold difference between the slopes) between the screening visit (baseline) and the first follow-up visit post-treatment in patients treated with ChemoID-guided regimens who had better response than patients in the physician-choice group.

The results of this trial also suggest that the ChemoID assay provides more treatment options, with an overall response rate of 50% and a PFS of 11 months using ordinary cytotoxic chemotherapies for improved outcomes compared to the current 5–10% response rate and PFS of 3–4 months achieved by standard of care treatment in recurrent ovarian cancer. This is particularly beneficial and important in light of the new value-based healthcare models, where payment is contingent on the performance of outcomes-based contracts for the indication of certain anticancer medication costs, raising questions regarding the affordability and accessibility of treating recurring EOC patients. Treatments with more expensive targeted anti-cancer drugs and immunotherapies are not always practical due to socioeconomic and health disparity issues in the US and globally. Our trial focused on screening SOC chemotherapies that are routinely covered by insurance and used by community oncologists globally, thus highlighting the clinical effectiveness of a personalized approach to treatment which is a promising strategy to provide more affordable treatment for patients with recurrent PROC. However, because the ChemoID assay is adaptable, a more expanded panel of drugs is being currently explored to increase the number of drugs tested to offer patients more flexibility to include other new agents in the future. We foresee personalized anti-cancer therapy targeting CSCs will be included sooner in the treatment plan, thereby eliminating ineffective treatments and allowing patients to gain the greatest therapeutic benefit possible.

Although this study provides promising treatment options for recurrent PROC patients, some potential limitations should be noted. For example, ChemoID is a functional assay limited by the availability of viable tumor samples. Our study only included recurrent PROC subjects where malignant cells could be safely obtained via tissue biopsy or paracentesis. The specimen collection quality is paramount for the success of the assay, which could be a limiting factor. However, this can be successfully resolved by training the sample collection team.

The median turnaround time from biopsy to assay results was 14 days (range 5–15 days from the time of biopsy to the time of results). As a consequence, patients receiving tamoxifen as a bridging therapy were on treatment for 14 days in a median while awaiting test results. Although this duration is unlikely to have significantly influenced overall outcomes, we acknowledge that any delay in initiating an optimal therapy could be a concern in certain cases and we are streamlining the assay process to minimize any delays in future applications.

Currently, the assay is performed in a central laboratory due to the specialized equipment and expertize required for its execution. While this limits immediate widespread availability in standard clinical laboratories, ongoing efforts aim to optimize the assay for broader implementation in the future.

Regarding costs, although the ChemoID assay incurs an additional expense, its clinical utility is found in guiding treatment selection to avoid ineffective chemotherapy, thereby potentially lowering overall healthcare costs linked to unsuccessful treatments and unnecessary toxicity. Given that the cost associated with the ChemoID assay is significantly less compared to the cost of ineffective chemotherapy treatments, the results of this study suggest that the use of the assay-guided treatments may offset financial toxicity for these patients. As noted earlier, this cost-effectiveness is especially significant for patients with platinum-resistant disease, where treatment selection remains a major challenge. Nevertheless, we recognize that any additional diagnostic expense must be considered in the context of the cost of standard clinical care.

## Methods

### Study design and participants

The ChemoID study was a multicenter blind randomized clinical trial designed to assess whether functional precision medicine ChemoID assay-guided selection of chemotherapy improved the objective response rate of recurrent platinum-resistant epithelial ovarian cancer patients vs best physician-choice chemotherapy regimen selection (NCT03949283). Patients were blinded to randomization group assignment. Physicians and investigators were not provided with ChemoID test results for patients randomized to the physician-choice control arm and unblinding of test results was not permitted.

Physicians and investigators were provided with a report of the ChemoID assay only for patients randomized to the ChemoID-guided arm.

The study protocol was approved by the Central Western Institutional Review Board (WIRB) under study protocol ID: 20191094 for the University of Cincinnati Cancer Center, Allegheny Health Network Cancer Institute, Miami Cancer Institute, Louisiana State University, Edwards Cancer Center, and Stephenson Cancer Center University of Oklahoma Health Sciences Center. The study protocol was also approved by the Institutional Review Board of Kaiser Permanente Los Angeles Medical Center under study protocol ID: 12411, and the Charleston Area Medical Center under study protocol ID: 19-643. All patients signed the informed consent before enrollment in the study. The clinical trial protocol is available in Supplement 1. The IRB protocol was approved on April 11, 2019, and the trial was registered on ClinicalTrials.gov with the Identifier NCT03949283 on May 11, 2019. Patients were enrolled in the trial between January 31, 2020, and April 15, 2023, following the Declaration of Helsinki^52^ and the International Conference on Harmonization Good Clinical Practice guidelines. Data analysis was performed at the end of December 2023.

136 patients were screened and 81 subjects meeting the inclusion and exclusion criteria for study participation (Table 1) with recurrent PROC were enrolled over three years in the randomized controlled clinical trial (Consort Diagram – Fig. 1). The population with PROC patients (PFI1 <6 months) included members of all ethnic groups, at least 18 years old, who received ≤ 5 prior regimens (including at least 1 platinum-based regimen) for their epithelial ovarian carcinoma. In all cases, the diagnosis was confirmed by a pathologist according to the WHO classification of ovarian tumors. Participants in the trial had measurable disease by imaging or objective physical parameters. Participants with CA-125 only disease without RECIST 1.1 measurable or otherwise evaluable disease were excluded from the trial.

All enrolled subjects before randomization underwent surgical biopsy or fluid aspiration of ascites as per standard of care. Fresh tissue biopsy samples were collected under sterile conditions and divided into two portions. One portion of the biopsy was sent to the central ChemoID laboratory by an overnight courier in a clinical pack containing a sterile vial with RPMI transportation medium at room temperature. Upon arrival, patients’ identifiers were recorded, and the tissue was triaged for the growth of bacteria and yeast/fungi and accepted at the ChemoID laboratory. The assay used in this study to guide treatment was performed by an independent hospital pathology laboratory regulated by the Centers for Medicare & Medicaid Services (CMS), which oversees all laboratory testing performed on humans in the U.S. through the Clinical Laboratory Improvement Amendments (CLIA) guidelines. The second portion of the biopsy was placed in a 10% formaldehyde solution and sent to the local pathology lab for histopathological confirmation to satisfy the main inclusion criterion. Post-surgery/biopsy, patients received a baseline CT scan of the thorax, and of the abdomen and pelvis. The ChemoID assay was performed on all enrolled subjects so that retrospective analysis could be conducted on patients randomly assigned to the physician-choice group. Patients were randomly assigned 1:1 using block randomization by the sites’ coordinators to one of the two study groups using an automatic computer-generated algorithm (in REDCap). The patients were treated either with physician-selected standard-of-care chemotherapy versus treatment directed by the ChemoID assay, depending on the randomly allocated study group.

### Generation of primary cancer cell lines from tumor biopsies

To generate the primary tumor cell cultures, the fresh tumor tissue from surgical biopsies was minced using sterile scalpel blades and gently dissociated in a biosafety cabinet using 0.025% trypsin solution at 37 °C for 10 min with gentle agitation and intermittent resuspension. Dissociated tumor cells were plated in RPMI with 20% FBS, 1% Penicillin/Streptomycin, and Gentamycin Sulfate (complete media) in sterile plastic Petri dishes in the presence of residual tumor tissues and incubated at 37°C humid tissue culture incubator in the presence of 5% CO_2_. Cancer cells in ascites were spun down and cultured in RPMI with 20% FBS, 1% Penicillin/Streptomycin, and Gentamycin Sulfate (complete media) in sterile plastic Petri dishes and incubated at 37 °C humid tissue culture incubator in the presence of 5% CO_2_. Primary cancer cells were passaged to confluency and subcultured in complete media in additional sterile plastic Petri dishes.

### Enrichment of cancer stem cells (CSCs)

**Fig. 13:**
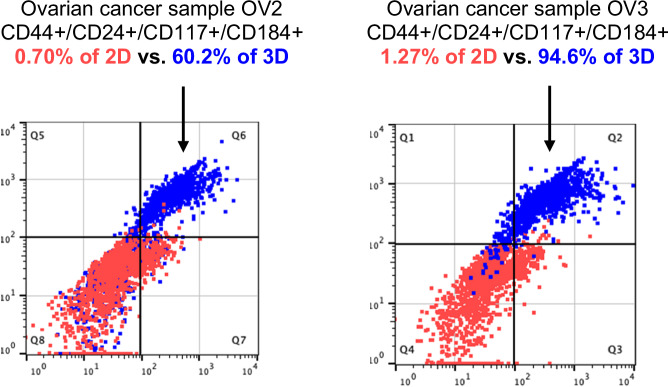
The bulk of tumor cells (grown in 2D) and the CSCs (grown in 3D) were labeled with fluorescent-conjugated antibodies against CD24, CD44, CD117, and CD184. A fluorescence shift can be observed in the expression of CD44/CD24/CD117/CD184 in the CSCs enriched using the 3D bioreactor (in blue) vs the bulk of tumor cells grown in 2D (in red). Sample OV2 contains 0.7% of the bulk of tumor cells (2D) expressing CD44/CD24/CD117/CD184 vs 60.2% of the CSCs (3D) enriched in the bioreactor. Sample OV3 contains 1.27% of the bulk of tumor cells (2D) expressing CD44/CD24/CD117/CD184 vs 94.6% of the CSCs (3D) enriched in the bioreactor.

Patient-derived CSC cultures were obtained as previously described^28–31,53,54^. The CSCs were enriched from the primary tumor cell cultures by loading a 3D cell culture rotating bioreactor (Cordgenics) with a volume of 50 mL and a gas-permeable membrane that allows for gas exchange where cells will aggregate in suspension to form cell aggregates in the absence of shear forces. The 3D-suspension cell culture rotating bioreactor can control the movement of air bubbles and remove them from the bioreactor without degrading the low-shear culture environment or the suspended three-dimensional tissue assemblies. This provides unparalleled control over the locations of cells and tissues within its bioreactor vessel during operation and sampling. Both the low-shear suspension of cells and control of the locations of cells and air bubbles are affected using the hydrodynamic force created by the flow within the vessel and fluid drag along the surface of the viscous spinner. A gas-permeable membrane connected to the base of the vessel enables the exchange of gas between the tissue culture medium in the vessel and an incubator environment in which the vessel is placed. A conic spinner on the axis of rotation of the cell culture rotating bioreactor enables the simultaneous creation of a low-shear culture environment and the “herding” of suspended cells and tissue assemblies, which is responsible for the CSCs’ selective growth. A rotation rate of 15–25 rpm was estimated to have average sheer values of 0.001 dyn per square centimeter, which is the rate at which medium-large, three-dimensional, tissue-like suspended growth assemblies have been successful. The rotating bioreactor was maintained in an incubator with constant CO_2_, temperature, 20% airflow, and at 20–25 rpm rotation speed. CSCs from primary cancer cells (bulk of the tumor cells) were enriched by loading 2 × 10^6^ bulk of tumor cells into the bioreactor and culturing them for 7 days in RPMI media in the absence of growth factors. The bulk of tumor cells (grown in 2D) and the CSCs (grown in 3D) were labeled with fluorescent-conjugated antibodies against CD24, CD44, CD117, and CD184 (BD Biosciences). Figure 13 shows the flow cytometric analysis of CD44, CD24, CD117, and CD184 expression in patient-derived primary ovarian cancer cell lines (Bulk of Tumor - Baseline) and bioreactor-enriched CSCs. CD44, CD24, CD117, and CD184 expression identify ovarian CSCs^46,55–57^.

### Intervention

The cytotoxic chemotherapy regimens used in the trial were chosen by all participating investigators and were covered by insurance, so study participants had no additional costs. All trial participants, independently of arm randomization, were given Tamoxifen as a maintenance drug while waiting for the assay results until the day before chemotherapy treatment began. The median turnaround time from biopsy to assay results was 14 days, with a range of 5–15 days from the time of biopsy to the time of results. As a consequence, patients receiving maintenance tamoxifen therapy were on treatment for 14 days in a median while awaiting test results.

The regimens and doses of chemotherapy used to treat subjects in the two trial groups were identical (Fig. 2). Patients received 1 of the 13 cytotoxic chemotherapy regimens either chosen by the physician or guided by the ChemoID assay test report.

The ChemoID assay-guided group received the regimen selected from the high-cell kill drugs on CSCs based on the ChemoID assay report. The treatment given to subjects in the control group was selected from the same list of chemotherapies tested by the assay, based on the treating physician’s best empirical judgment. All participants continued treatment until there was a documented progression, unacceptable toxicity, or consent withdrawal.

### Assessment of response to chemotherapy of CSCs and the bulk of tumor cells

Treatments with anti-cancer drugs and sensitivity tests were performed as previously described^28–31,53,54^. The bulk of tumor cells and CSCs were counted using trypan blue exclusion to determine cell viability and cell number before chemosensitivity testing using a Cellometer mini automated cell counter.

96-well plates are seeded in RPMI-1640 with 10% FBS, penicillin and streptomycin with a minimum of 20,000 individual tumor cells per regimen of bulk tumor cells or CSCs in 5 replicas and incubated at 37°C in a 5% CO_2_ incubator. After 24 h from plating, clinical-grade chemotherapy drugs were added alone or in combination for 1-h exposure at concentrations that do not exceed the serum C [max] described in pharmacokinetic (PK) studies, including the clinical dose. Three concentrations of each chemotherapy treatment were prepared by serial dilution. Each concentration was added to five replicate wells on the microtiter plate. Additionally, three replicated wells (control 1 = no treatment) and three replicated wells (control 2 = equal amount of solvent) were associated with each treatment.

After the 1-h exposure, the treatment media containing the various chemotherapies were removed and replaced with fresh media. MTT assay was performed 24 h following chemotherapy treatment to assess cell survival as previously described^28–31,53,54^.

Inhibition of bulk tumor cells and CSCs survival was measured for each concentration (average counts in five replicates ±SE) of a given treatment (for a total of 14–18 different treatments per patient). Survival of tumor cells at each concentration was calculated as compared to control-2 and the overall percent of the bulk of tumor cells and CSCs killed was calculated for each treatment as the primary measures of potential therapy efficacy.

### Reporting of the ChemoID assay results

Percent survival (potential therapeutic efficacy) was calculated relative to appropriate negative and positive controls for each treatment. The median turnaround time for generating the ChemoID assay results for trial participants was 14 days, with a range of 5–15 days from the time of biopsy to the time of results. Efficacy and resistance of each drug and combinations were reported on the ChemoID assay results as a continuous number from <10% to 100% cell-kill as previously^28–31,53,54^.

### Assessment of tumor response

As per clinical protocol, participants were followed according to standard-of-care intervals by clinical assessments or until disease progression. Because of the differences in chemotherapy cycle lengths between the allowed regimens, tumor reassessment was time-based, and not cycle-based, with a CT scan performed as per standard of care once every 8 weeks (± 7 days) for the first year and every 12 weeks (± 7 days) after the first year, and at any other time if clinically indicated. Tumor response to chemotherapy was evaluated according to the Response Evaluation Criteria in Solid Tumours (RECIST 1.1) criteria^58^ at the 6-month follow-up visit. Tumor assessments were performed by an independent radiology service composed of 2 readers and a third senior reader for adjudication of disagreements. All radiologists were blinded to groups and/or treatment assignments throughout the trial to determine the earliest time of progression independent of the impressions of the treating physicians to avoid bias.

### Quantification and Statistical Analysis

All statistical analyses followed the plan specified in the protocol with no deviations and were completed using Stata v17.1 (StataCorp) by independent biostatisticians. Data were analyzed from January 31, 2020 (first patient) to the end of December 2023 (first interim analysis), when 75 patients had completed the required follow-ups in the trial. A data manager and an independent statistical services conducted data monitoring and analysis of results.

For our primary analyses comparing objective response rate (ORR), with *N* = 220, a 1:1 ratio between treatment groups, an overall alpha rate of 0.05, and beta rate of 0.2 and interim analysis described below, we will have over 80% power to detect an improvement in ORR from 10% to 25%, giving a odds ratio of 0.33 at the final analysis.

Three prespecified interim analyses were planned a priori at 75, 100, and 150 at a total sample size of 220. The stopping guidelines for either benefit or futility were based on Obrien-Fleming spending functions. According to the protocol, at our first interim analysis (*N* = 75) to conclude the trial and declare either a success or no success we needed a detectable odds ratio less than or equal to 0.33 (we observed OR = 0.044) with a *p*-value less than 0.00005 (we observed *p* = 0.0000006) for our primary outcome of ORR. Likewise, at sample sizes of 100 and 150, the corresponding stopping *p*-values would need to be 0.0039 and 0.0184, respectively. At full collection (*N* = 220), due to the interim analyses and to preserve the experiment-wise error rates, analyses would have to achieve *p*-values less than 0.0412 to be considered statistically significant.

As per protocol, initial analyses involved data cleaning, variable development, and exploratory data analyses. We used standard summaries to describe baseline characteristic distributions in terms of centrality, spread, shape, and possible outliers by arm, cohort, and treatment group. Graphical explorations emphasized the examination of the nature and extent of potential nonlinear relationships on the appropriate modeling scale (e.g. natural, log, logit, etc.).

Baseline characteristics were compared using t-tests or Mann–Whitney U tests, as appropriate, for continuous and ordinal variables and Fisher’s exact tests for categorical variables. Kaplan–Meier curves were constructed using established methods, and the median PFS was calculated from these curves.

The primary analysis included all subjects randomized at baseline under intention-to-treat principles. The primary efficacy outcome was objective response rate (ORR), defined as the number of patients with Complete Response + Partial Response at 6 months follow-up. This outcome was compared between patients randomized to ChemoID-guided chemotherapy versus chemotherapy chosen by the Physicians. ORR comparisons were based upon logistic regression models with a sole main effect for the study arm.

Secondary analyses included the following: Clinical benefit rate (CBR) defined as the number of patients with Complete Response + Partial Response + Stable Disease at 6 months follow-up, was modeled similarly to ORR with logistic regression. Progression-free survival was calculated between the two arms with Kaplan–Meier methods, while Cox proportional hazard models were used to obtain hazard ratios. Repeated measure outcomes of CA125 levels and Health-Related Quality of Life (HRQoL) outcomes were modeled using generalized linear mixed models with Huber-White robust standard errors and exchangeable correlation structures.

Recommended cut points for CSC kill and the bulk of tumor kill percentages were calculated with Youden indices. All statistical assumptions were verified. Graphical methods were used extensively throughout.

## Supplementary information

Supplement 1

## Data Availability

The study protocol and statistical analysis plan are available from the corresponding author upon request. The data that support the findings of this study are not openly available due to patient privacy, ethical, and legal issues. The de-identified participants’ data that underlie the results reported in this article, will be made available upon reasonable request to investigators whose proposals for the use of the data have been approved by an independent review committee. Proposals may be submitted to the corresponding author beginning 12 months up to 18 months from the publication date.

## Abbreviations

PROC: platinum-resistant ovarian cancer
EOC: epithelial ovarian cancer
ORR: objective response rate
CBR: clinical benefit rate
DOR: duration of response
PFS: Progression-Free-Survival
CSCs: cancer stem cells.

## Code availability

No code was generated/used in the preparation of this paper.
