# Supplementary material for: ChemoID-guided therapy improves objective response rate in recurrent platinum-resistant ovarian cancer randomized clinical trial
Source: NPJ Precis Oncol. 2025 Mar 25;9:86. doi: 10.1038/s41698-025-00874-0 (PMC11937309; doi:10.1038/s41698-025-00874-0)
Supplement: Supplementary file 1 — Supplement 1 [file 41698_2025_874_MOESM1_ESM.pdf]

Title:	**Standard Chemotherapy versus Cancer Stem Cell Assay Directed Chemotherapy in Recurrent Platinum Resistant Ovarian Cancer**
Type of Clinical Trial	Randomized Clinical Trial
Drug or Device Name(s):	ChemoID
FDA IND or IDE	N/A
Sponsor:	Cordgenics
Protocol Number:	CG03-EOC<br>WIRB® Protocol # 20191094
Protocol Approval Date:	April 11, 2019

Sponsor (ChemoID)

Address ChemoID Lab
Marshall University Medical Center/TGRI
1340 Hal Greer Boulevard
Huntington, WV, 25701

Study PI: Thomas Herzog, MD, University of Cincinnati

HERZOGTJ@ucmail.uc.edu

Study Co-PI: Camille Gunderson, MD, University of Oklahoma Health Sciences Center

Camille-Gunderson@ouhsc.edu

TABLE OF CONTENTS

TABLE OF CONTENTS II

1 ABBREVIATIONS AND DEFINITIONS OF TERMS V

2 ABSTRACT VIII

3 PROTOCOL SYNOPSIS XI

TABLE 3.1: SCHEDULE OF STUDY PROCEDURES XX

TABLE 3.2: STUDY DIAGRAM XXII

4 BACKGROUND INFORMATION AND RATIONALE 1
4.1 INTRODUCTION 1
4.2 NAME AND DESCRIPTION OF INVESTIGATIONAL PRODUCT OR INTERVENTION 3
4.3 FINDINGS FROM PREVIOUS CLINICAL STUDIES 6
4.4 CHEMOID SELECTION OF DRUGS AND DOSAGES 12
4.5 OTHER RELEVANT LITERATURE AND DATA 13
4.6 COMPLIANCE STATEMENT 14

5 STUDY OBJECTIVES 15
5.1 PRIMARY OBJECTIVES (OR AIM) 15
5.2 SECONDARY OBJECTIVE (OR AIM) 15

6 INVESTIGATIONAL PLAN 16
6.1 GENERAL SCHEMA OF STUDY DESIGN 16
6.1.1 Screening Phase 16
6.1.2 Study Treatment Phase 17
6.1.3 Follow-up Phase 17
6.2 ALLOCATION TO TREATMENT GROUPS AND BLINDING 18
6.3 STUDY DURATION, ENROLLMENT AND NUMBER OF SITES 18
6.3.1 Duration of Study Participation 18
6.3.2 Total Number of Study Sites/Total Number of Subjects Projected 18
6.4 STUDY POPULATION 18
6.4.1 Inclusion Criteria 18
6.4.2 Exclusion Criteria 19
6.5 REQUIREMENTS FOR STUDY ENTRY, TREATMENT, AND FOLLOW-UP 21
6.5.1 Pre-Treatment Assessment 21
6.5.2 Assessment During Treatment 22

7 TREATMENT PLAN AND ENTRY/RANDOMIZATION PROCEDURE 24
7.1 TREATMENT PLAN 24
7.2 DURATION OF THERAPY 24
7.3 TREATMENT WINDOW 25
7.4 ARM 1- PHYSICIANS CHOICE OF CHEMOTHERAPY REGIMENS 25
7.5 ARM 2 – CHEMOID DRUG RESPONSE ASSAY CHEMOTHERAPY REGIMENS 27
7.6 DURATION OF STUDY 29
7.7 FOLLOW-UP AFTER STUDY TREATMENT DISCONTINUATION 29

8 TREATMENT MODIFICATIONS/MANAGEMENT 30
8.1 HEMATOLOGIC TOXICITY 30
8.2 NON-HEMATOLOGIC TOXICITY 45
8.2.1 Dose Reduction Guidelines 45

8.2.2 Proteinuria 46
8.2.3 Hypertension 47
8.2.4 Hypersensitivity reactions 47
8.2.5 Modifications for hepatic toxicity 48
8.2.6 Modifications for renal toxicity 48
8.2.7 Diarrhea 48
8.2.8 Decrease in LVEF 49
8.2.9 Reversible Posterior Leukoencephalopathy Syndrome (RPLS) 49
8.2.10 Myelodysplastic Syndrome and Acute Myeloid Leukemia 50
8.2.11 Mucositis and cutaneous toxicity 50
8.2.12 Bowel obstruction 51
8.2.13 Gastrointestinal perforation or fistula 51

9 STUDY PROCEDURES **52**
9.1 SCREENING VISIT 52
9.2 ENROLLMENT CONTINGENCY PLANS 52
9.3 STUDY TREATMENT PHASE 53
9.3.1 Visit T1 (Pre-biopsy visit) 53
9.3.2 Visit T2 (post biopsy visit) 54
9.3.3 Visit T3 (Randomization visit) 54
9.3.4 Visit T4 54
9.3.5 Visit 5 and further study treatment visits (Day 1 of each chemotherapy cycle) 55
9.4 FOLLOW-UP PHASE 55
9.4.1 Visit F1 (8-weeks Clinical and Radiological Imaging follow-up visit) 55
9.4.2 Visit F2 (16-weeks follow-up visit) 56
9.4.3 Visit F3 (24-weeks follow-up visit) 56
9.4.4 Visit F4 (32-weeks follow-up visit) 56
9.4.5 Visit F5 (40-weeks follow-up visit) 57
9.4.6 Visit F6 (48-weeks follow-up visit) 57
9.4.7 Visit F7 (60-weeks follow-up visit) 58
9.4.8 Visit F8 (72-weeks follow-up visit) 58
9.4.9 Visit F9 (84-weeks follow-up visit) 58
9.4.10 Visit F10 (96-weeks - 18 months-follow-up visit – End of study Visit) 59
9.4.11 Follow-up phone calls: (Follow-up phone calls) 59
9.5 UNSCHEDULED VISITS 59
9.6 CONCOMITANT MEDICATION 59
9.7 RESCUE MEDICATION ADMINISTRATION 59
9.8 SUBJECT COMPLETION/WITHDRAWAL 60
9.8.1 Early Termination Study Visit 60

10 STUDY EVALUATIONS AND MEASUREMENTS **60**
10.1 SCREENING AND MONITORING EVALUATIONS AND MEASUREMENTS 60
10.1.1 Medical Record Review 60
10.1.2 Physical Examination 61
10.1.3 Vital Signs 61
10.1.4 Laboratory Evaluations 61
10.1.5 Other Evaluations, Measures 61
10.2 EFFICACY EVALUATIONS 62
10.3 SAFETY EVALUATION 62

11 STATISTICAL CONSIDERATIONS **62**

11.1 STATISTICAL METHODS 62
11.1.1 Baseline Data *62*
11.1.2 Efficacy Analysis *62*
11.1.3 Safety Analysis *63*
11.2 SAMPLE SIZE AND POWER 63
11.3 INTERIM ANALYSIS 63

12 STUDY MEDICATION (STUDY DEVICE OR OTHER STUDY INTERVENTION) 63
12.1 DESCRIPTION 63

13 SAFETY MANAGEMENT 63
13.1 CLINICAL ADVERSE EVENTS AND SERIOUS EVENTS 63
13.1.1 Definition of an Adverse Event (AE) *64*
13.2 ADVERSE EVENT REPORTING 64

14 STUDY ADMINISTRATION 64
14.1 TREATMENT ASSIGNMENT METHODS 64
14.1.1 Randomization *64*
14.1.2 Blinding *65*
14.1.3 Unblinding *65*
14.2 DATA COLLECTION AND MANAGEMENT 65
14.3 CONFIDENTIALITY 65
14.4 REGULATORY AND ETHICAL CONSIDERATIONS 66
14.4.1 Data and Safety Monitoring Plan *66*
14.4.2 Risk Assessment *67*
14.4.3 Potential Benefits of Trial Participation *67*
14.4.4 Risk-Benefit Assessment *67*
14.5 RECRUITMENT STRATEGY 68
14.6 INFORMED CONSENT/ASSENT AND HIPAA AUTHORIZATION 68

15 PUBLICATION OF CLINICAL DATA 69

16 REFERENCES 70

APPENDIX 74

1 ABBREVIATIONS AND DEFINITIONS OF TERMS

°C	Degrees centigrade
AE	Adverse event
ANC	Absolute Neutrophil Count
ASCO	American Society of Clinical Oncology
CAP	College of American Pathologists
CBC	Complete Blood Count
CFR	Code of Federal Regulations
CLCR	Creatinine Clearance
CLIA	Clinical Laboratory Improvement Amendments
CMP	Complete Metabolic Panel
CRF	Case Report Form
CSM	Centers for Medicare & Medicaid Services
DCC	Data Coordinating Center
DICOM	Digital Imaging and Communications in Medicine
DRE	Disease-Related Event
DSMB	Data Safety Monitoring Board
EC	Ethics Committee
ECOG	Eastern Cooperative Oncology Group
EOC	Epithelial Ovarian Cancer
FDA	Food and Drug Administration
FFR	Federal Financial Report
GCP	Good Clinical Practice
GI	Gastro-Intestinal
GIP	Gastro-Intestinal Perforation

GLP	Good Laboratory Practices
GMP	Good Manufacturing Practices
GOG	Gynecology Oncology Group
HIPAA	Health Insurance Portability and Accountability Act
HRQOL	Health Related Quality Of Life
IB	Investigator’s Brochure
ICH	International Conference on Harmonization
IDE	Investigational Device Exemption
IND	Investigational New Drug Application
IRB	Institutional Review Board
ISM	Independent Safety Monitor
LDT	Laboratory Developed Test
MOP	Manual of Procedures
MSDS	Material Safety Data Sheet
NCT	National Clinical Trial
NIH	National Institutes of Health
NIH IC	NIH Institute or Center
OHRP	Office for Human Research Protections
OS	Overall Survival
PFS	Progression Free Survival
PI	Principal Investigator
QA	Quality Assurance
QC	Quality Control
RECIST	Response Evaluation Criteria In Solid Tumors
SAE	Severe Adverse Event

SAP	Statistical Analysis Plan
SMC	Safety Monitoring Committee
SOA	Schedule of Activities
SOC	Standard Of Care
SOP	Standard Operating Procedure
UP	Unanticipated Problem
US	United States

2 ABSTRACT

Context: (Background)

The purpose of this clinical study is to confirm the utility of chemosensitivity (ChemoID) tumor testing on cancer stem cells as a predictor of clinical response in recurrent platinum-resistant epithelial ovarian cancer (EOC), fallopian tube, or primary peritoneal cancer.

Population studied will be female participants experiencing a recurrent platinum-resistant ovarian cancer (no mucinous, low grade serous, or pure sarcoma types), with ≤ 5 prior treatments, and a performance status 0-1.

Patients will be randomized between chemotherapy selected by a cancer stem cell chemotherapeutic drug cytotoxicity assay (ChemoID) versus control treatment of physician choice (chemotherapy chosen empirically by treating physician). The concept parallels testing for bacterial sensitivity against antibiotics in case of a bacterial infection. The ChemoID test utilizes sample specimens obtained following standard of care biopsy or pleural or peritoneal fluid aspiration for the treatment of ovarian cancer to test FDA approved chemotherapies indicated for the disease. No additional procedures are required to fulfill tissue collection requirements for the assay.

Objectives: (primary and important secondary objectives).

Primary study outcome measures:

- **Objective response rate (ORR) as measured by RECIST version 1.1** criteria in recurrent EOC patients who have had ChemoID-guided treatment versus physician choice control treatment (chemotherapy chosen by the Physician from the provided list).

Secondary outcome measures:

- **Progression Free Survival (PFS)**
- **Duration of Response (DOR)**
- **CA125 levels**
- **Health-Related Quality of Life (HRQOL)** as an outcome measure to ChemoID treatment selection using self-reported and validated questionnaires, addressing physical, psychological, emotional, and social issues.

Study Design:

Basic design: Parallel Group Randomized Controlled Clinical Trial.

Eligible patients will be ***randomized*** 1:1 and placed into two study arms:

- **Arm 1**: participants will be treated with control treatment (chemotherapy chosen by the treating physician from the provided list)

- **Arm 2**: participants will be treated with ChemoID-guided standard-of-care chemotherapy drugs from the provided list.

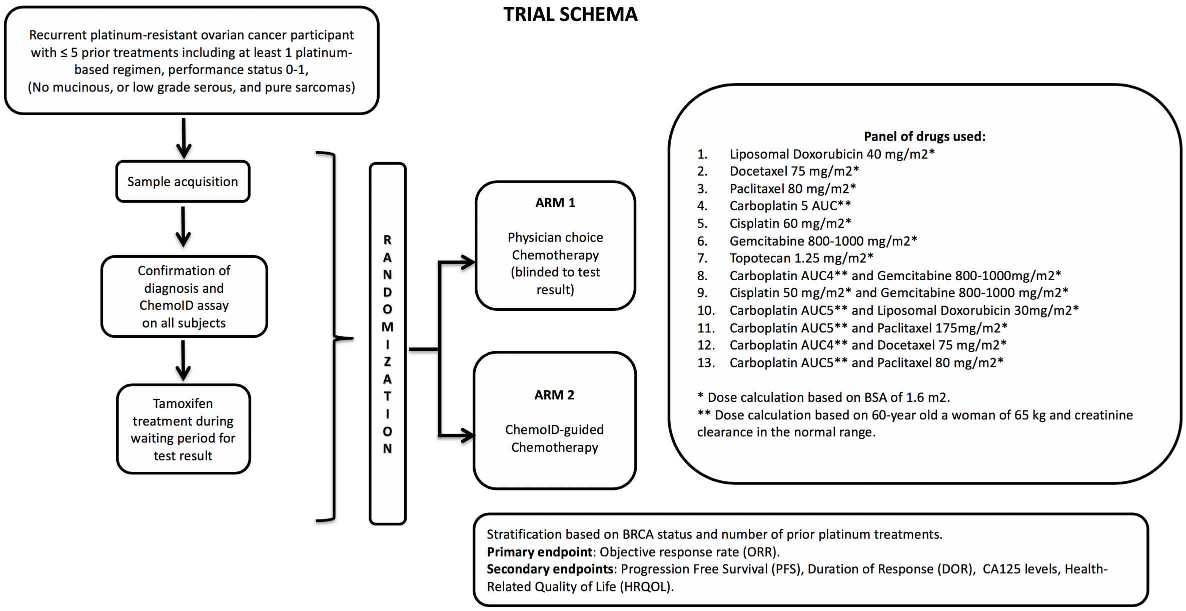

Setting/Participants:

The settings for this study are:

1. The University of Cincinnati
2. The University of Oklahoma Stephenson Cancer Center
3. MD Anderson Cancer Center
4. Duke Medical Center
5. Cleveland Clinic
6. Ohio State University
7. Arizona Oncology
8. University of Miami
9. Arizona Oncology
10. Phoenix-Biltmore Cancer Center
11. Allegheny Health Network Hospitals
12. Edwards Cancer Center, Cabell Huntington Hospital
13. Mississippi Medical Cancer Center Institute

The description of participants including key eligibility criteria will conform to the description of the two-arm study design as stated above.

Study Interventions and Measures:

Primary study outcome measures:

- **Objective response rate (ORR) as measured by RECIST version 1.1** criteria in recurrent EOC patients who have had ChemoID-guided treatment versus physician choice control treatment (chemotherapy chosen by the Physician from the provided list).

Secondary outcome measures:

- **Progression Free Survival (PFS)**
- **Duration of Response (DOR)**
- **CA125 levels**
- **Health-Related Quality of Life (HRQOL)** as an outcome measure to ChemoID treatment selection using self-reported and validated questionnaires, addressing physical, psychological, emotional, and social issues.

3 PROTOCOL SYNOPSIS

Recurrent epithelial ovarian cancer (EOC) is associated with significant mortality and a median survival rate of only 12-24 months. The vast majority of patients present with advance stage (III-IV) disease (1). Even after successful treatment and full remission, an estimated 85% of patients relapse (2).

Most relapses occur after platinum-based chemotherapy—a standard first-line treatment for EOC. Recurrent EOC is often considered either platinum-sensitive (progression-free interval >6 months after platinum-based chemotherapy) or platinum-resistant (progression-free interval ≤6 months after platinum-based chemotherapy (2).

Regimens to treat recurrent EOC are normally informed by responses to first-line therapies and vary significantly. Hundreds of clinical trials, many supported by the National Cancer Institute's consortium the Gynecologic Oncology Group (GOG), have investigated chemotherapy drugs, regimens, and cytoreductive surgery methods in search of effective strategies to prevent EOC recurrence; nonetheless, treatment regimens for recurrent platinum resistant EOC remain far less standardized than first-line therapies.

Individual responses to standardized treatments vary significantly, and eventually, resistance to platinum-based chemotherapy occurs in nearly all patients with recurrent ovarian cancer (3). When disease recurs within 6 months of the last dose of platinum, the standard treatment is sequential single-agent chemotherapy with a nonplatinum compound (3) with or without bevacizumab. Four drugs are most often used in this setting: pegylated liposomal doxorubicin, weekly paclitaxel, gemcitabine, and topotecan. All of these agents have similar response rates (10%–15%), PFS (3–4 months), and OS (~12 months); therefore, choice of which agent to use is usually based on previous treatment regimens, toxicity profile, the previous toxicities experienced by the patient, and patient preference (3).

Oncologists point to patient race/ethnicity, age, and co-morbidities, BRCA status, HRD status, as well as nuances in how EOC lesions are graded as challenges to standardization (4). Toxicity profiles are extensive for most chemotherapy drugs with no relatively low response with recurrent EOC, particularly once platinum resistance emerges. As such, there is an urgent need for ways to tailor chemotherapy regimens to patients based on their individual EOC characteristics.

ChemoID is a CLIA-certified and CAP-accredited chemotherapeutic drug cytotoxicity assay of cancer stem cells (CSCs) and primary tumor cells designed to help physicians select appropriate chemotherapy for an individual patient. The ChemoID assay uses patients' viable tumor cells to indicate which chemotherapy agent (or combinations) will kill not only cancer cells, but also importantly the cancer stem cells (CSCs) that are known to cause cancer to recur.

During the assay, cancer stem cells and bulk tumor cells from an individual patient are exposed to chemotherapy drugs. The test measures the effect of actual doses of standard-of-care chemotherapies on cancer stem cells.

The ChemoID drug response assay reports a prioritized list of effective and ineffective chemotherapies. Importantly, ChemoID assay is designed to target cancer stem

cells to mitigate relapse. The goal of the test is to improve outcomes for cancer patients, including women experiencing recurrent EOC.

The **rationale** for this study is that current treatments fail to select drugs for recurrent ovarian cancer that act on CSCs, which are likely responsible for therapy resistance, cancer recurrence and may also help explain why bulk tumor reduction following surgery and chemotherapy fail to prevent most recurrences (5-16). Targeting of CSCs alongside the bulk of other cancer cells may provide a new treatment paradigm to limit disease relapse and metastasis. This constitutes an important advantage of ChemoID approach over other assays available. By testing multiple chemotherapies on a patient's cancer stem cells and bulk tumor cells before clinically treating a cancer patient, ChemoID drug response assay may enable faster reaction time to administer the optimum selection of chemotherapy drug(s), increase patient survival, lower treatment costs, and decrease levels of toxicity by eliminating unnecessary chemotherapies.

Upon obtaining informed consent, all eligible participants affected by recurrent platinum-resistant EOC will have a tumor biopsy or malignant ascites/pleural effusion sample collection to undergo ChemoID drug response assay testing with multiple FDA approved chemotherapeutic agents.

Participants will be ***randomized*** to a standard treatment arm of chemotherapies selected by the treating physician (**Arm 1**), or to a study treatment arm with FDA approved chemotherapy drugs selected by the ChemoID drug response assay (**Arm 2**).

- **ARM 1**: participants will be treated with control treatment (chemotherapy chosen by the treating physician from the provided list)
- **ARM 2**: participants will be treated with ChemoID drug response assay guided standard-of-care chemotherapy drugs from the provided list.

A stratified randomization approach for treatment arm assignment will be used with strata based on number of prior platinum treatments, and BRCA status to ensure balance within these cells.

Participants will be assessed by chest/abdomen and pelvis CT or PET scan with contrast at 8-week intervals after therapies as per standard-of care.

Results of clinical response to standard of care chemotherapy or to ChemoID guided chemotherapy agents will be used to determine the predictive value of chemosensitivity testing with the following objectives: **Primary**: Objective Response Rate (ORR); **Secondary**: Progression Free Survival (PFS), Duration of Response (DOR), and CA125 levels. Other secondary outcomes measured will be Health-Related Quality of Life (HRQOL) as an outcome measure using a self-reported, validated questionnaire, addressing physical, psychological, emotional, and social issues).

Procedures to be performed:

The current study preferentially will utilize specimens (tumor biopsy or peritoneal/pleural fluid collection) obtained from patients having procedures for a diagnosis or for the

treatment of EOC. Alternatively, this study may utilize tumor biopsies or peritoneal/pleural fluid obtained under research protocol. Biopsies or fluid collections will be conducted by the physicians who routinely perform these procedures.

After removal, peritoneal or pleural fluid is placed in a sterile container. If a tumor biopsy is taken during the course of a surgical procedure the specimen is placed in a sterile container containing transport medium. The containers will be labeled and shipped to the ChemoID laboratory at Cabell Huntington Hospital. ChemoID assay is performed by well-trained medical technologists in a Clinical Laboratory Improvement Amendments (CLIA) certified facility under the Centers for Medicare & Medicaid Services (CMS) guidelines. The performance characteristics of the ChemoID assay in terms of its accuracy, precision, analytical sensitivity and analytical specificity are certified by both CLIA and College of American Pathologists (CAP). The clinical utility of the assay as performed is documented in several peer reviewed publications and ASCO abstracts (17-26).

**Study Title**	Standard Chemotherapy versus Cancer Stem Cell Assay Directed Chemotherapy in Recurrent Platinum Resistant Ovarian Cancer
**Funder**	(ChemoID)
**Clinical Phase**	III
**Study Rationale**	The traditional treatment course for new cases of EOC is cytoreductive surgery followed by platinum-based chemotherapy. Although this regimen is initially effective in a high percentage of cases, unfortunately most patients relapse. This is mostly attributed to the presence of ovarian cancer stem cells (CSCs), which are chemo-resistant and responsible for the recurrence of cancer. EOC, based on its biological features and clinical evolution, is a prototypical example of CSC-driven disease. Ovarian CSCs account not only for the primary tumor growth, the peritoneal spread and the relapse, but also for the development of chemoresistance, thus having profound implication for the treatment of this deadly disease. Indeed, CSCs account for a very small subpopulation in the primary tumor that is enriched in recurrent disease, both because of their expansion to fuel the relapse and because of the possible selection of drug-resistant CSCs after the first-line treatment. The ascites that develops in advanced EOC, both at diagnosis and upon recurrence, contains CSCs which are able to survive and proliferate even under non-adherent conditions, leading to self-organized spheroids of ovarian cancer cells that, in turn, account for peritoneal seeding.<br>The rationale for this study is that current treatments often fail to choose drugs for recurrent EOC that act on CSCs, which are responsible for therapy resistance and cancer recurrence. Targeting CSCs alongside the bulk of other cancer cells is a new paradigm in cancer treatment. This constitutes an important advantage of ChemoID approach over other assays available. By testing multiple

	chemotherapies on a patient's tumor cells before clinically treating a cancer patient, the ChemoID drug response assay may enable faster reaction time to administer the optimum selection of chemotherapy drug(s), increase patient survival, lower treatment costs and decrease levels of toxicity by eliminating unnecessary chemotherapies,<br>ChemoID assay is a high-complex CLIA and CAP certified drug response assay that uses a patient's viable tumor cells to indicate which chemotherapy agent will kill not only the bulk of the tumor but also the cancer stem cells (CSCs). Because CSCs are very resistant to chemotherapy and irradiation, empirical treatments often fail to choose drugs that act on CSCs, which are responsible for tumor recurrence. The ability to individualize therapy by providing the treating physician with drug response information on a panel of approved drugs should aid in the selection of effective therapy for individual patients, thus resulting in improved outcomes. The successful elimination of CSCs would have tremendous implications for the clinical management of EOC patients.<br>We would like to determine the clinical validity of chemosensitivity tumor testing on the bulk tumor cells as well as CSCs as a predictor of clinical response in the management of recurrent EOC.
**Study Objective(s)**	The **primary objective** of this study is to compare:<br>• Objective Response Rate (ORR) in patients with recurrent platinum resistant EOC who receive standard of care treatment (chemotherapy chosen by the treating physician from the provided list) versus ChemoID drug response assay-directed chemotherapy.<br>The **secondary objectives** of this study are to compare:<br>• Progression Free Survival (PFS)<br>• Duration of Response (DOR)<br>• CA125 levels<br>• Health-Related Quality of Life (HRQOL)
**Test Article(s)**<br>*(If Applicable)*	ChemoID is a high-complex CLIA and CAP certified drug response assay that uses a patient's viable tumor cells to indicate which chemotherapy agent (or "combinations") will kill not only the bulk of the cancer tumor but also the cancer stem cells (CSCs).<br>CSCs are a small sub-population of cancer cells within a patient's tumor that are very resistant to chemotherapy and radiation and are responsible for cancer recurrence. Empirical treatments often fail to

	choose drugs that act on CSCs. This may help to explain why early tumor shrinkage is often poorly predictive of overall survival. While conventional therapies kill the bulk of non-stem cancer cells, resulting in tumor shrinkage, CSCs may remain viable and later reestablish the tumor, leading to relapse. A potential new approach to address this is the targeting of both CSCs and bulk tumor cells with most effective chemotherapy choices for improved clinical outcome. ChemoID drug response assay serves as a clinically actionable tool for oncologists by measuring percent of cell kill of CSCs and bulk tumor cells by direct visualization and quantification of cell death following exposure to FDA approved chemotherapy agents. When ordering the ChemoID assay, a physician selects each of the multiple treatments under consideration for a given patient for inclusion in the test.
**Study Design**	The proposed study is a parallel group randomized controlled clinical trial. Upon obtaining informed consent, all eligible participants affected by recurrent platinum resistant EOC will have a tumor biopsy or malignant ascites/pleural effusion sample collection to undergo ChemoID drug response testing with multiple FDA-approved chemotherapeutic agents.<br><br>All participants will receive treatment with Tamoxifen while waiting chemotherapy treatment to start.<br><br>Eligible participants will be ***randomized*** to a standard treatment arm (**Arm 1**) with control treatment (chemotherapy chosen by the Physician from the provided list), or to a study arm (**Arm 2**) of FDA-approved chemotherapy selected by the ChemoID drug response assay.<br><br>• **Arm 1**: participants will be treated with control treatment (chemotherapy chosen by the treating physician from the provided list)<br><br>• **Arm 2**: participants will be treated with ChemoID-guided standard-of-care chemotherapy drug from the provided list.<br><br>A stratified randomization approach for treatment arm assignment will be used with strata based on number of prior platinum treatments, and BRCA status to ensure balance within these cells.
**Subject Population**	**Inclusion Criteria**

1. Informed consent obtained and signed.
2. Participant is willing and able to commit to study procedures including long-term follow-up visit(s);
3. Participant must be a female and at least 18 years of age at the time of enrollment.
4. Negative pregnancy test for women of childbearing potential.
5. Participant has been diagnosed with recurrent platinum resistant epithelial ovarian, peritoneal, or fallopian tube carcinoma.
6. Participant must have measurable disease by imaging or objective physical parameter.
7. Participant has agreed to provide a core biopsy of the primary site, a secondary metastatic site, or to undergo a paracentesis or thoracentesis for fluid collection.
8. An adequate fresh sample can be provided to be submitted for ChemoID testing.
9. Participant has disease of one of the following histologic epithelial cell types: high-grade serous adenocarcinoma, endometrioid adenocarcinoma, undifferentiated carcinoma, transitional cell carcinoma, clear cell carcinoma, or adenocarcinoma, not otherwise specified (N.O.S.). Cytologic confirmation of diagnosis is acceptable for participants treated with neoadjuvant therapy who have not had a surgical procedure for a histologic confirmation. Patients with low-grade serous or mucinous adenocarcinoma are not eligible, nor are patients with pure ovarian sarcomas.
10. Participant has received ≤ 5 prior regimens including at least one platinum-based regimen for their ovarian, peritoneal, or fallopian tube carcinoma.
11. Participant must have an estimated life expectancy of greater than six months, as determined by the investigator.
12. Participant requires chemotherapy and the investigator plans to administer one of the regimens of interest as deemed by her physician.

13. Participant must have an ECOG Performance Status Score of ≤ 2, KPS≥70, or 0-1 GOG status

14. Adequate laboratory values within 60 days of enrollment to study defined as follows:

    a. ANC ≥ 1500/mm3

    b. Hgb ≥ 10 mg/dl

    c. Hct ≥ 28%

    d. Platelet count ≥ 100,000/μL

    e. Serum creatinine ≤ 2.0 mg/dl

    f. Total bilirubin ≤ 2.5 mg/dl

    g. AST/SGOT ≤ 3 times ULN. If intrahepatic liver metastases are present, AST and ALT must be ≤ 5 times institutional ULN.

Exclusion Criteria

1. Use of Avastin planned to treat participant.
2. Participant has ovarian stromal, germ cell tumors or pure sarcoma.
3. Participant has borderline carcinoma (uncertain malignant potential) mucinous or low-grade serous carcinoma.
4. Participant is pregnant or lactating.
5. Participants of childbearing potential not employing adequate contraception.
6. Participants who are at risk of failure of compliance to the visit schedules and procedures including those with psychiatric disease that would substantially impact compliance or consent.
7. Estimated life expectancy of <6 months, as estimated by the investigator in consultation with participating oncologists.
8. Participants with symptomatic cardiac conditions (i.e. NYHA class III/IV or uncompensated angina).
9. Enrollment in another clinical study that precludes allowing the oncologist to select chemotherapy regimens.
10. Previously participated in this study.
11. Any condition that would, in the opinion of the investigator, place the participant at an unacceptable risk, or render the participant unable to meet the requirements of the protocol (including long-term study follow-up).

	12. CA-125 only disease without RECIST 1.1 measurable or otherwise evaluable disease.<br>13. Participant may not use any complementary or alternative medicines including natural herbal products or folk remedies as they may interfere with the effectiveness of the study treatments.<br><br>Participants who do not meet all of the enrollment criteria may not be enrolled. Any violations of these criteria must be reported in accordance with IRB Policies and Procedures.
**Number Of Subjects**	Total Number of Subjects (220)<br>Total Number of Sites (13)
**Study Duration**	Each subject's participation will last up to 24 months at the most<br>The entire study is expected to last 3 years
**Study Phases:**	3 phases
**1- Screening:**	1) Screening for eligibility and obtaining consent
**2- Treatment:**	2) Drainage of fluid or biopsy of tumor of suspected malignant pleural or peritoneal tumor followed by physician choice treatment of chemotherapies vs. ChemoID-guided treatment in recurrent EOC patients
**3- Follow-Up:**	3) Study visits with clinical assessment at the beginning of every cycle of chemotherapy and imaging assessment with chest/abdomen/pelvis CT scan preferentially with contrast once every 8 weeks (+/- 7 days) for the first year and every 12 weeks (+/- 7 days) after the first year as per standard of care.
**Efficacy Evaluations**	Primary outcome: objective response rate (ORR).<br>Secondary outcomes: progression free survival (PFS), duration of response (DRR), CA125 levels, and Health-Related Quality of Life (HRQOL).
**Pharmacokinetic Evaluations**	(N/A)
**Safety Evaluations**	Chemotherapies used are FDA approved to treat this disease and are part of standard of care
**Statistical And Analytic Plan**	The primary endpoint of improved response rate will be examined using Cox Proportional Hazards Model (PHM) regression under an intention to treat analysis.

**DATA AND SAFETY MONITORING PLAN**	The PI and a DSMB will be responsible to monitor data quality management and ongoing assessment of safety

TABLE 3.1: SCHEDULE OF STUDY PROCEDURES

**Study Phase**	**Screening**	**Treatment/Intervention**					**Clinical follow-up at the beginning of each chemotherapy cycle and imaging follow-up every 8 weeks as per standard of care**										**Follow-up phone call**	
**Visit Number**		**T1**	**T2**	**T3**	**T4**	**T5**	**F1**	**F2**	**F3**	**F4**	**F5**	**F6**	**F7**	**F8**	**F9**	**F10**	**C1**	**C2**
**Week Number**							**w8**	**w16**	**w24**	**w32**	**w40**	**w48**	**w60**	**w72**	**w84**	**w96**		
**Follow-up phone call**																	**X**	**X**
Review Inclusion/Exclusion Criteria	X																	
Informed Consent/Assent	X																	
Demographics/Medical History	X																	
Physical Examination	X	X	X	X	X	X	X	X	X	X	X	X	X	X	X	X		
Vital Signs: BP, HR, RR	X	X	X	X	X	X	X	X	X	X	X	X	X	X	X	X		
Height and Weight	X				X	X	X	X	X	X	X	X	X	X	X	X		
Pregnancy Test	X																	
Prior/Concomitant Medications	X				X	X	X	X	X	X	X	X	X	X	X	X		
Clinical Laboratory Evaluation Including CA125 levels *	X				X	X	X	X	X	X	X	X	X	X	X	X		
Abdomen and pelvis CT scan with or w/o contrast **and/or** anyone or more of the following imaging exams: Chest CT scan, PET/CT scan, MRI, chest X-Ray, and abdomen ultrasound	X		X				X	X	X	X	X	X	X	X	X	X		
Clinical Assessment	X		X	X	X	X	X	X	X	X	X	X	X	X	X	X		
Confirm Eligibility	X																	
Scheduling for Tumor Biopsy or Peritoneal/Abdominal Fluid collection for Diagnosis and		X																

for ChemoID (at the same time)																		
Review of Pathology Report			X															
Tamoxifen treatment prior to start cycle 1 chemo**			X	X	X													
Study coordinators will contact the ChemoID lab to verify that tumor biopsy is viable and growing			X															
Randomization of patients who have provided a viable and growing sample to the ChemoID lab				X														
Study coordinators will contact the ChemoID lab to request release of ChemoID assay results for patients enrolled in ChemoID-guided arm				X														
Review of ChemoID drug response assay for participants assigned to assay-guided Arm					X													
Dispense Chemotherapy Drug as per Study Arm						X	X	X	X	X	X	X	X	X	X	X		
Concomitant Therapy			X	X	X	X	X	X	X	X	X	X	X	X	X	X		
Drug Compliance ***			X	X	X	X	X	X	X	X	X	X	X	X	X	X		
Adverse Event Assessment					X	X	X	X	X	X	X	X	X	X	X	X		

* Lab values are acceptable within 60 days of enrolment. Enrollment of subject happens after confirming with the ChemoID lab that the cancer cells are growing and that the sample is not contaminated.

* Tamoxifen should continue until the day prior to IV chemotherapy

** Confirm verbally drug compliance

TABLE 3.2: STUDY DIAGRAM

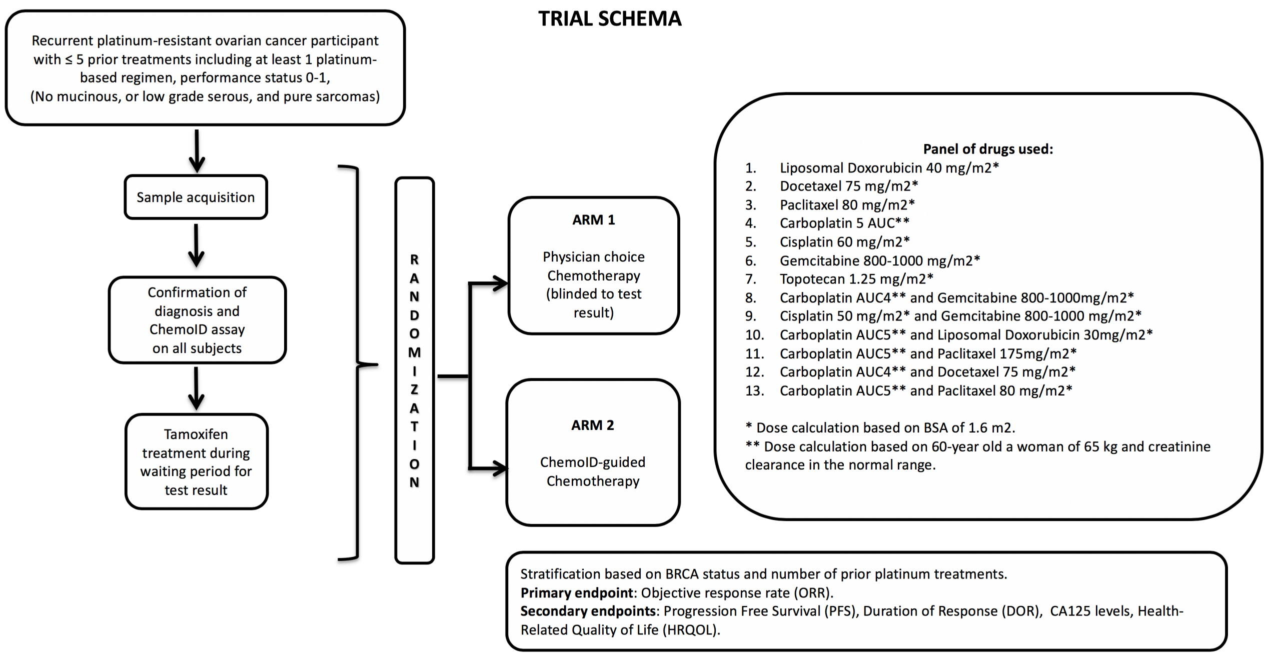

4 BACKGROUND INFORMATION AND RATIONALE

4.1 Introduction

Most patients with epithelial ovarian cancer (EOC) present with advanced disease (stage III-IV) at diagnosis (1, 27). Recurrent EOC is associated with significant mortality and a median survival of only 12-24 months (**Figure 4.1**) (1).

The combination of platinum drugs with a taxane is the standard of care for the systemic treatment after primary cytoreductive surgery (28). However, this treatment results in response rate (RR) of ~70% in patients with suboptimally-debulked disease, and of ~80% in optimally cytoreduced patients (29, 30). Disease recurrence is common in these patients, and most of them eventually develop platinum resistant disease (defined as disease recurring within 6 months after last receipt of platinum-based chemotherapy) (2, 31).

RRs and duration of response to second line chemotherapy for patients with recurrent platinum-resistant disease are significantly lower than those with platinum sensitive disease. In women with platinum-resistant disease, RRs range from 10%-15%, and duration of response is typically less than 6 months, with PFS (3–4 months), and OS (~12 months) to chemotherapeutic agents such as pegylated liposomal doxorubicin (PLD), topotecan, taxanes, etoposide, and gemcitabine (3). In comparison, the RRs are usually >30% and/or duration of response >8 months in women with platinum-sensitive disease (32, 33).

Late-line treatment options for patients with ovarian cancer are few, with the proportion of patients achieving an overall response typically less than 10%, and median overall survival after third-line therapy of 5–9 months (34).

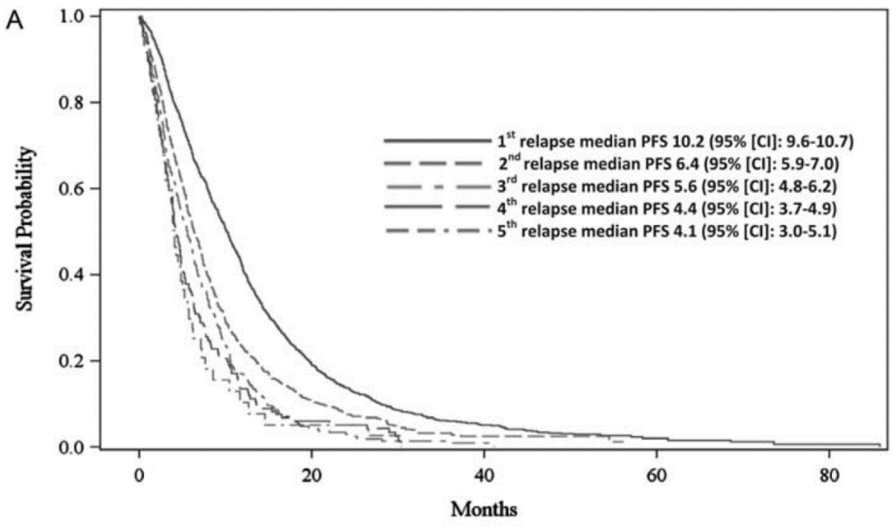

Figure 4.1: *Kaplan-Meier analyses of progression-free survival (PFS) across 3,388 patients with 1st-5th relapses of EOC (Hanker, et al., 2012).*

Regimens to treat recurrent EOC are normally informed by responses to first-line therapies and vary significantly; therefore, choice of which agent to use is usually based on toxicity profile, the previous toxicities experienced by the patient, and patient preference (3).

Hundreds of clinical trials, many supported by the National Cancer Institute's consortium the Gynecologic Oncology Group (GOG), have investigated chemotherapy drugs, regimens, and reductive surgery methods in search of effective strategies to prevent EOC recurrence. Recently, several phase 3 randomized trials have assessed combination therapy with a targeted agent such as bevacizumab for recurrent platinum-resistant and –sensitive ovarian cancer (i.e. AURELIA, OCEANS, and GOG-0213) (35-39).

In particular, the AURELIA trial assessed bevacizumab combined with chemotherapy - either liposomal doxorubicin, weekly paclitaxel, or topotecan – versus chemotherapy alone in patients with recurrent platinum resistant ovarian cancer with 1-2 prior chemotherapy regimens. For patients receiving bevacizumab/chemotherapy, the primary endpoint of PFS was significantly prolonged (6.8 months versus 3.4 months, HR 0.48, 95% CI 0.38-0.60, p<0.001) than in patients treated with chemotherapy alone. Figure 4.1.1 shows the Kaplan-Mayer analysis of PFS in the AURELIA trial.

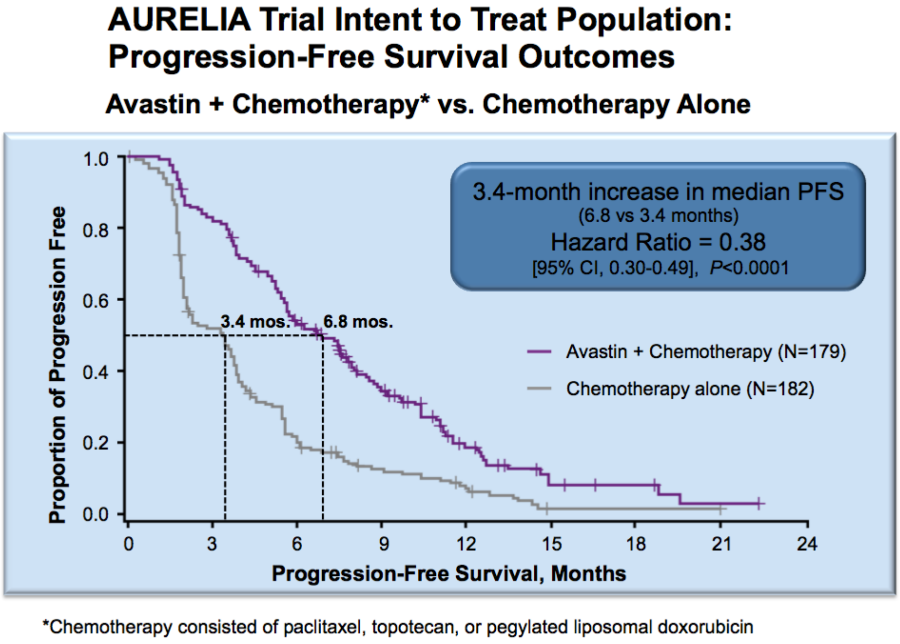

Figure 4.1.1: *Kaplan-Meier analysis of PFS in the AURELIA intent to treat trial.*

The median overall survival was 16.6 months for the bevacizumab/chemotherapy versus 13.3 months for chemotherapy alone; the overall survival was similar between groups (HR 0.85,95% CI, 0.66-1.08, p<0.174).

However, none of these trials have addressed or explored the idea of reducing the load of cancer stem cells in recurrent EOC to enable a greater and more durable response to therapy. Despite results demonstrating treatment advances, regimens for platinum-resistant recurrent EOC are unfortunately not curative. Thus, there is **urgent need for the development of alternative strategies** given the poor response of platinum-resistant recurrent ovarian epithelial malignant disease.

Individual patient responses to standardized treatments vary significantly. Oncologists point to patient race/ethnicity, age, and co-morbidities, as well as nuances in how EOC lesions are graded as challenges to standardization (4). Toxicity profiles are extensive for most chemotherapy drugs with no guarantee of success at the patient level. As such, there is an urgent need for ways to tailor chemotherapy regimens to patients based on their individual EOC characteristics.

ChemoID is a CLIA-certified and CAP-accredited drug response assay designed to help physicians' select appropriate chemotherapy for an individual patient. The ChemoID drug response assay uses patient's live tumor cells to indicate which chemotherapy agent (or combinations) will kill not only cancer cells, but also more importantly the cancer stem cells (CSCs) that are known to cause cancer to recur.

During the assay, cancer stem cells and bulk tumor cells from an individual patient are exposed to FDA-approved chemotherapy drugs. The drug response assay measures the effect of actual doses of standard-of-care chemotherapies on cancer stem cells. The ChemoID assay reports a prioritized list of effective and ineffective chemotherapies.

Importantly, the ChemoID drug response assay is designed to target cancer stem cells to mitigate relapse. The goal of the assay is to improve outcomes for cancer patients, including women experiencing recurrent platinum-resistant EOC.

The **rationale** for this study is that current treatments often fail to choose drugs for recurrent ovarian cancer that act on CSCs, which are responsible for therapy resistance and cancer recurrence.

Targeting of CSCs alongside the bulk of other cancer cells is a promising strategy towards the management and eradication of ovarian cancer. This constitutes an important advantage of ChemoID approach over other assays available. By testing multiple chemotherapies on a patient's tumor cells before clinically treating a cancer patient, ChemoID drug response assay may enable faster reaction time to administer the optimum selection of chemotherapy drug(s), increased patient survival, lower treatment costs, and decreased levels of toxicity by eliminating unnecessary chemotherapies.

4.2 Name and Description of Investigational Product or Intervention

ChemoID is a CLIA-certified and CAP-accredited assay performed by medical technologists at the Cabell Huntington Hospital in West Virginia under the Clinical Laboratory Improvement Amendments of 1988 (CLIA) regulations.

Both CLIA and College of American Pathologists (CAP) certified the performance characteristics of the ChemoID assay in terms of its accuracy, precision, analytical sensitivity and analytical specificity. The clinical utility of the assay as performed is documented in several peer reviewed publications and ASCO abstracts (5-14).

For the following reasons ChemoID drug response assay is considered a non-significant risk (NSR) device:

1) Use of the assay involves sampling of a subjects' tumor. This sampling is not considered an additional or unnecessary risk as surgeons routinely perform standard biopsy procedures or resection of tumors. The current study uses fresh specimens from tumor biopsies or peritoneal/pleural fluid collections obtained under the standard of care. Biopsies will be conducted by the physicians who routinely perform these procedures. Tissue for this study will be obtained by a physician under the standard of care procedure or after patient consent under the research protocol after it is assured that there is adequate tissue for routine histologic analysis.

2) The ChemoID assay is performed by medical technologists in a CLIA certified facility.

3) Biopsy samples obtained upon informed consent undergo ChemoID assay with FDA approved chemotherapeutic agents. No investigational agents are included in the assay. Chemotherapy drugs associated with potential clinical benefit are ranked in order of potential or predicted efficacy against bulk tumor and cancer stem cell populations.

4) Information contained in the ChemoID assay report is considered by the physician in conjunction with all other relevant patient's health information to make treatment decisions.

The ChemoID drug response assay uses patient's live tumor cells to indicate which chemotherapy agent (or combinations) will kill not only cancer cells, but also and more importantly, the cancer stem cells (CSCs) that are known to cause cancer to recur.

During the assay, cancer stem cells and bulk tumor cells from an individual patient are exposed to FDA-approved chemotherapy drugs. The drug response assay measures the cell kill effect of actual doses of standard-of-care chemotherapies on cancer stem cells (**Figure 4.2.1**).

The ChemoID assay results include a prioritized list of effective and ineffective chemotherapies based on actual percent cell kill measured by the assay. An actual redacted ChemoID drug response assay delivered to clinicians is shown in **Figure 4.2.1**. Importantly, ChemoID assay is designed to target cancer stem cells to mitigate relapse. The goal of the drug response assay is to improve outcomes for cancer patients, including women experiencing recurrent platinum resistant EOC.

ChemoID® Drug Response Assay
Cabell Huntington Hospital Laboratory

1340 Hal Greer Blvd
Huntington, WV 25701
304-526-2145

Cabell Huntington Hospital

Patient Information

PATIENT NAME	DOB	PRN	MRN	BN

ORDERING PHYSICIAN	PHYSICIAN FACILITY

Specimen Information

TUMOR	SPECIMEN SITE	SPECIMEN TYPE
Adenocarcinoma	Peritoneal	Fluid (Peritoneal)
SPECIMEN COLLECTED DATE	LABORATORY RECEIVED DATE	REPORT DATE

ChemoID® Results

Comparative Values for Bulk of Tumor

Treatment	% Cell Kill	Graphic Comparison
Docetaxel 75 mg/m2 + Doxorubicin 60 mg/m2	98.3 % ± 0.1	
Paclitaxel 175 mg/m2 + Cisplatin 100 mg/m2	97.1 % ± 0.2	
Doxorubicin 60 mg/m2 + Gemcitabine 1000 mg/m2	96.2 % ± 0.3	
Cyclophosphamide 600 mg/m2 + Cisplatin 100 mg/m2	95.7 % ± 0.1	
Pemetrexed 500 mg/m2 + Cisplatin 100 mg/m2	95.2 % ± 0.4	
Fluorouracil 1000 mg/m2 + Cisplatin 100 mg/m2	95.2 % ± 0.1	
Cisplatin 100 mg/m2	91.0 % ± 1.4	
Doxorubicin 60 mg/m2	90.7 % ± 1.0	
Gemcitabine 1000 mg/m2 + Carboplatin 360 mg/m2	89.2 % ± 0.4	
Cyclophosphamide 600 mg/m2 + Carboplatin 360 mg/m2	88.0 % ± 0.7	
Carboplatin 360 mg/m2	38.6 % ± 1.0	
Docetaxel 75 mg/m2	13.2 % ± 1.0	
Fluorouracil 1000 mg/m2	<10 %	
Gemcitabine 1000 mg/m2	<10 %	
Pemetrexed 500 mg/m2	<10 %	
Paclitaxel 175 mg/m2	<10 %	
Cyclophosphamide 600 mg/m2	<10 %	
Topotecan 1.5 mg/m2	<10 %	

Comparative Values for Cancer Stem-Like Cells

Treatment	% Cell Kill	Graphic Comparison
Docetaxel 75 mg/m2 + Doxorubicin 60 mg/m2	94.5 % ± 0.1	
Paclitaxel 175 mg/m2 + Cisplatin 100 mg/m2	93.6 % ± 0.1	
Doxorubicin 60 mg/m2 + Gemcitabine 1000 mg/m2	90.1 % ± 0.4	
Cyclophosphamide 600 mg/m2 + Cisplatin 100 mg/m2	90.1 % ± 0.1	
Pemetrexed 500 mg/m2 + Cisplatin 100 mg/m2	89.3 % ± 0.3	
Fluorouracil 1000 mg/m2 + Cisplatin 100 mg/m2	89.3 % ± 0.3	
Doxorubicin 60 mg/m2	83.6 % ± 0.9	
Cisplatin 100 mg/m2	83.3 % ± 1.0	
Gemcitabine 1000 mg/m2 + Carboplatin 360 mg/m2	81.8 % ± 0.2	
Cyclophosphamide 600 mg/m2 + Carboplatin 360 mg/m2	67.7 % ± 1.4	
Docetaxel 75 mg/m2	57.0 % ± 1.2	
Carboplatin 360 mg/m2	12.9 % ± 1.5	
Paclitaxel 175 mg/m2	<10 %	
Fluorouracil 1000 mg/m2	<10 %	
Gemcitabine 1000 mg/m2	<10 %	
Topotecan 1.5 mg/m2	<10 %	

Figure 4.2.1: *Anonymized ChemoID drug response assay results for an Ovarian Cancer.*

4.3 Findings from Previous Clinical Studies

Several clinical studies have been conducted on the use of ChemoID drug response assay to predict chemotherapy sensitivity for different types of cancer.

In a prospective clinical investigation the ChemoID assay was used to measure the sensitivity and resistance of CSCs and bulk of tumor cells cultured from 42 glioblastoma (GBM) clinical samples challenged with several chemotherapy agents, which were correlated to the clinical response of the treated patients, independently of other biomarkers. Patients were all treated with standard-of-care TMZ plus radiation with or without maximal surgery, depending on the status of the disease. Patients were prospectively monitored for tumor response, time to recurrence, progression-free survival (PFS), and overall survival (OS). Odds ratio (OR) associations of 12-month recurrence, PFS, and OS outcomes were estimated for CSC, bulk tumor, and combined assay responses for the standard-of-care TMZ treatment; sensitivities/specificities, areas under the curve (AUCs), and risk reclassification components were examined.

Clinical Study Results: Median follow-up was 8 months (range 3-49 months). For every 5% increase in *in vitro* CSC cell kill by TMZ, 12-month patient response (non-recurrence of cancer) increased two-fold, OR = 2.2 (P = .016). Similar but somewhat less supported associations with the bulk tumor test were seen, OR = 2.75 (P = .07) for each 5% bulk tumor cell kill by TMZ. Combining CSC and bulk tumor assay results in a single model yielded a statistically supported CSC association, OR = 2.36 (P = .036), but a much attenuated remaining bulk tumor association, OR = 1.46 (P = .472). AUCs and [sensitivity/specificity] at optimal outpoints (N40% CSC cell kill and N55% bulk tumor cell kill) were AUC = 0.989 [sensitivity = 100/specificity = 97], 0.972 [100/89], and 0.989 [100/97] for the CSC only, bulk tumor only, and combined models, respectively. Risk categorization of patients was improved by 11% when using the CSC test in conjunction with the bulk test (risk reclassification nonevent net reclassification improvement [NRI] and overall NRI = 0.111, P = .030). Median recurrence time was 20 months for patients with a positive (N40% cell kill) CSC test versus only 3 months for those with a negative CSC test, whereas median recurrence time was 13 months versus 4 months for patients with a positive (N55% cell kill) bulk test versus negative. Similar favorable results for the CSC test were observed for PFS and OS outcomes.

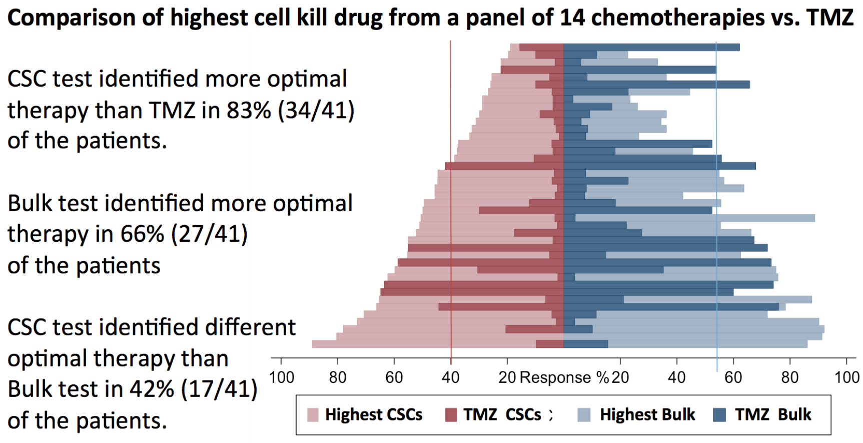

Figure 4.3.1: *Comparison of most sensitive drug from a panel of various chemotherapies vs. Temodar*

Results across a panel of 14 potential other treatments indicated that 34/41 (83%) potentially more optimal alternative therapies may have been chosen using CSC results, whereas 27/41 (66%) alternative therapies may have been chosen using bulk tumor results.

A pyramid plot of percent cell kill for the most cytotoxic drug and TMZ comparing CSC and bulk tests for each patient is illustrated above. Optimal therapies with the highest cell kill are shown in light colors and TMZ cell kill is shown in dark colors with each row of the pyramid corresponding to results for a single patient. When the light bar is longer than the dark bar, a potentially more optimal therapy than TMZ is identified. CSC results outlined in red show patients whose CSC test identified an optimal therapy that was different than the optimal therapy identified by the bulk test, 17/41 patients, 42% (95%CI 26-57%) p<0.001.

Figure 4.3.2 illustrates the relationship between the TMZ CSC assay results (%-cell kill on the y -axis) and TMZ bulk tumor assay results (%-cell kill on the x -axis) characterized by 12-month recurrence outcomes, with solid circles representing treatment responders (patients who did not manifest a recurrence at 12 months) and open circles representing patients manifesting recurrence within 12 months from treatment. Referent lines are drawn at the optimal thresholds from the logistic regression models (40% for CSC, 55% for bulk tumor). In the upper-right quadrant are patients with high TMZ cell kill for both CSC and bulk tumor assays where 5/41 (12%) had prolonged tumor response and only 1 (2.4%) was recurrent. In the lower-left quadrant are patients with low TMZ cell kill for both CSC and bulk tumor assays; all 31 (76%) recurred within 12 months from treatment. Finally, the lower-right quadrant shows patients whose TMZ bulk tumor assay showed a high cell kill (> 55%) but whose TMZ CSC assay showed a low cell kill (< 40%); all 4/41 (10%) recurred within 12 months.

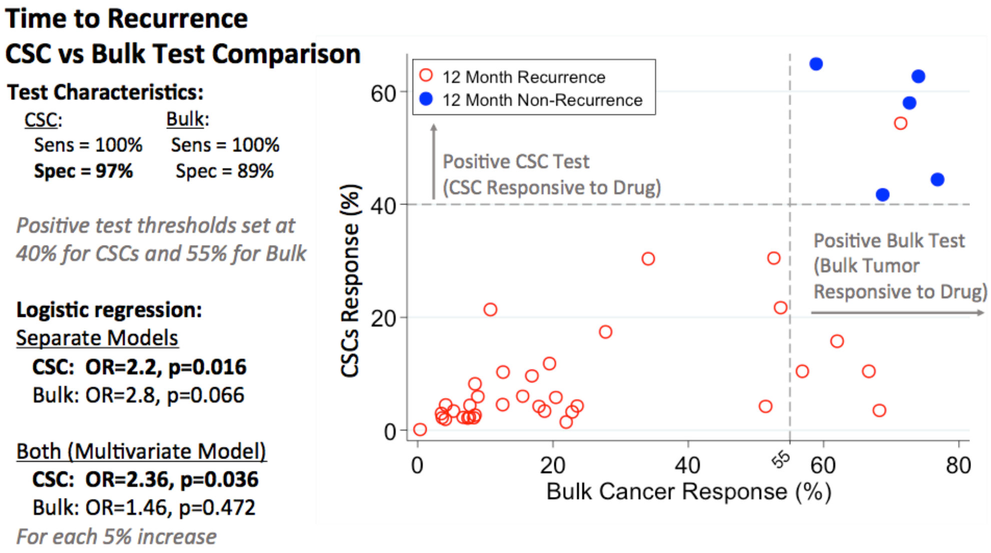

Figure 4.3.2: *Quadrant diagram of the relationship between TMZ CSC assay results (%-cell kill on the y-axis) and TMZ bulk tumor assay results (%-cell kill on the x-axis) characterized by 12-month recurrence outcomes.*

In figure 4.3.2 solid circles represent treatment responders (patients who did not manifest a recurrence at 12 months), and open circles represent patients manifesting recurrence within 12 months from treatment. Optimal threshold referent lines from the logistic regression models (40% for CSC, 55% for bulk tumor) are illustrated. When the CSC assay results were considered separately, every 5% increase in TMZ CSC %-cell kill

was associated with a significant two-fold increase in 12-month patient response (non-recurrence of cancer), OR = 2.2 (95% CI 1.16-4.17), *P* = .016.

TMZ bulk tumor %-cell kill was similarly associated but with less statistical support, OR = 2.8 (0.93-8.06) *P* = .066 for each 5% increase.

When the CSC and bulk test results were analyzed together in a single, multivariate model, the CSC test again showed a statistically supported OR of 2.36 (1.06-5.25) *P* = .036, whereas the bulk of tumor test association fell to OR = 1.46 (0.52-4.08) *P* = .472.

ChemoID assay areas under the curve were high for all three models: AUC = 0.989, 0.972, and 0.989 for the separate CSC, bulk tumor, and combined model, respectively. Related optimal thresholds for the assays were 40% CSC cell kill and 55% bulk tumor cell kill by TMZ which then provided sensitivities/specificities of 100/97, 100/89, and 100/97 for the three models, respectively. Both the CSC assay and the bulk tumor assay performed well in these models and in related secondary models for OS & PFS, with the CSC assay showing slightly improved results over the bulk tumor results throughout.

Figure 4.3.3 shows Kaplan-Meyer plots of time to recurrence stratified by ChemoID test results from the clinical study using the optimal TMZ thresholds (40% cell kill for CSC and 55% cell kill for bulk tumor). Patients with positive ChemoID CSC tests (>40% cell kill) had longer median times to recurrence (20 months) than those with negative CSC tests (3 months). Patients with positive bulk tumor tests (>55% cell kill) had longer median times to recurrence (13 months) than those with negative bulk tumor tests (4 months), but the separation was not as great.

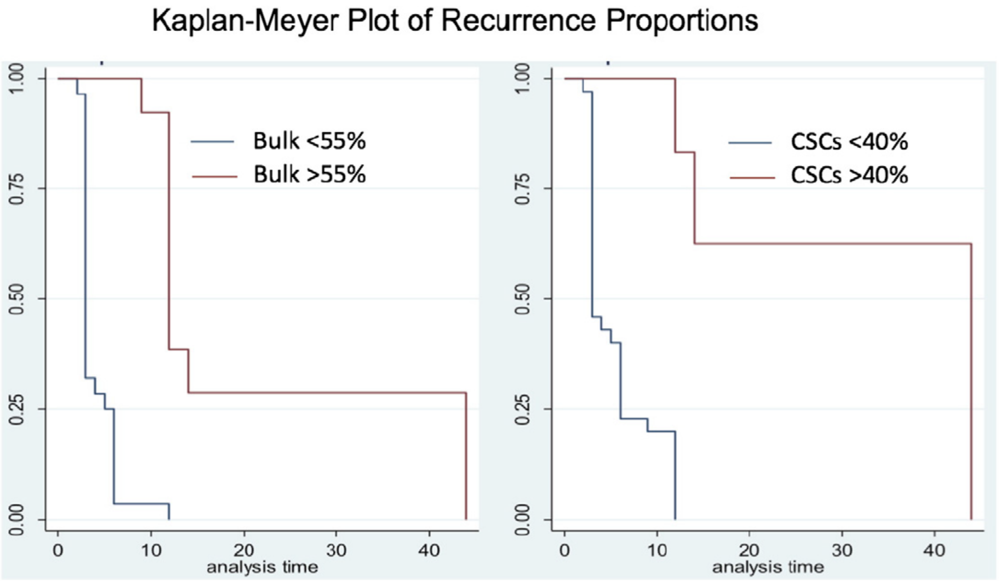

Figure 4.3.3: *Kaplan-Meier plots of tumor recurrence across the study period. Survival (tumor non recurrence) is shown stratified by dichotomized test results (TMZ optimal thresholds of CSCs> 40% and bulk test >55%); P for both <0.001 in Cox proportional hazard models.*

The ChemoID drug response assay has also been used prospectively to select treatment for 45 patients affected by poor prognosis (3rd - 5th relapse) recurrent ovarian cancer. Test results from ChemoID assay to measure the sensitivity and resistance of CSCs and bulk of tumor cells challenged with 15 chemotherapy single agents or their combinations were correlated to the clinical response of the treated patients, independently of other biomarkers. Patients were all treated with a regimen

guided by the ChemoID test, taking into consideration patients' health status and using dose reductions, as needed. CT and PET scans were used to prospectively monitor patients for tumor response, time to recurrence, progression-free survival (PFS), and overall survival (OS).

In our pilot study (see Table 4.3.1) we found that recurrent ovarian cancer patients (3rd - 5th relapse) prospectively treated with ChemoID-guided chemotherapy had a median PFS of 9.5 months – 3rd relapse; 7 months – 4th relapse; and 6.5 months -5th relapse, respectively, compared against historical data showing PFS of 5.6 months – 3rd relapse; 4.4 months – 4th relapse; and 4.1 months -5th relapse, respectively (1).

Table 4.3.1. *Comparison of historical median PFS and ChemoID-guided treated poor prognosis ovarian cancer patients*

Relapse	Historical Median PFS (months)	ChemoID Median PFS (months)
3rd	5.6	9.5
4th	4.4	7
5th	4.1	6.5

Figure 4.3.4 illustrates the relationship between the CSC assay results (%-cell kill on the y-axis) and bulk tumor assay results (%-cell kill on the x-axis) characterized by recurrence outcomes at 6-month, with blue solid circles representing treatment responders (non-recurrence at 6 months) and red open circles representing patients with recurrence within 6 months from treatment start. Referent lines are drawn at thresholds of 40% for CSC, and 55% cell kill for bulk tumor. In the upper-right quadrant are represented patients treated with high-cell kill chemotherapy for both CSC and bulk tumor assays, who were non-recurrent at 6 months. In the lower-left quadrant are represented patients treated with low- cell kill chemotherapy for both CSC and bulk tumor assays, who had a recurrence in less than 6 months. The CSC test demonstrated 97% sensitivity, 85% specificity, 93% positive predictive value (PPV), and 90% negative predictive value (NPG). The bulk test demonstrated 81% sensitivity, 77% specificity, 91% PPV, and 65% NPG.

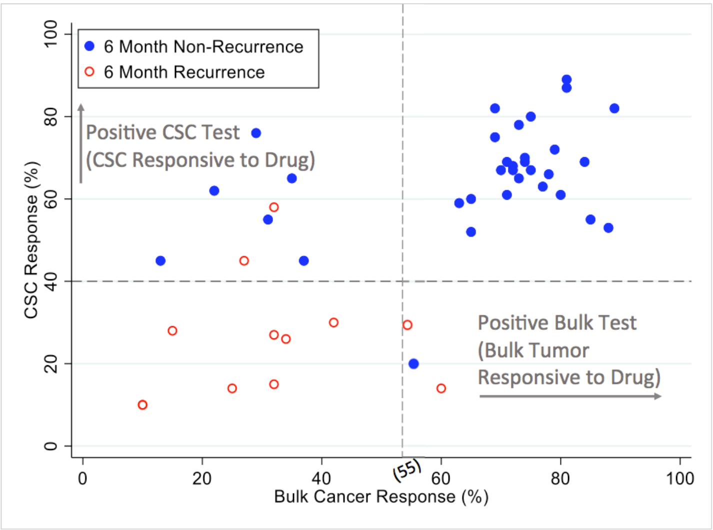

Figure 4.3.4: *Quadrant diagram of the relationship between CSC assay results (%-cell kill on the y-axis) and bulk tumor assay results (%-cell kill on the x-axis) characterized by 6-months recurrence outcomes.*

Figures 4.3.5 and 4.3.6 show Kaplan-Meier plots of overall survival across the study period stratified by dichotomized test results on bulk test or CSC test respectively.

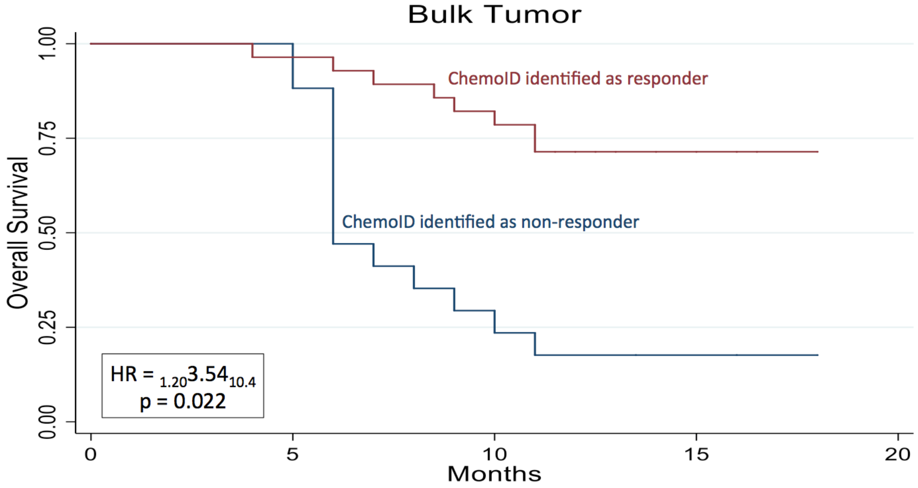

***Figure 4.3.5**: Kaplan-Meier plots of overall survival across the study period. Survival is shown stratified by dichotomized test results (bulk test >55% cell kill - responder or <55% cell kill – non-responder); HR=3.54; P=0.022 in Cox proportional hazard models.*

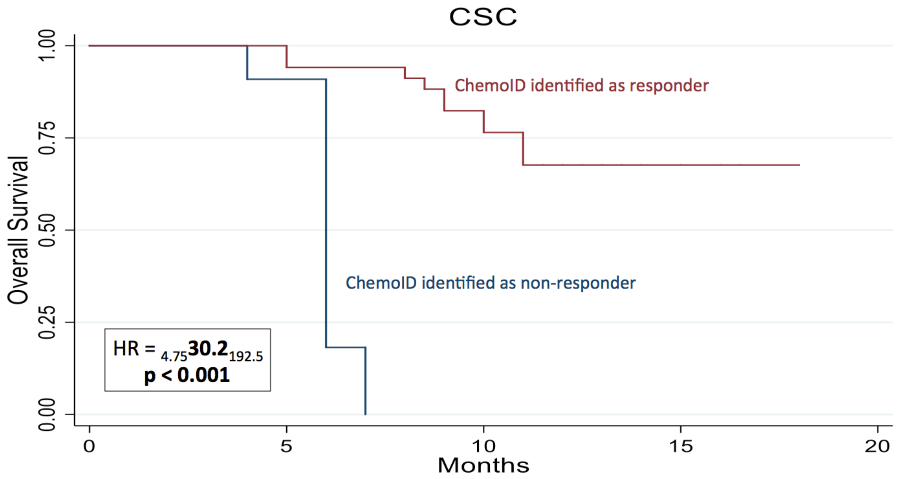

***Figure 4.3.6**: Kaplan-Meier plots of overall survival across the study period. Survival is shown stratified by dichotomized test results (CSC test >40% cell kill - responder or <40% cell kill – non-responder); HR=30.2; P<0.001 in Cox proportional hazard models.*

In the CSC model, the ovarian cancer patients that ChemoID identified as non-responders had over 30 times the hazard of death compared to those women that were identified by ChemoID as responders (p<0.001). Moreover, we observed that adding the ChemoID bulk tumor and CSC response to a Cox model containing only relapse number and women age increased Harrell's C statistics from 0.66 to 0.89 for overall survival.

Figures 4.3.7 and 4.3.8 show two examples of case report images from our case series of advanced ovarian cancers showing correlation between ChemoID high-suppression chemotherapy drug(s) combinations and clinical response of patients treated with ChemoID-guided therapy.

Figure 4.3.7: MRI images from a recurrent ovarian patient treated with ChemoID-guided 4th line therapy

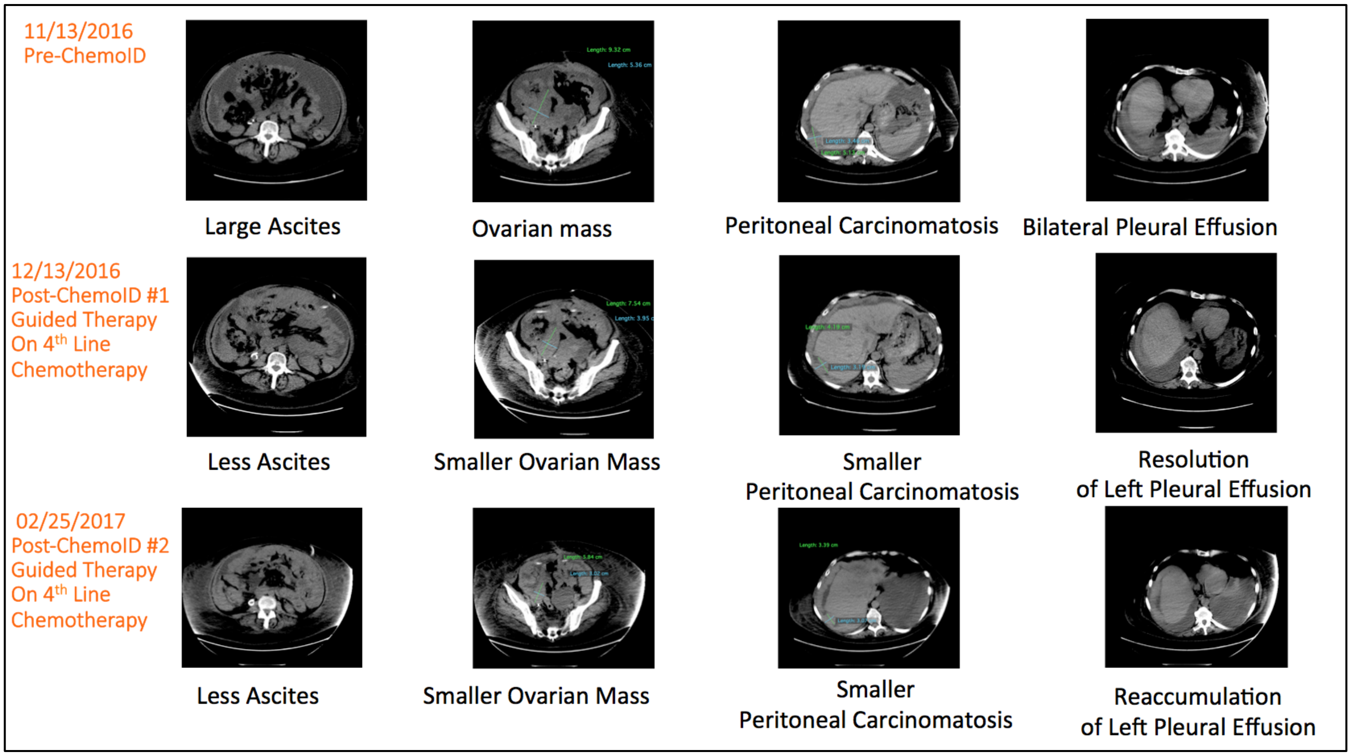

Figure 4.3.8: MRI images from a recurrent ovarian patient treated with ChemoID-guided 5th line chemotherapy

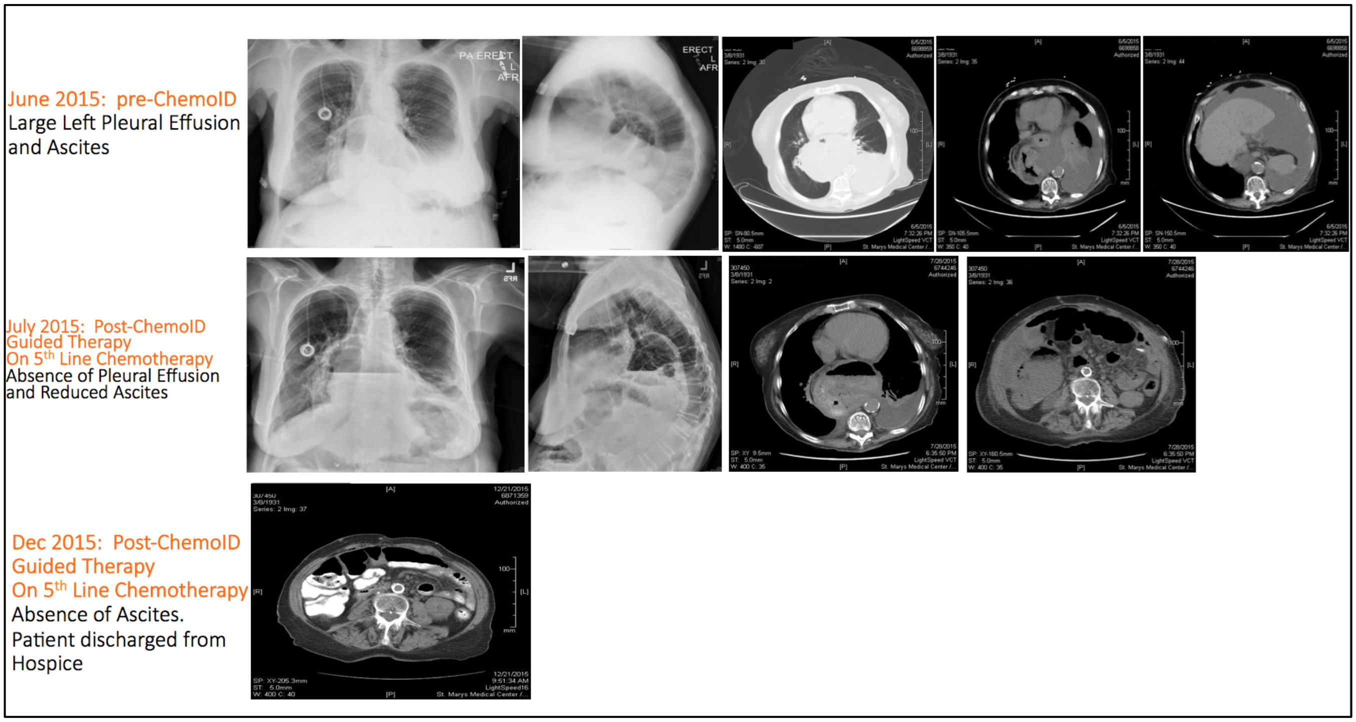

4.4 ChemoID Selection of Drugs and Dosages

Drugs and dosages used in the standard panel of chemotherapies tested in the ChemoID drug response assay are all FDA approved to treat recurrent ovarian cancer. A list of current chemotherapy agents used to treat recurrent ovarian cancer are presented in **Table 4.4.1 A and B**.

Table 4.4.1A. List of current single chemotherapeutic agents used in standard of care to treat recurrent epithelial ovarian carcinoma and on the standard ChemoID panel

Regimen	Single Drug	Dose and schedule
**1**	Liposomal Doxorubicin	40mg/m2* q 28 days
**2**	Docetaxel	75mg/m2* q21 days
**3**	Paclitaxel	80mg/m2* D1, 8, 15 of 28 day cycles
**4**	Carboplatin	AUC 5** q 21 days
**5**	Cisplatin	60mg/m2* q 21 days
**6**	Gemcitabine	800-1000mg/m2* day 1 & 8 of 21 day cycles
**7**	Topotecan	1.25 mg/m2* day 1-5 q21 days

Table 4.4.1B. List of current chemotherapeutic agent combinations used in standard of care to treat recurrent epithelial ovarian carcinoma and on the standard ChemoID panel

Regimen	Single Drug	Dose and schedule
**8**	Carboplatin<br>Gemcitabine	AUC 4** D1 q21 days<br>800 - 1000 mg/m2* D1 & 8 q21 days
**9**	Cisplatin<br>Gemcitabine	50 mg/m2* D1 & 15 q28 days<br>800-1000 mg/m2* D1 & 15 q28 days
**10**	Carboplatin<br>Liposomal Doxorubicin	AUC 5** D1 q28 days<br>30 mg/m2* D1 q28 days
**11**	Carboplatin<br>Paclitaxel	AUC 5** D1 q21 days<br>175 mg/m2* D1 q21 days
**12**	Carboplatin<br>Docetaxel	AUC 4** D1 q21 days<br>75 mg/m2* D1 q21 days
**13**	Carboplatin<br>Paclitaxel	AUC 5** D1 q28 days<br>80 mg/m2* D1, 8, 15

* Dose calculation based on BSA of 1.6 m2.

** Dose calculation based on 60-year old a woman of 65 kg and creatinine clearance in the normal range

4.5 Other Relevant Literature and Data

In a series of 262 EOC eligible patients, in which 45% were platinum-resistant recurrent ovarian cancer, a univariate analysis demonstrated that patients who were treated with an assay-sensitive regimen had an improvement in both PFS and OS compared to patients who were treated with assay-resistant regimens (**Table 4.5.1**) (40). This significant (≥50%) improvement in both PFS and OS represents an OS increase of 14 months. Importantly, these observed improvements in PFS and OS were evident in both the platinum-sensitive and -resistant subgroups.

Table 4.5.1. Multivariate analysis of factors affecting PFS and OS

	PFS		OS	
	HR[a] (95% CI)	p value	HR[a] (95% CI)	p value
Age				
Inc. per 10 years	1.07 (0.93–1.23)	0.360	1.03 (0.87–1.22)	0.744
ECOG PS				
1 or 2 vs. 0	1.12 (0.80–1.57)	0.437	1.30 (0.86–1.95)	0.209
Histology				
Serous vs. others	1.36 (0.96–1.93)	0.082	1.09 (0.73–1.63)	0.684
Tumor grade				
3 vs. 0 or 1	1.13 (0.78–1.63)	0.530	1.20 (0.75–1.90)	0.444
Platinum sensitivity status				
Plat sensitive vs. plat resistant	0.64 (0.47–0.87)	0.004	0.66 (0.45–0.96)	0.029
Chemoresponse assay results				
S vs. I or R	0.66 (0.47–0.94)	0.020	0.59 (0.38–0.93)	0.023

[a] Hazard ratio (HR) estimated from proportional hazards model adjusted for covariates.

Specifically, it was found that patients with tumor response defined as sensitive for their clinical treatment demonstrated **significantly improved PFS in comparison to intermediate or resistant results** [median PFS 8.8 months for S vs. 5.9 months for I + R (HR = 0.67, 95% CI = 0.50–0.91, p = 0.009)] (**Figure 4.5.1 A**). There was no difference in PFS between the I and R groups (HR = 0.92, 95% CI = 0.67–1.26, p = 0.591). The association with in vitro response was consistent within platinum-sensitive and platinum-resistant subpopulations as well (HR: 0.71 vs. 0.66, respectively; p = 0.690 for interaction test), showing improved outcomes when using S regimens in either cohort (**Figure 4.5.1**).

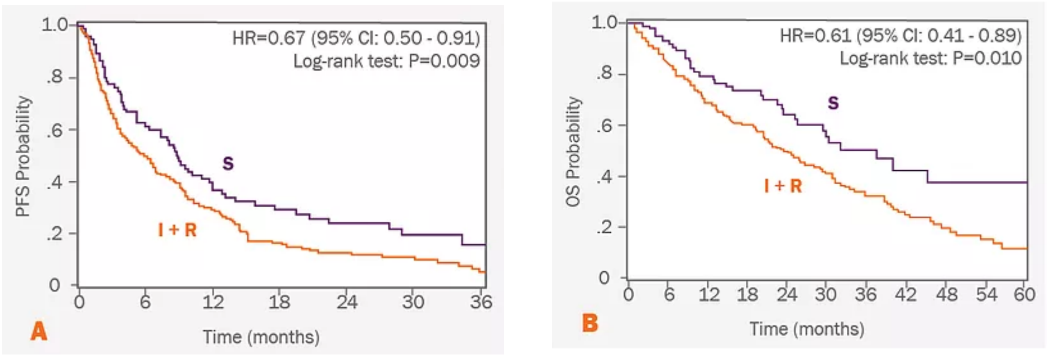

Figure 4.5.1. Clinical outcomes for **A)** progression-free survival (PFS) and **B)** overall-survival (OS) in a prospective, multi-center study of 262 patients with recurrent ovarian cancer who were treated with a therapy that was 'sensitive' according to the chemosensitivity assay.

In multivariate analysis, platinum sensitivity status and in vitro assay results remained the only two independent factors significantly associated with PFS (**Table 4.5.1**). Age did not remain significant in multivariate analysis. Particularly, patients defined as S had a 34% reduced risk of disease progression compared to those defined as I or R, when controlling for age, performance status, histology, tumor grade and prior platinum sensitivity status (HR = 0.66, 95% CI = 0.47–0.94, p = 0.020).

A similar correlation was identified for **OS** (median OS: 37.5 months for S vs. 23.9 months for I + R, HR = 0.61, 95% CI = 0.41–0.89, p = 0.010; **Figure 4.5.1 B**) and the relationship was consistent in multivariate analysis (HR = 0.59, 95% CI = 0.38–0.93, p = 0.023; **Table 4.5.1**).

These improvements amounted to median increases in PFS of 3 months (9 vs. 6 months) and OS of 14 months (38 vs. 24 months). In multivariate analysis, chemosensitivity result was shown to be independently associated with PFS (HR=0.66, 95% CI=0.47-0.94, p=0.020) and OS (HR=0.59, 95% CI=0.38-0.93, p=0.023).

4.6 Compliance Statement

This study will be conducted in full accordance of all applicable Research Policies and Procedures of the investigators institution and all applicable Federal and state laws and regulations including 45 CFR 46, 21 CFR Parts 50, 54, 56, and the Good Clinical Practice: Consolidated Guideline approved by the International Conference on Harmonization (ICH). All episodes of noncompliance will be documented.

The investigators will perform the study in accordance with this protocol, will obtain consent and assent, and will report unanticipated problems involving risks to subjects or others in accordance with the ASRI-WPAHS IRB Policies IRB Policies and Procedures and all federal requirements. Collection, recording, and reporting of data will be accurate and will ensure the privacy, health, and welfare of research subjects during and after the study.

5 STUDY OBJECTIVES

The purpose of this clinical study is to confirm the utility of cancer stem cell assay (ChemoID) tumor testing as a predictor of clinical response in patients with platinum resistant epithelial ovarian, fallopian tube, or primary peritoneal cancer.

Population studied will be female participants experiencing a recurrence of platinum-resistant epithelial ovarian cancer.

Patients will be randomized between a treatment of chemotherapy (standard treatment) or chemotherapy selected by the ChemoID cancer stem cells drug response assay. The ChemoID assay utilizes sample specimens obtained following standard of care biopsy or pleural or peritoneal fluid for the treatment of ovarian cancer to test FDA approved chemotherapies indicated for the disease.

5.1 Primary Objectives (or Aim)

The Primary objective is to compare:

- Objective Response Rate (ORR) in recurrent platinum-resistant EOC patients who have had a treatment with ChemoID-guided treatment compared to control therapy (chemotherapy chosen by the Physician).

5.2 Secondary Objective (or Aim)

The secondary objectives of this study are to compare:

- Progression Free Survival (PFS)
- Duration of Response (DOR)
- CA125 levels
- Health-Related Quality of Life (HRQOL) measured using self-reported, validated questionnaires, addressing physical, psychological, emotional, and social issues.

6 INVESTIGATIONAL PLAN

6.1 General Schema of Study Design

This study is designed as a **parallel group *randomized* controlled clinical trial** to determine if recurrent platinum resistant EOC patients treated with chemotherapy drugs predicted by the ChemoID assay will have better outcomes than patients treated with standard-of-care control therapy (chemotherapy chosen by the Physician).

Accrual sites for this study are:

1. University of Cincinnati
2. University of Oklahoma
3. MD Anderson Cancer Center
4. Duke Medical Center
5. Cleveland Clinic
6. Ohio State University
7. Arizona Oncology
8. University of Miami
9. Arizona Oncology
10. Phoenix-Biltmore Cancer Center
11. Allegheny Health Network Hospitals
12. Edwards Cancer Center, Cabell Huntington Hospital
13. Mississippi Medical Cancer Center Institute

6.1.1 Screening Phase

Potential subjects affected by recurrent platinum resistant epithelial ovarian cancer will be screened using the protocol inclusion and exclusion criteria.

Informed consent will be obtained prior to any study related procedures being performed, including discontinuation of current therapy.

All participants will be studied and treated under standard-of-care procedures. Imaging studies indicated by standard-of-care for EOC patients include cross sectional imaging using CT, PET-CT, MRI, Chest X-Ray, and/or ultrasound.

Participants will be studied by clinical assessment preferably with CT-scans of the chest/abdomen/pelvis pre and post intravenous contrast or without contrast if the patient has a contraindication to CT IV contrast such as severe allergy and/or renal dysfunction.

Blood and urine samples will be collected as per standard-of-care and will be used to confirm eligibility based on clinical laboratory parameters. Blood serum levels of CA125 will be determined at baseline.

Participants will have a urine or serum pregnancy test.

6.1.2 Study Treatment Phase

Visit T1 (Pre-biopsy visit): Eligible patients will be scheduled for tumor biopsy or peritoneal/abdominal fluid collection for diagnosis and for ChemoID assay.

Patients will undergo standard of care biopsy of tumor or drainage of peritoneal or pleural fluid to document recurrence. This sample will be split and sent to Pathology to confirm diagnosis of EOC cells and to the ChemoID lab at the Cabell Huntington Hospital, WV to perform the cancer stem cells drug response assay.

ChemoID assay uses fresh tissue biopsies to determine cytotoxic activity of FDA approved chemotherapies.

Shipment of samples to the ChemoID lab is performed using a secure shipping container for biological samples and the FEDEX carrier using standardized procedure under CLIA and CAP regulations.

Visit T2 (Post-biopsy visit): Pathology report is reviewed. Tamoxifen treatment is started. Study Coordinator calls ChemoID lab to verify that tumors sample provided is viable and growing.

Visit T3 (Randomization visit): Eligible patients are ***randomized*** and enrolled in the study. Study coordinator calls the ChemoID lab to request the release of the ChemoID assay results **only** for patients enrolled in the active arm (ChemoID guide therapy). Tamoxifen treatment is continued.

Visit T4 (ChemoID assay review): Review of the ChemoID drug assay results for participants assigned to assay-guided Arm. Tamoxifen treatment is continued.

Visit T5 (Start of Treatment): Tamoxifen treatment is suspended. Participants are treated with single-agent chemotherapy according to the assigned study arm.

In ARM 1: Participants will be screened by the ChemoID drug response assay; however, their test results will not be released from the lab to the Clinical Investigators until the end of the study and participants will be treated with a single-agent standard-of-care chemotherapy drug (chemotherapy chosen by the Physician from the provided list).

In ARM 2: Participants will be screened by the ChemoID drug response assay and their test results will be released from the lab to the Clinical Investigators and participants will be treated with a ChemoID-guided standard-of-care single-agent chemotherapy drug from the provided list.

6.1.3 Follow-up Phase

Follow-up as indicated by standard-of-care includes cross sectional imaging using CT, PET-CT, MRI, Chest X-Ray, and/or ultrasound. Participants will be followed according to standard-of-care by clinical assessment at the beginning of each cycle of chemotherapy and by imaging assessment every 8 weeks (+/- 7 days) for the first year and every 12 weeks (+/- 7 days) after the first year as per standard of care preferably with CT-scans of the chest/abdomen/pelvis pre and post intravenous contrast or without contrast if the patient has a contraindication to CT IV contrast such as severe allergy and/or renal dysfunction. If indicated, MRI scans pre- and post-intravenous gadolinium contrast will be performed under standard-of-care unless the patient has a contraindication to gadolinium contrast, then non-contrast chest/abdomen/pelvis MRI will be obtained.

Response to chemotherapy will be evaluated according to the Response Evaluation Criteria in Solid Tumors version 1.1. (RECIST) (41). The follow-up phase will continue until disease progression up to 24 months.

6.2 Allocation to Treatment Groups and Blinding

Recurrent epithelial ovarian cancer (EOC) eligible participants will be *randomized* using a statistical random number generator (built in REDCap EDC) and will be assigned to one of the two arms of the study. Study investigators will be kept blind to the schedule.

All participants will be screened by the ChemoID drug response assay. Treating physician will receive the ChemoID assay results **only** for those participants who are randomized to receive ChemoID-guided treatment (ARM2). Physician should treat patients in the ChemoID-guided arm with a drug displaying the highest cell kill on cancer stem cell and bulk of tumor based on ChemoID assay result, compatibly with patient health status.

6.3 Study Duration, Enrollment and Number of Sites

6.3.1 Duration of Study Participation

The study duration per subject will be up to 24 months. Participants will be followed according to standard-of-care every 2 - 3 months intervals (8 weeks +/- 7 days for the first year and every 12 weeks +/- 7 days after the first year) by clinical assessments as detailed in Table 1: schedule of study procedures.

6.3.2 Total Number of Study Sites/Total Number of Subjects Projected

The study will be conducted at 13 investigative sites, all in the United States.

The total number of evaluable subjects projected is N=220 (see statistical section below).

6.4 Study Population

6.4.1 Inclusion Criteria

1. Informed consent obtained and signed.
2. Participant is willing and able to commit to study procedures including long-term follow-up visit(s);
3. Participant must be a female and at least 18 years of age at the time of enrollment.
4. Negative pregnancy test for women of childbearing potential.
5. Participant has been diagnosed with recurrent platinum resistant epithelial ovarian, peritoneal, or fallopian tube carcinoma.

6. Participant must have measurable disease by imaging or objective physical parameter.
7. Participant has agreed to provide a core biopsy of the primary site, a secondary metastatic site, or a paracentesis or thoracentesis for fluid collection.
8. An adequate fresh sample can be provided to be submitted for ChemoID testing.
9. Participant has disease of one of the following histologic epithelial cell types: high-grade serous adenocarcinoma, endometrioid adenocarcinoma, undifferentiated carcinoma, transitional cell carcinoma, clear cell carcinoma, or adenocarcinoma, not otherwise specified (N.O.S.). Cytologic confirmation of diagnosis is acceptable for participants treated with neoadjuvant therapy who have not had a surgical procedure for a histologic confirmation. Patients with low-grade serous or mucinous adenocarcinoma are not eligible, nor are patients with pure ovarian sarcomas.
10. Participant has received ≤ 5 prior regimens including at least 1 platinum-based regimen for their ovarian, peritoneal, or fallopian tube carcinoma.
11. Participant must have an estimated life expectancy of greater than six months, as determined by the investigator.
12. Participant requires chemotherapy and the investigator plans to administer one of the regimens of interest as deemed by her physician.
13. Participant must have an ECOG Performance Status Score of ≤ 2, KPS≥70, or 0-1 GOG status
14. Adequate laboratory values within 60 days of enrollment to study defined as follows:
    a. ANC ≥ 1500/mm3
    b. Hgb ≥ 10 mg/dl
    c. Hct ≥ 28%
    d. Platelet count ≥ 100,000/µL
    e. Serum creatinine ≤ 2.0 mg/dl
    f. Total bilirubin ≤ 2.5 mg/dl
    g. AST/SGOT ≤ 3 times ULN. If intrahepatic liver metastases are present, AST and ALT must be ≤ 5 times institutional ULN.

6.4.2 Exclusion Criteria

1. Use of Avastin planned to treat participant.
2. Participant has ovarian stromal, germ cell tumors or pure sarcomas.

3. Participant has borderline carcinoma (uncertain malignant potential) mucinous or low-grade serous carcinoma.
4. Participant is pregnant or lactating.
5. Participants of childbearing potential not employing adequate contraception.
6. Participants who are at risk of failure of compliance to the visit schedules and procedures including those with psychiatric disease that would substantially impact compliance
7. Estimated life expectancy of <6 months, as estimated by the investigator in consultation with participating oncologists.
8. Participants with symptomatic cardiac conditions (i.e. NYHA class III/IV or uncompensated angina).
9. Enrollment in another clinical study that precludes allowing the oncologist to select chemotherapy regimens.
10. Previously participated in this study.
11. Any condition that would, in the opinion of the investigator, place the participant at an unacceptable risk, or render the participant unable to meet the requirements of the protocol (including long-term study follow-up).
12. CA-125 only disease without RECIST 1.1 measurable or otherwise evaluable disease.
13. Participant may not use any complementary or alternative medicines including natural herbal products or folk remedies as they may interfere with the effectiveness of the study treatments.

Participants who do not meet all of the enrollment criteria may not be enrolled. Any violations of these criteria must be reported in accordance with IRB Policies and Procedures.

6.5 Requirements for Study Entry, Treatment, and Follow-Up

6.5.1 Pre-Treatment Assessment

The following observations and tests are to be performed and recorded on the appropriate form(s). Please note: Each entry refers to the corresponding footnote at the end of the table.

Assessments	Prior to Enrollment (calendar days) *or as per institutional standard of care	Prior to Cycle 1 Day 1 Treatment (calendar days) *or as per institutional standard of care
Informed Consent	≤ 28 days	
History and Physical	≤ 28 days	≤ 7 days
Vital Signs (Blood Pressure, Heart Rate, Temperature and Pulse Oxygen Saturation)	≤ 28 days	Day of treatment
Height	≤ 28 days	≤ 7 days
Weight	≤ 28 days	≤ 7 days
Performance Status (ECOG)	≤ 28 days	≤ 7 days
Toxicity Assessment	≤ 14 days	≤ 7 days
Patient Reported Outcomes Assessment		≤ 7 days
Concurrent Medications	≤ 14 days	Day of treatment
CBC/Differential/Platelets	≤ 14 days	≤ 7 days
Chemistries (including Sodium, Potassium, Chloride, bicarbonate, Calcium, Glucose, BUN/Creatinine, Total Bilirubin, Total Protein, ALT, AST, Alkaline Phosphatase, Albumin)	≤ 14 days	≤ 7 days
CA125	≤ 14 days	≤ 7 days
Pregnancy Test (for patients of child bearing potential)	≤ 14 days	≤ 3 days [a]
Chest imaging (X-ray or CT scan of the chest)	≤ 28 days	
Radiographic Tumor Measurement (CT or MRI of the abdomen and pelvis) [b]	≤ 28 days	≤ 28 days
Electrocardiogram	≤ 14 days	≤ 7 days
MUGA or echocardiogram [c]		≤ 28 days

[a] The minimum sensitivity of the pregnancy test must be 25 IU/L or equivalent units of hCG.
[b] Radiographic tumor measurements should be obtained via imaging of at least the chest, abdomen, and pelvis at baseline. If chest imaging at baseline reveals evidence of measurable disease, then subsequent radiographic tumor assessments must also include chest imaging. See RECIST 1.1 for allowable imaging modalities used to assess disease at baseline and subsequent assessments. Contrast CT is the preferred modality.
[c] MUGA or echocardiogram should be performed prior to receiving anthracycline-based therapy. LVEF assessment by MUGA or echocardiogram should be performed on an every 12-week basis thereafter.

6.5.2 **Assessment During Treatment**

The following observations and tests are to be performed and recorded on the appropriate form(s). Please note: Each entry refers to the corresponding footnote at the end of the table.

Parameter	Day 1 of each cycle of therapy	Day 8 & 15 of each cycle *
Medical history and physical examination	≤ 3 day of treatment	
Concomitant medications	≤ 3 day of treatment	
Adverse event assessment	≤ 3 day of treatment	
QOL (HRQOL)	≤ 3 day of treatment	
Vital signs (Blood Pressure, Heart Rate, Temperature and Pulse Oxygen Saturation)	Day of treatment/assessment	< 3 day of treatment
ECOG performance status	≤ 3 day of treatment	
CBC with Differential	≤ 3 days of treatment	< 3 day of treatment
Serum chemistry[1]	≤ 3 days of treatment	< 3 day of treatment
MUGA or echocardiogram	See footnote [2]	
CA-125 measurement	≤ 3 days of treatment	
Tumor assessment	See footnote [3]	
Audiometry	See footnote [4]	

* Weekly – based regimens only.

1: Serum chemistry includes sodium, potassium, chloride, bicarbonate, BUN, creatinine, glucose, albumin, calcium, AST (SGOT), ALT (SGPT), alkaline phosphatase, total bilirubin.

2: MUGA or echocardiogram should be performed at baseline for all patients assigned to anthracycline-based therapy. LVEF assessment by MUGA or echocardiogram should be performed on an every 12 week basis thereafter.

3: **Tumor reassessment will be time-based, and not cycle-based, with CT scan or MRI performed once every 8 weeks (+/- 7 days),** for the first year and every 12 weeks (+/- 7 days) after the first year, and at any other time if clinically indicated based on symptoms or physical signs suggestive of progressive disease. Imaging assessments can be discontinued if disease progression is confirmed according to RECIST 1.1. However, if a patient discontinues study treatment for any reason other than progression, imaging studies should continue every 8 weeks (+/- 7 days) until progression. Utilize same imaging modality of abdomen and pelvis +/- chest (for patients with evidence of measurable disease on baseline chest imaging; then subsequent radiographic tumor assessments must also include chest imaging.

4: Audiometry should be performed at baseline and periodically for all patients assigned to cisplatin-based therapy.

7 TREATMENT PLAN AND ENTRY/RANDOMIZATION PROCEDURE

7.1 Treatment plan

Patients will be randomized to a standard treatment arm (**Arm 1**) with chemotherapy chosen by the Physician from the provided list, or to a study arm (**Arm 2**) of FDA-approved chemotherapy selected by the ChemoID drug response assay from the provided list. Treatment will take place in the inpatient or outpatient setting depending on the treatment regimen.

Routine clinical assessments including history, physical exam, and lab assessments prior to treatment with chemotherapy should be performed based on standard of care (i.e. assessment prior to Day 1 of chemotherapy cycle) and as often as deemed necessary per treating physician discretion. Reporting of these routine clinical assessments is not necessary unless there are delays in treatment or dose modifications. These visits should be recorded as an Unexpected Visit.

The use of Avastin is not allowed during this trial.

7.2 Duration of therapy

All participants will continue treatment defined as below or consent withdrawal. The patient may voluntarily withdraw from the study at any time.

Patients should continue chemotherapy until there is a documented progression, or unacceptable toxicity.

Use of Avastin is not allowed during this trial.

Patients with Complete Response (defined as normal CA125 and no measurable disease by RECIST) should remain on chemotherapy selected per protocol.

The number of chemotherapy cycles will be left up to the treating physician's discretion. However, patients should receive a minimum of 4 cycles of chemotherapy and ideally treated until a documented Partial Response and Stable Disease (defined by RECIST 1.1).

In the absence of treatment delays due to adverse event(s), treatment may continue as specified in the above treatment modality sections or until one of the following criteria applies:

- ☐ Disease progression,
- ☐ Intercurrent illness that prevents further administration of treatment,
- ☐ Unacceptable adverse event(s), as described in Section 8
- ☐ Patient decides to withdraw consent for participation in the study, or
- ☐ General or specific changes in the patient's condition render the patient unacceptable for further treatment in the judgment of the investigator.

Because of the differences in cycle lengths between the allowed regimens, tumor reassessment will be time-based, and not cycle-based, with CT scan performed as per standard of care once every 8 weeks (+/- 7 days) for the first year and every 12 weeks (+/- 7 days) after the first year, and at any other time if clinically indicated based on

symptoms or physical signs suggestive of progressive disease. Imaging assessments can be discontinued if disease progression is confirmed according to RECIST 1.1. However, if a patient discontinues study treatment for any reason other than progression, imaging studies should continue on the protocol outlined schedule until progression. An Excel tool will be provided to sites to assist in determining imaging dates.

7.3 Treatment window

For 21 or 28-day cycles, a patient will be permitted to have a new cycle of chemotherapy delayed up to 7 days (without this being considered to be a protocol violation) for *major life events* (e.g., serious illness in a family member, major holiday, vacation which is unable to be scheduled).

It will be acceptable for individual chemotherapy doses to be delivered within a "24 hour window before and after the protocol-defined date" for "Day 1" treatment of weekly treatment or 21- or 28-day cycles. If the treatment due date is a Friday, and the patient cannot be treated on that Friday, then the window for treatment would include the Thursday (1 day earlier than due) through Monday (day 3 past due).

Chemotherapy dose reductions are allowed as per standard clinical practice.

7.4 ARM 1- Physicians Choice of chemotherapy regimens

Patients randomized to the Physician choice chemotherapy may be treated with one of the seven regimens specified in this section per investigator discretion (the planned regimen will be specified after randomization). The number of cycles of therapy should be administered as clinically appropriate and as defined in section 7.2.

Patients must continue treatment until disease progression, defined as objective findings on imaging studies, and **not** progression of disease by CA125 alone.

Regimen 1:

- Pegylated liposomal doxorubicin 40 mg/m^2 IV over approximately 60 minutes on day 1, every 28 days

Regimen 2:

- Docetaxel 75mg/m^2 IV infused over 60 minutes, every 21 days.

Regimen 3:

- Paclitaxel 80 mg/m^2 intravenously (IV) over approximately 60 minutes on days 1, 8, 15, every 28 days.

Regimen 4:

- Carboplatin (AUC 5) IV infused over 30-60 minutes on day 1, every 21 days

Regimen 5:

- Cisplatin 60mg/m^2. Infused at a rate of 1mg/min on day 1, every 21 days.

Regimen 6

- Gemcitabine 800-1000mg/m^2 IV infused over 30 minutes on days 1 and 8, every 21 days

Regimen 7:

- Topotecan 1.25 mg/m^2 IV over approximately 30 minutes on days 1 to 5, every 21 days

Regimen 8:

- Gemcitabine (800-1000mg/m^2) IV infused over 30 minutes on days 1 and 8 every 21 days
- Carboplatin (AUC4) IV infused over 30-60 minutes on day 1 every 21 days

Regimen 9:

- Gemcitabine (800-1000mg/m^2) IV infused over 30 minutes on days 1 and 15 every 28 days
- Cisplatin (50mg/m^2) IV over 30 minutes every 28 days

Regimen 10:

- Pegylated liposomal doxorubicin (30mg/m2) IV on day 1 per institutional standard every 28 days.
- Carboplatin (AUC 5) IV infused over 30-60 minutes on day 1 every 28 days

Regimen 11:

- Paclitaxel (175mg/m^2) IV infused over 3 hours on day 1 every 21 days
- Carboplatin (AUC 5) IV infused over 30-60 minutes on day 1 every 21 days

Regimen 12:

- Docetaxel (75 mg/m^2) IV infused over 60 minutes every 21 days
- Carboplatin (AUC 4) IV over 60 minutes every 21 days

Regimen 13:

- Paclitaxel (80mg/m^2) IV infused over 3 hours on day 1 every 28 days
- Carboplatin (AUC 5) IV infused over 30-60 minutes on day 1 every 28 days

Pre medications with dexamethasone, diphenhydramine, and famotidine (or other H2 blocker) should be administered prior to infusion per institutional or practice standards. Anti-emetics may be administered per institutional and practice standards.

7.5 ARM 2 – ChemoID Drug Response Assay chemotherapy regimens

The physician will choose treatment regimen based on ChemoID drug response assay results. Ideally, the regimen with the highest percentage cell kill for both CSC and bulk of tumor cells should be used, however the physician has the flexibility to choose the best regimen according to anticipated patient tolerability. The regimens tested by ChemoID drug response assay are the same as the one in the Physician choice arm. The number of cycles of therapy should be administered as clinically appropriate and as defined in section 7.2. **Patients must continue treatment until disease progression**, defined as objective findings on imaging studies, and **not** progression of disease by CA125 alone, assuming they do not discontinue treatment for toxicity or consent withdrawal.

Dosing for patients receiving one of the reference regimens should be recalculated for weight changes ≥10% of baseline.

Regimen 1: A cycle will be 28 days in length, with pegylated liposomal doxorubicin (PLD) and carboplatin infused IV on day 1, as detailed below. Anti-emetics may be administered per institutional and practice standards.

- Pegylated liposomal doxorubicin 40 mg/m^2 IV over approximately 60 minutes on day 1, every 28 days

Regimen 2: A cycle will be 21 days in length, with docetaxel infused IV as below on day 1. Pre medications with dexamethasone, diphenhydramine, and famotidine (or other H2 blocker) should be administered prior to docetaxel infusion per institutional or practice standards. Anti-emetics may be administered per institutional and practice standards. Dexamethasone 8mg PO BID for three days beginning day prior to treatment.

- Docetaxel (75 mg/m^2) IV infused over 60 minutes

Regimen 3: A cycle will be 28 days in length, with paclitaxel infused IV as below on day 1. Pre medications with dexamethasone, diphenhydramine, and famotidine (or other H2 blocker) should be administered prior to paclitaxel infusion per institutional or practice standards. Anti-emetics may be administered per institutional and practice standards.

- Paclitaxel 80 mg/m^2 intravenously (IV) over approximately 60 minutes on days 1, 8, 15, every 28 days

Regimen 4: A cycle will be 21 days in length, with carboplatin infused IV as below on day 1. Pre medications with dexamethasone, diphenhydramine, and famotidine (or other H2 blocker) should be administered prior to infusion per institutional or practice

standards. Anti-emetics may be administered per institutional and practice standards.

- Carboplatin (AUC5) IV infused over 30-60 minutes on day 1, every 21 days

Regimen 5: A cycle will be 21 days in length with Cisplatin infused IV on day 1, as detailed Below. Anti-emetics may be administered per institutional and practice standards.

- Cisplatin 60mg/m2 IV. Infuse at a rate of 1mg/min
- Prehydration 1000cc Normal saline bolus over 30 minutes
- Posthydration Mannitol 25 grams in 1000cc Normal Saline IV over 30 minutes

Regimen 6: A cycle will be 21 days in length, with gemcitabine infused IV on days 1 and 8, as detailed below. Anti-emetics may be administered per institutional and practice standards.

- Gemcitabine (800-1000mg/m^2) IV infused over 30 minutes on days 1 and 8, every 21 days

Regimen 7: A cycle will be 21 days in length, with Topotecan infused IV as below on day 1.

- Topotecan 1.25 mg/m^2 IV over approximately 30 minutes on days 1 to 5, every 21 days

Regimen 8: A cycle will be 21 days in length, with gemcitabine infused IV on days 1 and 8 and carboplatin on day 1, as detailed below. Gemcitabine infusion should occur prior to carboplatin infusion on day 1. Anti-emetics may be administered per institutional and practice standards.

- Gemcitabine (800-1000mg/m^2) IV infused over 30 minutes on days 1 and 8
- Carboplatin (AUC4) IV infused over 30-60 minutes on day 1

Regimen 9: A cycle will be 28 days in length with gemcitabine infused IV on days 1 and 15 and cisplatin on Day 1 and Day 15 as detailed below. Gemcitabine infusion should occur prior to carboplatin infusion. Anti-emetics may be administered per institutional and practice standards.

- Gemcitabine (800-1000mg/m^2) IV infused over 30 minutes on days 1 and 15
- Cisplatin (50mg/m^2) IV over 30 minutes
- Prehydration 500cc Normal saline bolus over 30 minutes;
- Posthydration Mannitol 12.5 grams in 500cc Normal Saline IV over 30 minutes;

Regimen 10: A cycle will be 28 days in length, with pegylated liposomal doxorubicin (PLD) and carboplatin infused IV on day 1, as detailed below. PLD infusion should occur prior to carboplatin infusion. Anti-emetics may be administered per institutional and practice standards.

- Pegylated liposomal doxorubicin (30mg/m2) IV on day 1 per institutional standard.
- Carboplatin (AUC 5) IV infused over 30-60 minutes on day 1

Regimen 11: A cycle will be 21 days in length, with paclitaxel and carboplatin infused IV as below on day 1. Paclitaxel infusion should occur prior to carboplatin infusion. Pre medications with dexamethasone, diphenhydramine, and famotidine (or other H2 blocker) should be administered prior to paclitaxel infusion per institutional or practice standards. Anti-emetics may be administered per institutional and practice standards.

- Paclitaxel (175mg/m^2) IV infused over 3 hours on day 1
- Carboplatin (AUC 5) IV infused over 30-60 minutes on day 1

Regimen 12: A cycle will be 21 days in length, with docetaxel and carboplatin infused IV as below on day 1. Docetaxel infusion should occur prior to carboplatin infusion. Premedications with dexamethasone, diphenhydramine, and famotidine (or other H2 blocker) should be administered prior to docetaxel infusion per institutional or practice standards. Anti-emetics may be administered per institutional and practice standards. Dexamethasone 8mg PO BID for three days beginning day prior to treatment.

- Docetaxel (75 mg/m^2) IV infused over 60 minutes
- Carboplatin (AUC 4) IV over 60 minutes

Regimen 13: A cycle will be 21 days in length, with paclitaxel and carboplatin infused IV as below on day 1. Paclitaxel infusion should occur prior to carboplatin infusion. Pre medications with dexamethasone, diphenhydramine, and famotidine (or other H2 blocker) should be administered prior to paclitaxel infusion per institutional or practice standards. Anti-emetics may be administered per institutional and practice standards.

- Paclitaxel (80mg/m^2) IV infused over 3 hours on day 1
- Carboplatin (AUC 5) IV infused over 30-60 minutes on day 1

Pre medications with dexamethasone, diphenhydramine, and famotidine (or other H2 blocker) should be administered prior to infusion per institutional or practice standards. Anti-emetics may be administered per institutional and practice standards.

7.6 Duration of Study

The number of cycles will be per the investigator's discretion. All patients will remain on study receiving treatment until disease progression, unacceptable toxicity, or withdrawal of consent. The patient may voluntarily withdraw from the study at any time.

7.7 Follow-up after study treatment discontinuation

The median PFS reported in studies with platinum-resistant patients treated with chemotherapy alone is 3.4 months (35-38). All of the agents demonstrated to have similar

response rates (10%–15%), PFS (3–4 months), and OS (~12 months). Choice of which agent to use is usually based on toxicity profile, the previous toxicities experienced by the patient, and patient preference (3). In this trial the primary analysis of objective response rate (ORR) will be a stratified log-rank test and intention-to-treat (ITT) with patients analyzed according to the arm to which they were randomized.

8 TREATMENT MODIFICATIONS/MANAGEMENT

Toxicity assessments will be done using NCI Common Terminology Criteria for Adverse Events (CTCAE v5.0). CTCAE v5.0 is identified and located on the CTEP website at (http://ctep.cancer.gov/protocolDevelopment/electronic_applications/ctc.htm).

For adverse events (AEs) that are unrelated to the study drugs, study treatment may be held for up to 14 days at the discretion of the treating investigator. Drug holds of greater than 14 days for unrelated AEs where the patient is experiencing ongoing clinical benefit may be considered after discussion with the Study Chair.

All AEs experienced by participants will be collected from the time of the first dose of study treatment, through the study and until the final study visit. Participants continuing to experience toxicity at the off study visit may be contacted for additional assessments until the toxicity has resolved or is deemed irreversible.

There will be no dose escalations or re-escalations on this study.

Dose delays and modifications will be made using the following recommendations.

Use of Avastin is not allowed during this trial.

8.1 Hematologic Toxicity

Myeloid growth factors (filgrastim or pegfilgrastim) may be used per institutional standards. It is recommended that NCCN and/or ASCO guidelines be consulted. Filgrastim should be administered subcutaneously starting 24 to 72 hours after the last dose of chemotherapy and continuing through hematopoietic recovery, but should not be administered within the 48 hours preceding the next dose of cytotoxic chemotherapy. Pegfilgrastim should be administered at 6mg subcutaneously 24 to 72 hours after the last dose of chemotherapy and should not be administered within 2 weeks preceding the next dose of cytotoxic chemotherapy. Pegfilgrastim should not be used for patients receiving chemotherapy that is given less than every 2 weeks.

Use erythropoietin (EPO) per standard of care National Comprehensive Cancer Network (NCCN) and/or institutional guidelines, iron supplements, and/or transfusions as clinically indicated for management of anemia. Dose modifications or holding chemotherapy for anemia should not be done.

Treating physicians should be aware of the recent changes in prescribing information for the erythropoiesis stimulating agents (including Aranesp, Epogen and Procrit) which note

that there is a potential risk of shortening the time to tumor progression or disease-free survival, and that these agents are administered only to avoid red blood cell transfusions. They do not alleviate fatigue or increase energy. They should not be used in patients with uncontrolled hypertension. They can cause an increased incidence of thrombotic events in cancer patients on chemotherapy. The updated package inserts should be consulted. Transfusions may be administered as clinically indicated for management of anemia, but no dose modifications or chemotherapy withholding is necessary. Patients will NOT receive prophylactic thrombopoietic agents. Patients may NOT receive amifostine.
Initial treatment modifications will consist of cycle delay and/or dose reduction as indicated below for each regimen. **All arms require ANC of 1500/mm^3 and platelets of 100,000/mm^3 for treatment initiation, the start of each cycle (Day #1), and treatment resumption after holding treatment.**

8.1.1.1 ***Regimen 1: Pegylated Liposomal Doxorubicin (PLD) (40 mg/m2)***

Dose reductions for next course will be performed according to following guidelines for nadir:

**Table 8.1.1.1 A: Dose modifications for neutropenia, PLD, Regimen 1**		
	Hematologic Event	Dose Modification
Occurrence	500–< 1,500/ mm^3	Wait until ANC ≥ 1,500/ mm^3; redose with no dose reduction
Initial Occurrence	Grade 4 neutropenia (< 500/mm^3)	Wait until ANC ≥ 1,500/ mm^3; reduce PLD dose to 30 mg/m^2
Second Occurrence	Grade 4 neutropenia (< 500/mm^3)	Wait until ANC ≥ 1,500/ mm^3; reduce PLD dose to 20 mg/m^2
Third occurrence	Grade 4 neutropenia (< 500/mm)	Discontinue PLD

**Table 8.1.1.1 B: Dose modifications for thrombocytopenia, PLD, Regimen 1**		
	Hematologic Event	Dose Modification
Occurrence	25,000–< 75,000/ mm^3	Wait until platelets ≥ 100,000/ mm^3; redose with no dose reduction
Initial Occurrence	Any occurrence of grade 4 thrombocytopenia (platelets < 25,000/mcL) or Severe bleeding	Wait until platelets ≥ 100,000/ mm^3; reduce PLD dose to 30 mg/m^2
Second Occurrence	If any of the above toxicities occur after initial dose reduction	Wait until platelets ≥ 100,000/ mm^3; reduce PLD dose to 20 mg/m^2
Third occurrence	If any of the above toxicities occur after initial dose reduction	Discontinue PLD

The WBC count must be ≥ 3000/mm^3, ANC ≥ 1500/mm^3 and the platelet count ≥ 100,000/mm^3 prior to the beginning of the following course of treatment on day 1.

For patients who do not achieve hematological recovery on scheduled day of the course, complete blood counts should be performed twice weekly until the above defined limits are achieved. If hematological recovery is achieved within 14 days after the scheduled day of the course, the full dose of PLD adjusted for the previous nadir should be administered immediately. If hematological recovery is not achieved 14 days or more after the scheduled day of the course, the patient will discontinue treatment.

No more than two dose-reductions because of postponed treatment are permitted.

8.1.1.2 *Regimen 2: Docetaxel (75mg/m2)*

Day 1 of any given cycle of Regimen 2 should not be administered unless the ANC is ≥ 1,500/mcL and the platelet count is ≥ 100,000/mcL. Treatment may be delayed for a maximum of 3 weeks until these parameters are met. If patient counts fail to recover adequately within three weeks, discontinue protocol directed therapy. Dose modifications may occur for neutropenic or thrombocytopenic events, as detailed in the following tables. Dose modifications should be carried through to future cycles of therapy once performed.

**Table 8.1.1.2 A: Dose modifications for neutropenia, Regimen 2**		
	Hematologic Event	Dose Modification
Initial Occurrence	Febrile neutropenia† Grade 4 neutropenia lasting ≥ 7 days ANC < 1000/mcL on Day 1 Treatment delay > 7 days for neutropenia	Reduce docetaxel dose to 45mg/m2
Second Occurrence	If any of the above toxicities occur after initial dose reduction	Reduce docetaxel dose to 30mg/m2
Third Occurrence	If any of the above toxicities occur after two dose reductions	Discontinue docetaxel
† Febrile neutropenia is defined within the CTCAE as a disorder characterized by an ANC <1000/mcL and a single temperature of >101 degrees F or a sustained degree of ≥ 100.4 degrees F for more than an hour.		

**Table 8.1.1.2 B: Dose modifications for thrombocytopenia, Regimen 2**		
	Hematologic Event	Dose Modification
Second Occurrence	If any of the above toxicities occur after initial dose reduction	Reduce docetaxel dose to 45mg/m2
Third Occurrence	If any of the above toxicities occur after two dose reductions	Discontinue docetaxel

† At the discretion of the investigator, a dose reduction may be performed for platelet counts < 100,000/mcL on Day 1.

8.1.1.3 ***Regimen 3: Weekly Paclitaxel (80mg/m2)***

Dose reductions for next course will be performed according to following guidelines for nadir:

**Table 8.1.1.3 A: Dose Modification for neutropenia, <u>Weekly Paclitaxel, Regimen 3</u>**		
Initial Occurrence	Febrile Neutropenia<br>Grade 4 neutropenia (< 500/mm^3) lasting ≥7 days<br>ANC < 1000/mm3 on Day 1<br>Treatment delays > 7 days for neutropenia	Reduce paclitaxel dose to 70 mg/m
Second Occurrence	If any of the above toxicities occur after initial dose reduction	Reduce paclitaxel dose to 60 mg/m2††
Third Occurrence	If any of the above toxicities occur after two dose reductions	Discontinue paclitaxel
† Febrile neutropenia is defined within the CTCAE as a disorder characterized by an ANC <1000/mm3 and a single temperature of >101 degrees F or a sustained degree of ≥ 100.4 degrees F for more than an hour.<br>††Up to 2 dose reductions (70 mg/m^2 and 60 mg/m^2) are acceptable for paclitaxel. Instead of a second dose reduction, 70 mg/m^2 can be maintained if subsequently a one weekly dose is omitted within one cycle. For selected patients, after discussion with Study Chair, a third dose reduction in paclitaxel to 60 mg/m^2 days #1, #8, and #15 can be considered.		

**Table 8.1.1.3 B: Dose Modification for thrombocytopenia, <u>Weekly Paclitaxel, Regimen 3</u>**		
	<u>Hematologic Event</u>	<u>Dose Modification</u>
Initial Occurrence	Any occurrence of grade 4 thrombocytopenia (platelets < 25,000/ mm^3)<br>Grade 3 thrombocytopenia with bleeding event (platelets 25,000 to <50,000/ mm^3) - Severe Bleeding	Reduce paclitaxel dose to 70 mg/m^2
Any Occurrence	Severe Bleeding associated with Febrile Neutropenia	Discontinue paclitaxel
Second Occurrence	If any of the above toxicities occur after initial dose reduction	Reduce paclitaxel dose to 60 mg/m^2†

Third Occurrence	If any of the above toxicities occur after two dose reductions	Discontinue paclitaxel
†Up to 2 dose reductions (70 mg/m^2 and 60 mg/m^2) are acceptable for paclitaxel. Instead of a second dose reduction, 70 mg/m^2 can be maintained if subsequently a one weekly dose is omitted within one cycle. For selected patients, after discussion with Study Chair, a third dose reduction in paclitaxel to 60 mg/m^2 days #1, #8, and #15 can be considered.		

The WBC count must be ≥ 3000/mm3, ANC ≥ 1500/mm3 and the platelet count ≥ 100,000/mm3 prior to the beginning of the following course of treatment on day 1.

The day 8, 15, and 22 paclitaxel dose will not be given unless the ANC is at least 500/mm3 and the platelet count is as least 50,000/mm3. If not given, these doses are omitted and not made up.

For patients who do not achieve hematological recovery on scheduled day of the course, complete blood counts should be performed twice weekly until the limits defined above are achieved. If hematological recovery is achieved within 14 days after the scheduled day of the course, the full dose of paclitaxel adjusted for the previous nadir should be administered immediately. If hematological recovery is not achieved 14 days or more after the scheduled day of the course, the patient will discontinue treatment.

No more than two dose-reductions because of postponed treatment are permitted. However, instead of a second paclitaxel dose reduction, 70 mg/m^2 can be maintained if subsequently a one weekly dose is omitted within one cycle. For selected patients, after discussion with Study Chair, a third dose reduction in paclitaxel to 60 mg/m^2 days #1, #8, and #15 can be considered.

8.1.1.4 ***Regimen 4: Carboplatin (AUC5)***

Day 1 of any given cycle of Regimen 4 should not be administered unless the ANC is ≥ 1,500/mcL and the platelet count is ≥ 100,000/mcL. Treatment may be delayed for a maximum of 14 days until these parameters are met. If patient counts fail to recover adequately within three weeks, discontinue protocol directed therapy.

Dose modifications may occur for neutropenic or thrombocytopenic events, as detailed in the following tables. Dose modifications should be carried through to future cycles of therapy once performed.

**Table 8.1.1.4 A: Dose modifications for neutropenia, Regimen 4**		
	Hematologic Event	Dose Modification
Initial Occurrence	Febrile neutropenia†<br>Grade 4 neutropenia lasting ≥ 7 days<br>ANC < 1000/mcL on Day 1<br>Treatment delay > 7 days for neutropenia	Reduce carboplatin by 1 AUC<br>Initiate G-CSF if not already receiving
Second Occurrence	If any of the above toxicities occur after initial dose reduction	Reduce carboplatin by 1 AUC††

Third Occurrence	If any of the above toxicities occur after two dose reductions	Discontinue carboplatin
† Febrile neutropenia is defined within the CTCAE as a disorder characterized by an ANC <1000/mcL and a single temperature of >101 degrees F or a sustained degree of ≥ 100.4 degrees F for more than an hour.<br>††Carboplatin may not be reduced below AUC4.		

**Table 8.1.1.4 B: Dose modifications for thrombocytopenia, Regimen 4**		
	Hematologic Event	Dose Modification
Initial Occurrence	Any occurrence of grade 4 thrombocytopenia (platelets < 25,000/mcL)<br>Grade 3 thrombocytopenia with bleeding event (platelets 25,000 to <50,000/mcL)<br>Platelets < 75,000/mcL on Day 1†<br>Treatment delay > 7 days for thrombocytopenia	Reduce carboplatin by 1 AUC
Second Occurrence	If any of the above toxicities occur after initial dose reduction	Reduce carboplatin by 1 AUC††
Third Occurrence	If any of the above toxicities occur after two dose reductions	Discontinue carboplatin
† At the discretion of the investigator, a dose reduction may be performed for platelet counts < 100,000/mcL on Day 1.<br>††Carboplatin may not be reduced below AUC4.		

8.1.1.5 *Regimen 5: Cisplatin (60mg/m2)*

Day 1 of any given cycle of Regimen 5 should not be administered unless the ANC is ≥ 1,500/mcL and the platelet count is ≥ 100,000/mcL. Treatment may be delayed for a maximum of 3 weeks until these parameters are met. If patient counts fail to recover adequately within three weeks, discontinue protocol directed therapy.

Dose modifications may occur for neutropenic or thrombocytopenic events, as detailed in the following tables. Dose modifications should be carried through to future cycles of therapy once performed.

**Table 8.1.1.5 A: Dose modifications for neutropenia, Regimen 4**		
	Hematologic Event	Dose Modification
Initial Occurrence	Febrile neutropenia†<br>Grade 4 neutropenia lasting ≥ 7 days<br>ANC < 1000/mcL on Day 1<br>Treatment delay > 7 days for neutropenia	Reduce cisplatin to 50mg/m2<br>Initiate G-CSF if not already receiving
Second Occurrence	If any of the above toxicities occur after initial dose reduction	Reduce cisplatin to 40mg/m2

Third Occurrence	If any of the above toxicities occur after two dose reductions	Discontinue cisplatin
† Febrile neutropenia is defined within the CTCAE as a disorder characterized by an ANC <1000/mcL and a single temperature of >101 degrees F or a sustained degree of ≥ 100.4 degrees F for more than an hour.		

Table 8.1.1.5 B: Dose modifications for thrombocytopenia, Regimen 4		
	Hematologic Event	Dose Modification
Initial Occurrence	Any occurrence of grade 4 thrombocytopenia (platelets < 25,000/mcL)<br>Grade 3 thrombocytopenia with bleeding event (platelets 25,000 to <50,000/mcL)<br>Platelets < 75,000/mcL on Day 1†<br>Treatment delay > 7 days for thrombocytopenia	Reduce cisplatin to 50mg/m2
Second Occurrence	If any of the above toxicities occur after initial dose reduction	Reduce cisplatin to 40mg/m2
Third Occurrence	If any of the above toxicities occur after two dose reductions	Discontinue cisplatin
† At the discretion of the investigator, a dose reduction may be performed for platelet counts < 100,000/mcL on Day 1.		

8.1.1.6 ***Regimen 6: Gemcitabine (800-1000 mg/m2)***

The WBC count must be ≥ 3000/mm^3, ANC ≥ 1500/mm^3 and the platelet count ≥ 100,000/mm^3 prior to the beginning of the following course of treatment on day 1.

For patients who do not achieve hematological recovery on scheduled day of the course, complete blood counts should be performed twice weekly until the above defined limits are achieved. If hematological recovery is achieved within 14 days after the scheduled day of the course, the dose of gemcitabine should be adjusted according to the following tables. If hematological recovery is not achieved 14 days or more after the scheduled day of the course, the patient will discontinue treatment.

No more than two dose-reductions because of postponed treatment are permitted.

Table 8.1.1.6 A: Dosing guidelines for hematologic toxicity, Gemcitabine

Treatment Day	ANC (per mcL)		Platelet count (per mcL)	% of full dose
Day 1	≥ 1500<br>< 1500	and<br>or	≥ 100,000<br>< 100,000	100%<br>Delay treatment cycle
Day 8	≥ 1500<br>1000-1499<br>< 1000	and<br>or<br>or	≥ 100,000<br>75,000-99,999<br>< 75,000	100%<br>50%<br>Hold

Table 8.1.2.4 B: Dose modifications for hematologic toxicity, Gemcitabine		
	Hematologic Event	Dose Modification
Initial Occurrence	Febrile neutropenia†<br>ANC < 500/mcL for > 5 days<br>ANC < 100/mcL for > 3 days<br>Grade 4 thrombocytopenia (platelets < 25,000/mcL)<br>Grade 3 thrombocytopenia with bleeding event (platelets 25,000 to <50,000/mcL)<br>Treatment delay > 7 days for neutropenia or thrombocytopenia	Permanently reduce to 800mg/m2 on Days 1 and 8<br>Initiate G-CSF for neutropenic events if not already receiving
Second Occurrence	If any of the above toxicities occur after initial dose reduction	Maintain day 1 dose at 800 and omit day 8 dose.
Third Occurrence	If any of the above toxicities occur after two dose reductions	Discontinue gemcitabine
†Febrile neutropenia is defined within the CTCAE as a disorder characterized by an ANC <1000/mcL and a single temperature of >101 degrees F or a sustained degree of ≥ 100.4 degrees F for more than an hour.		

8.1.1.7 ***Regimen 7: Topotecan (1.25mg/m2)***

The WBC count must be ≥ 3000/mm^3, ANC ≥ 1500/mm^3 and the platelet count ≥ 100,000/mm^3 prior to the beginning of the following course of treatment on day 1. Standard dose-adjustments based on observed toxicities should be made, as follows:

Table 8.1.1.7 A: Dose modifications for neutropenia, Regimen 7 Topotecan		
	Hematologic Event	Dose Modification

Initial Occurrence	Febrile Neutropenia[†]<br>Grade 4 neutropenia (< 500/mm^3) lasting $\geq$7 days<br>Grade 3 neutropenia (500-900/ mm^3) lasting beyond Day 21 of the treatment course or associated with fever or infection	Reduce topotecan dose to 1 mg/m^2
Second Occurrence	If any of the above toxicities occur after initial dose reduction	Reduce topotecan dose to 0.75 mg/m^2
Third Occurrence	If any of the above toxicities occur after two dose reductions	Discontinue topotecan
[†] Febrile neutropenia is defined within the CTCAE as a disorder characterized by an ANC <1000/ mm^3 and a single temperature of >101 degrees F or a sustained degree of ≥ 100.4 degrees F for more than an hour.		

Table 8.1.1.7 B: Dose modifications for thrombocytopenia, Regimen 7 Topotecan		
	Hematologic Event	Dose Modification
Initial Occurrence	Any occurrence of grade 4 thrombocytopenia (platelets < 25,000/ mm^3) or Severe bleeding	Reduce topotecan dose to 1 mg/m^2
Second Occurrence	If any of the above toxicities occur after initial dose reduction	Reduce topotecan dose to 0.75 mg/m^2
Third Occurrence	If any of the above toxicities occur after initial dose reduction	Discontinue topotecan

If the criteria for topotecan administration are not met at the beginning of a new cycle, this cycle should be postponed, but not longer than 14 days, until the patient recovered. If the next treatment cycle is postponed by $\geq$7 days because of toxicity, a dose- reduction by one level applies. Patients who do not have hematologic recovery sufficient for treatment within 14 days should discontinue drug.

8.1.1.8 *Regimen 8: Carboplatin (AUC4) and Gemcitabine (800-1000mg/m2)*

Day 1 of any given cycle of Regimen I should not be administered unless the ANC is ≥ 1,500/mcL and the platelet count is ≥ 100,000/mcL. Treatment may be delayed for a maximum of 3 weeks until these parameters are met. Patients who fail to recover adequate counts within a three-week delay should discontinue protocol-directed cytotoxic therapy. Day 8 treatment should be administered per the guidelines in Table 8.1.1.2.A below, and dose modifications for hematologic events should be performed per Table 8.1.1.2.B.

Table 8.1.1.2 A: Dosing guidelines for hematologic toxicity, Regimen 8				
Treatment Day	ANC (per mcL)		Platelet count (per mcL)	% of full dose

Day 1	≥ 1500<br>< 1500	and<br>or	≥ 100,000<br>< 100,000	100%<br>Delay treatment cycle
Day 8	≥ 1500<br>1000-1499<br>< 1000	and<br>or<br>or	≥ 100,000<br>75,000-99,999<br>< 75,000	100%<br>50%<br>Hold

Table 8.1.1.2 B: Dose modifications for hematologic toxicity, Regimen 8

	Hematologic Event	Dose Modification
Initial Occurrence	Febrile neutropenia[†]<br>ANC < 500/mcL for > 5 days<br>ANC < 100/mcL for > 3 days<br>Grade 4 thrombocytopenia (platelets < 25,000/mcL)<br>Grade 3 thrombocytopenia with bleeding event (platelets 25,000 to <50,000/mcL)<br>Treatment delay > 7 days for neutropenia or thrombocytopenia	Permanently reduce gemcitabine to 800mg/m2 on Days 1 and 8<br>Initiate G-CSF for neutropenic events if not already receiving
Second Occurrence	If any of the above toxicities occur after initial dose reduction	Permanently reduce gemcitabine dose to 800mg/m2 on Day 1 only (Day 8 gemcitabine is permanently omitted)
Third occurrence	If any of the above toxicities occur after two dose reductions	Discontinue gemcitabine and carboplatin

[†]Febrile neutropenia is defined within the CTCAE as a disorder characterized by an ANC <1000/mcL and a single temperature of >101 degrees F or a sustained degree of ≥ 100.4 degrees F for more than an hour.

8.1.1.9 ***Regimen 9: Gemcitabine (800-1000mg/m2) and Cisplatin (50mg/m2)***

Day 1 of any given cycle of Regimen I should not be administered unless the ANC is ≥ 1,500/mcL and the platelet count is ≥ 100,000/mcL. Treatment may be delayed for a maximum of 3 weeks until these parameters are met. Patients who fail to recover adequate counts within a three-week delay should discontinue protocol. Day 15 treatment should be administered per the guidelines in Table 8.1.3.2A.A below, and dose modifications for hematologic events should be performed per Table 8.1.3.2B.

Table 8.1.3.2 A: Dosing guidelines for hematologic toxicity, Regimen 9

Treatment Day	ANC (per mcL)		Platelet count (per mcL)	% of full dose
Day 1	≥ 1500	and	≥ 100,000	100%
	< 1500	or	< 100,000	Delay treatment cycle
Day 15	≥ 1500	and	≥ 100,000	100%
	1000-1499	or	75,000-99,999	50%
	< 1000	or	< 75,000	Hold

Table 8.1.3.2 B: Dose modifications for hematologic toxicity, Regimen 9

	Hematologic Event	Dose Modification
Initial Occurrence	Febrile neutropenia†<br>ANC < 500/mcL for > 5 days<br>ANC < 100/mcL for > 3 days<br>Grade 4 thrombocytopenia (platelets < 25,000/mcL)<br>Grade 3 thrombocytopenia with bleeding event (platelets 25,000 to <50,000/mcL)<br>Treatment delay > 7 days for neutropenia or thrombocytopenia	Permanently reduce gemcitabine to 600mg/m2 on Days 1 and 15<br>Initiate G-CSF for neutropenic events if not already receiving
Second Occurrence	If any of the above toxicities occur after initial dose reduction	Permanently reduce gemcitabine dose to 600mg/m2 on Day 1 only (Day 15 gemcitabine is permanently omitted)
Third Occurrence	If any of the above toxicities occur after two dose reductions	Discontinue gemcitabine and cisplatin

†Febrile neutropenia is defined within the CTCAE as a disorder characterized by an ANC <1000/mcL and a single temperature of >101 degrees F or a sustained degree of ≥ 100.4 degrees F for more than an hour.

8.1.1.10 ***Regimen 10: Carboplatin (AUC5) and Pegylated Liposomal Doxorubicin (PLD) (30mg/m2)***

Day 1 of any given cycle of Regimen 3 should not be administered unless the ANC is ≥ 1,500/mcL and the platelet count is ≥ 100,000/mcL. Treatment may be delayed for a maximum of 3 weeks until these parameters are met. Patients who fail to recover adequate counts within a three-week delay should discontinue protocol-directed cytotoxic therapy.

Dose modifications may occur for neutropenic or thrombocytopenic events, as detailed in the following tables. Dose modifications should be carried through to future cycles of therapy once performed.

Table 8.1.1.3 A: Dose modifications for neutropenia, Regimen 10

	Hematologic Event	Dose Modification
Initial Occurrence	Febrile neutropenia[†]<br>Grade 4 neutropenia lasting ≥ 7 days<br>ANC < 1000/mcL on Day 1<br>Treatment delay > 7 days for neutropenia	Reduce carboplatin by 1 AUC<br>Initiate G-CSF if not already receiving
Second Occurrence	If any of the above toxicities occur after initial dose reduction	Reduce PLD dose to 25mg/m2
Third Occurrence	If any of the above toxicities occur after two dose reductions	Discontinue carboplatin and PLD

[†] Febrile neutropenia is defined within the CTCAE as a disorder characterized by an ANC <1000/mcL and a single temperature of >101 degrees F or a sustained degree of ≥ 100.4 degrees F for more than an hour.

Table 8.1.1.3 B: Dose modifications for thrombocytopenia, Regimen 10

	Hematologic Event	Dose Modification
Initial Occurrence	Grade 4 thrombocytopenia<br>(platelets < 25,000/mcL)<br>Grade 3 thrombocytopenia with bleeding event (platelets 25,000 to <50,000/mcL)<br>Platelets < 100,000/mcL on Day 1[†]<br>Treatment delay > 7 days for thrombocytopenia	Reduce carboplatin by 1 AUC
Second Occurrence	If any of the above toxicities occur after initial dose reduction	Reduce PLD dose to 25mg/m2
Third Occurrence	If any of the above toxicities occur after two dose reductions	Discontinue carboplatin and PLD

[†] At the discretion of the investigator, a dose reduction may be performed for platelet counts < 100,000/mcL on Day 1.

8.1.1.11 ***Regimen 11: Carboplatin (AUC5) and paclitaxel (175mg/m2)***

Day 1 of any given cycle of Regimen I should not be administered unless the ANC is ≥ 1,500/mcL and the platelet count is ≥ 100,000/mcL. Treatment may be delayed for a maximum of 3 weeks until these parameters are met.

Dose modifications may occur for neutropenic or thrombocytopenic events, as detailed in the following tables. Dose modifications should be carried through to future cycles of therapy once performed.

**Table 8.1.1.1 A: Dose modifications for neutropenia, Regimen 11**		
	Hematologic Event	Dose Modification
Initial Occurrence	Febrile neutropenia[†]<br>Grade 4 neutropenia lasting ≥ 7 days<br>ANC < 1000/mcL on Day 1<br>Treatment delay > 7 days for neutropenia	Reduce carboplatin to AUC 4 and Paclitaxel to 135 mg/m2
Second Occurrence	If any of the above toxicities occur after initial dose reduction	Initiate G-CSF if not already receiving
Third occurrence	If any of the above toxicities occur after two dose reductions	Discontinue carboplatin and paclitaxel
[†] Febrile neutropenia is defined within the CTCAE as a disorder characterized by an ANC <1000/mcL and a single temperature of >101 degrees F or a sustained degree of ≥ 100.4 degrees F for more than an hour.<br>[††]Carboplatin may not be reduced below AUC4. If neutropenic event occurs at a carboplatin AUC of 4, carboplatin dosing should be maintained at AUC4 and paclitaxel dose reduced to 135 mg/m2		

**Table 8.1.1.1 B: Dose modifications for thrombocytopenia, Regimen 11**		
	Hematologic Event	Dose Modification
Initial Occurrence	Any occurrence of grade 4 thrombocytopenia (platelets < 25,000/mcL)<br>Grade 3 thrombocytopenia with bleeding event (platelets 25,000 to <50,000/mcL), petechiae, or requiring platelet transfusion<br>Platelets < 75,000/mcL on Day 1[†]<br>Treatment delay > 7 days for thrombocytopenia	Reduce carboplatin by 1 AUC

Second Occurrence	If any of the above toxicities occur after initial dose reduction	Discontinue carboplatin and paclitaxel
Third occurrence	If any of the above toxicities occur after two dose reductions	
† At the discretion of the investigator, a dose reduction may be performed for platelet counts < 100,000/mcL on Day 1. ††Carboplatin may not be reduced below AUC4. If a thrombocytopenic event occurs at a carboplatin AUC of 4, carboplatin dosing should be maintained at AUC4 and paclitaxel dose reduced to 135mg/m2.		

8.1.1.12 ***Regimen 12: Carboplatin (AUC4) and Docetaxel (75mg/m2)***

Day 1 of any given cycle of Regimen I should not be administered unless the ANC is ≥ 1,500/mcL and the platelet count is ≥ 100,000/mcL. Treatment may be delayed for a maximum of 3 weeks until these parameters are met. If patient counts fail to recover adequately within three weeks, discontinue protocol. Dose modifications may occur for neutropenic or thrombocytopenic events, as detailed in the following tables. Dose modifications should be carried through to future cycles of therapy once performed.

Table 8.1.3.3 A: Dose modifications for neutropenia, Regimen 12

	Hematologic Event	Dose Modification
Initial Occurrence	Febrile neutropenia†<br>Grade 4 neutropenia lasting ≥ 7 days<br>ANC < 1000/mcL on Day 1<br>Treatment delay > 7 days for neutropenia	Reduce docetaxel to 65 mg/m2<br>Initiate G-CSF if not already receiving
Second Occurrence	If any of the above toxicities occur after initial dose reduction	Reduce docetaxel dose to 45mg/m2
Third Occurrence	If any of the above toxicities occur after two dose reductions	Discontinue patient from protocol directed therapy
† Febrile neutropenia is defined within the CTCAE as a disorder characterized by an ANC <1000/mcL and a single temperature of >101 degrees F or a sustained degree of ≥ 100.4 degrees F for more than an hour. ††Carboplatin may not be reduced below AUC4. If neutropenic event occurs at a carboplatin AUC of 4, carboplatin dosing should be maintained at AUC4 and docetaxel dose reduced to 45 mg/m2		

Table 8.1.3.3 B: Dose modifications for thrombocytopenia, Regimen 12

	Hematologic Event	Dose Modification

Initial Occurrence	Any occurrence of grade 4 thrombocytopenia (platelets < 25,000/mcL)<br>Grade 3 thrombocytopenia with bleeding event (platelets 25,000 to <50,000/mcL)<br>Platelets < 75,000/mcL on Day 1† Treatment delay > 7 days for thrombocytopenia	Reduce docetaxel to 65 mg/m2
Second Occurrence	If any of the above toxicities occur after initial dose reduction	Reduce docetaxel dose to 45mg/m2
Third Occurrence	If any of the above toxicities occur after two dose reductions	Discontinue patient from protocol directed therapy
† At the discretion of the investigator, a dose reduction may be performed for platelet counts < 100,000/mcL on Day 1.<br>††Carboplatin may not be reduced below AUC4. If a thrombocytopenic event occurs at a carboplatin AUC of 4, carboplatin dosing should be maintained at AUC4 and docetaxel dose reduced to 45mg/m2.		

8.1.1.13 ***Regimen 13: Carboplatin (AUC5) and paclitaxel (80mg/m2)***

Day 1 of any given cycle of Regimen I should not be administered unless the ANC is ≥ 1,500/mcL and the platelet count is ≥ 100,000/mcL. Treatment may be delayed for a maximum of 3 weeks until these parameters are met.

Dose modifications may occur for neutropenic or thrombocytopenic events, as detailed in the following tables. Dose modifications should be carried through to future cycles of therapy once performed.

**Table 8.1.1.1 A: Dose modifications for neutropenia, Regimen 13**		
	Hematologic Event	Dose Modification
Initial Occurrence	Febrile neutropenia†<br>Grade 4 neutropenia lasting ≥ 7 days<br>ANC < 1000/mcL on Day 1<br>Treatment delay > 7 days for neutropenia	Reduce carboplatin by 1 AUC<br>Initiate G-CSF if not already receiving
Second Occurrence	If any of the above toxicities occur after initial dose reduction	Reduce paclitaxel dose to 60/m2
Third occurrence	If any of the above toxicities occur after two dose reductions	Discontinue carboplatin and paclitaxel
† Febrile neutropenia is defined within the CTCAE as a disorder characterized by an ANC <1000/mcL and a single temperature of >101 degrees F or a sustained degree of ≥ 100.4 degrees F for more than an hour.<br>††Carboplatin may not be reduced below AUC4. If neutropenic event occurs at a carboplatin AUC of 4, carboplatin dosing should be maintained at AUC4 and paclitaxel dose reduced to 60 mg/m2		

Table 8.1.1.1 B: Dose modifications for thrombocytopenia, Regimen 11		
	Hematologic Event	Dose Modification
Initial Occurrence	Any occurrence of grade 4 thrombocytopenia (platelets < 25,000/mcL)<br>Grade 3 thrombocytopenia with bleeding event (platelets 25,000 to <50,000/mcL)<br>Platelets < 75,000/mcL on Day 1[†]<br>Treatment delay > 7 days for thrombocytopenia	Reduce carboplatin by 1 AUC
Second Occurrence	If any of the above toxicities occur after initial dose reduction	Reduce paclitaxel dose to 60mg/m2
Third occurrence	If any of the above toxicities occur after two dose reductions	Discontinue carboplatin and paclitaxel
[†] At the discretion of the investigator, a dose reduction may be performed for platelet counts < 100,000/mcL on Day 1.<br>[††]Carboplatin may not be reduced below AUC4. If a thrombocytopenic event occurs at a carboplatin AUC of 4, carboplatin dosing should be maintained at AUC4 and paclitaxel dose reduced to 60mg/m2.		

8.2 Non-hematologic toxicity

Management of non-hematologic toxicities with an impact on organ function ≥ grade 2 require reduction of one dose level and delay in subsequent therapy until recovery to grade 1. Dose modifications for alopecia, nausea, or constipation are not recommended. If treatment is delayed for greater than 3 weeks due to a drug-related non-hematologic toxicity, the patient may be discontinued from protocol directed therapy after consultation with the Study Chair.

8.2.1 **Dose Reduction Guidelines**

8.2.1.1 ***Platinum-based Regimens***

Table 8.2.1.1: Guidelines for permanent dose reductions, Reference Regimens		
**Drug**	**First reduction**	**Second reduction**
Carboplatin[†]	Decrease AUC by 1	Decrease AUC by 1
Paclitaxel	Decrease dose to 70 mg/m2	Decrease dose to 60 mg/m2
Gemcitabine	Decrease dose to 600mg/m2 on Days 1 and 8	Discontinue Day 8 dosing
Pegylated liposomal doxorubicin	Decrease dose to 25mg/m2	Decrease dose to 20mg/m2
Cisplatin	Decrease dose to 50mg/m2	Decrease dose to 40mg/m2
Docetaxel	Decrease dose to 45mg/m2	Decrease dose to 30mg/m2

†Carboplatin dose should not be reduced below an AUC of 4. Patients requiring a dose reduction below AUC4 should be removed from protocol-directed therapy.

8.2.1.2 ***Non-platinum based regimens***

Table 8.2.1.2: Guidelines for permanent dose reductions, Reference Regimens			
**Drug**	**Initial dose level**	**First reduction**	**Second reduction**
Paclitaxel weekly[1]	80 mg/m^2	Decrease dose to 70 mg/m^2	Decrease dose to 60 $mg/\ m^2$
Pegylated liposomal doxorubicin	40 mg/m^2	Decrease dose to 30 mg/m^2	Decrease dose to 20 $mg/\ m^2$
Topotecan mg/m^2 per day over 5 days	1.25 mg/m^2	Decrease dose to 1 mg/m^2	Decrease dose to 0.75 mg/m^2
Docetaxel	75mg/m^2	Decrease dose to 65mg/m^2	Decrease dose to 55mg/m^2
Paclitaxel	80mg/m^2	Decrease dose to 70mg/m^2	Decrease dose to 60mg/m^2
Gemcitabine	800-1000mg/m^2	Decrease dose to 600mg/m^2	Discontinue Day 8 dosing

[1] Up to 2 dose reductions (70 mg/m^2 and 60 mg/m^2) are acceptable for paclitaxel. Instead of a second dose reduction, 70 mg/m^2 can be maintained if subsequently a one weekly dose is omitted within one cycle. For selected patients, after discussion with Study Chair, a third dose reduction in paclitaxel to 60 mg/m^2 days #1, #8, and #15 can be considered.

8.2.2 **Proteinuria**

Patients should be monitored with random urine protein-to-creatinine ratios on Day 1 of each cycle.

Table 8.2.2: Guidelines for Proteinuria		
Grade	Definition	Recommended treatment and management
Grade 1	0.15 to 1.0 g/24 hr protein as estimated by random urine protein-to-creatinine ratio	Continue chemotherapy
Grade 2	>1.0 to 3.5 g/24hr protein as estimated by random urine protein-to-creatinine ratio	Continue chemotherapy.
Grade 3	≥3.5 g/24 hr protein as estimated by random urine protein-to-creatinine ratio	Continue chemotherapy.
Grade 4	Nephrotic syndrome	The patient is removed from the study.

8.2.3 **Hypertension**

Patients should be monitored prior to each treatment with measurement of blood pressure. Medication classes used for management of patients with Grade 3 hypertension include ACE inhibitors, Beta blockers, diuretics, and calcium channel blockers.

Table 8.2.3: Guidelines for Hypertension

Grade	Definition	Recommended treatment and management
Grade 1	Systolic BP 120-139mmHG or diastolic BP 80-89 mmHg)	Continue chemotherapy without dose modifications
Grade 2	Systolic BP 140-159mm HG or diastolic BP 90-99 mmHg); medical intervention indicated; recurrent or persistent (≥24 hrs); symptomatic increase by >20mmm Hg (diastolic) or to >140/90 mmHg if previously within normal limits; monotherapy indicated	Continue chemotherapy. Start or adjust antihypertensive therapy.
Grade 3	Systolic BP ≥ 160mmHg or diastolic BP ≥100 mmHg); medical intervention indicated; more than one drug or more intensive therapy than previously used indicated	Suspend chemotherapy. If not controlled to 140/90 mm Hg with medication within, patient is to be removed from the study.
Grade 4	Life-threatening consequences (e.g., hypertensive emergency, transient or permanent neurologic deficit, hypertensive crisis); urgent intervention indicated	Patient to be removed from the study.

8.2.4 **Hypersensitivity reactions**

Of note, the occurrence of a hypersensitivity reaction may not be a dose limiting toxicity. Depending on the severity of their hypersensitivity reaction, patients experiencing a hypersensitivity reaction may be retreated at full doses under institutional protocols to prevent hypersensitivity reactions. The below table provides recommendations regarding approach to continued treatment for patients experiencing hypersensitivity reactions. Sites may also follow institutional guidelines for prevention and/or avoidance of hypersensitivity reactions.

Table 8.2.4 A: Guidelines for hypersensitivity reactions

Grade	Definition	Recommended treatment and management
Grade 1	Mild transient reaction; infusion interruption not indicated; intervention not indicated	May consider retreatment per institutional guidelines. May consider pre-medication with steroids, antihistamines (diphenhydramine), or H2 blockers.

Grade 2	Therapy or infusion interruption indicated but responds promptly to symptomatic treatment (e.g., antihistamines, NSAIDs, narcotics, IV fluids); prophylactic medications indicated for <=24 hrs.	Stop infusion immediately. May consider retreatment or desensitization, per institutional guidelines. Patients may be discontinued from protocol directed therapy at the discretion of the investigator.
Grade 3	Prolonged (e.g., not rapidly responsive to symptomatic medication and/or brief interruption of infusion); recurrence of symptoms following initial improvement; hospitalization indicated for clinical sequel	Stop infusion immediately. May consider re-challenge under desensitization protocol, per institutional guidelines. Patients may be discontinued from protocol-directed therapy at the discretion of the investigator.
Grade 4	Life-threatening consequences; urgent intervention indicated.	Do not re-challenge. Protocol directed therapy should be discontinued.

8.2.5 **Modifications for hepatic toxicity**

Dose adjustments should be per institutional practice and guidelines. Criteria for dose holds and modifications on the reference arms for non-hematologic toxicity should follow institutional practice and prescribing information if not specified.

8.2.6 **Modifications for renal toxicity**

In patients receiving cisplatin-based therapy adjustments for renal dysfunction are as follows:

- If creatinine rises to greater than 1.5mg/dL, obtain creatinine clearance (CrCl). Creatinine clearance should be obtained via Crockcroft Gault calculation. Cisplatin dosing will be managed based on creatinine clearance as follows:
  - If CrCl is 50ml/min or greater, continue cisplatin at current dose level.
  - If the CrCl is less than 50ml/min, hold cisplatin. The CrCl should be checked weekly. If the CrCl returns to or surpasses 50ml/min, resume cisplatin with a one dose level reduction.
  - If the CrCl remains under 50ml/min for more than 4 weeks, discontinue cisplatin.

Dose adjustments for non-cisplatin based regimens should be per institutional practice and guidelines. Criteria for dose holds and modifications on the reference arms for non-hematologic toxicity should follow institutional practice and prescribing information if not specified.

8.2.7 **Diarrhea**

Diarrhea is often observed with chemotherapy, and active and early management of diarrhea is recommended even with grade 1 diarrhea. Management as follows:

**Table 8.2.7: Management of Diarrhea**

Toxicity	Management/Modifications
Initial grade 1 or 2 diarrhea:	Patients can take loperamide (per standard practice) and continue to take loperamide until patients are free from diarrhea for at least 12 hours. The dose of loperamide should not exceed 16mg in a 24-hour period. Patients should also be counseled to start a BRAT (bananas, rice, applesauce, toast) diet.
	If diarrhea persists despite 24 hours of loperamide treatment, hold therapy for a maximum of 7 days, continue loperamide, and maintain hydration. Therapy may be restarted at the same dose once patients have been free from diarrhea for 12 hours.
For either persistent grade 2 diarrhea or grade 3 or 4 diarrhea:	Refer to Table 8.2.1.1 and 8.2.1.2.

8.2.8 Decrease in LVEF

Patients who are receiving an anthracycline based treatment should undergo an echocardiogram (ECHO) or multigated acquisition (MUGA) scan at baseline and every 12 weeks while on study:

Table 8.2.8: Management and Monitoring of Decreased LVEF

Relationship of LVEF to Institution's LLN	LVEF Decrease < 10%	LVEF Decrease 10-15%	LVEF Decrease ≥ 16%
Normal	Continue	Continue	Continue and repeat MUGA/ECHO within 1-2 cycles
1-5% below LLN	Continue and repeat MUGA/ECHO within 1-2 cycles	Continue and repeat MUGA/ECHO within 1-2 cycles	HOLD and repeat MUGA/ECHO within 1-2 cycles
≥ 6% below LLN	Continue and repeat MUGA/ECHO within 1-2 cycles	HOLD and repeat MUGA/ECHO within 1-2 cycles	HOLD and repeat MUGA/ECHO within 1-2 cycles

8.2.9 Reversible Posterior Leukoencephalopathy Syndrome (RPLS)

Therapy should be held in patients with symptoms/signs suggestive of RPLS, pending work-up and management, including control of blood pressure. Therapy should be discontinued upon diagnosis of RPLS. Study drugs should not be resumed without consultation with the Study Chair.

After consultation with the Study Chair and the Principal Investigator, consideration of restarting the study may be evaluated in light of any clinical benefit.

8.2.10 **Myelodysplastic Syndrome and Acute Myeloid Leukemia**

Therapy should be held in patients with symptoms/signs suggestive of MDS/AML, pending work-up and management. Therapy should be discontinued indefinitely upon diagnosis of myelodysplastic syndrome or acute leukemia.

8.2.11 **Mucositis and cutaneous toxicity**

In case of grade ≥2 hand-foot syndrome (HFS) or stomatitis, the treatment dose will be delayed until resolved to grade ≤1 or discontinued if not resolved within 2 weeks. In addition, subsequent doses will be reduced if the HFS or stomatitis is grade ≥3. The following tables show the dose modifications and the course delays recommended as a function of the occurrence and severity of mucositis and cutaneous toxicity, respectively.

Table 8.2.11 A: Dose Modification Guidelines for Hand-Foot Syndrome (HFS)

Toxicity Grade	Dose Adjustment
**1:** mild erythema, swelling, or desquamation not interfering with daily activities	**Redose unless patient has experienced previous Grade 3 or 4 HFS.** If so, delay up to 2 weeks and reduce 1 dose level. Return to original dose interval.
**2:** erythema, desquamation, or swelling interfering with, but not precluding normal physical activities; small blisters or ulcerations <2 cm in diameter	**Delay dosing up to 2 weeks or until resolved to Grade 0-1.** If after 2 weeks there is no resolution, therapy should be discontinued. If resolved to Grade 0-1 within 2 weeks and there are no prior Grade 3-4 HFS, continue treatment at previous dose and return to original dose interval. If patient experienced previous Grade 3–4 toxicity, continue treatment with reduce 1 dose level and return to original dose interval.
**3:** blistering, ulceration, or swelling interfering with walking or normal daily activities; cannot wear regular clothing	**Delay dosing up to 2 weeks or until resolved to Grade 0–1.** Reduce 1 dose level and return to original dose interval. If after 2 weeks there is no resolution, therapy should be discontinued.
**4:** diffuse or local process causing infectious complications, or a bed ridden state or hospitalization	**Delay dosing up to 2 weeks or until resolved to Grade 0–1.** Reduce 1 dose level and return to original dose interval. If after 2 weeks there is no resolution, therapy should be discontinued.

Table 8.2.11 B: Dose Modification Guidelines for Stomatitis

Toxicity Grade	Dose Adjustment

**1:** painless ulcers, erythema, or mild soreness	**Redose unless patient has experienced previous Grade 3 or 4 toxicity.** If so, delay up to 2 weeks and reduce 1 dose level. Return to original dose interval
**2:** painful erythema, edema, or ulcers, but can eat	**Delay dosing up to 2 weeks or until resolved to Grade 0-1.** If after 2 weeks there is no resolution, therapy should be discontinued.<br>If resolved to Grade 0-1 within 2 weeks and there are no prior Grade 3-4 stomatitis, continue treatment at previous dose and return to original dose interval. If patient experienced previous Grade 3–4 toxicity, continue treatment with a 1 dose level reduction and return to original dose interval.
**3:** painful erythema, edema, or ulcers, and cannot eat	**Delay dosing up to 2 weeks or until resolved to Grade 0–1.** Reduce 1 dose level and return to original dose interval. If after 2 weeks there is no resolution, therapy should be discontinued.
**4:** requires parenteral or enteral support	**Delay dosing up to 2 weeks or until resolved to Grade 0–1.** Reduce 1 dose level and return to original dose interval. If after 2 weeks there is no resolution, therapy should be discontinued.

8.2.12 Bowel obstruction

Patient is to be removed from the study.

8.2.13 Gastrointestinal perforation or fistula

Patient is to be removed from the study.

9 STUDY PROCEDURES

See Table 1 for the procedures and assessments to be performed during this phase of the study. All screening visit tests and procedures will occur after the informed consent (IC) is signed. No screening exams will take place until the patient is fully informed of the research and signs the informed consent. This visit may take place over more than one day.

9.1 Screening Visit

See Table 1 for the procedures and assessments to be performed during this phase of the study. The Screening Phase will take place according to the study calendar, and it may be split into two visits. The Screening Visit will determine if the patient is eligible for the study.

Following are the steps involved with this phase of the study:

- Physical Exam and Clinical Assessment
- Vital Signs
- Height and Weight
- Pregnancy test
- Laboratory tests
- Prior/Concomitant Medications
- Clinical Laboratory Evaluation
- Chest, abdomen and pelvis CT scan preferentially with contrast and/or anyone or more of the following imaging exams: PET/CT scan, MRI, chest X-Ray, and abdomen ultrasound
- CA-125 serum baseline level
- Review Inclusion/Exclusion criteria
- Informed Consent

9.2 Enrollment Contingency Plans

In order to consider certain unexpected events that can disrupt the planned study schedule, enrolled participants who unexpectedly expire prior to surgery or who are otherwise withdraw from the study after the screening period but before surgery, may be replaced without limit.

9.3 Study Treatment Phase

See Table 1 for the procedures and assessments to be performed during this phase of the study. Eligible participants will be subjected to either tumor biopsy or to peritoneal/pleural fluid collection for diagnosis of recurrence of ovarian carcinoma and for ChemoID (at the same time).

Specimens will be sent to the pathology laboratory for pathological diagnosis and to ChemoID lab for drug response assessment.

Participants will be enrolled and randomized to receive either ChemoID-guided therapy or not. ChemoID drug response assay will test only FDA standard of care cytotoxic drugs indicated to treat recurrent EOC.

Standard of care treatments (physician choice or ChemoID-guided) will be administered to participants. Chemotherapy will be dispensed to participants according to their study arm assignment. Drug compliance and adverse event assessment will be recorded.

Physician will receive ChemoID test results from the ChemoID lab for those patients enrolled in ARM 1 (Physician choice chemotherapy) **only** at the end of the study.

Physician will receive full ChemoID test results from the ChemoID lab **only** for those patients enrolled in ARM 2 (ChemoID guided chemotherapy).

For those patients enrolled in ARM 2 (ChemoID guided chemotherapy) physician will select treatment based on highest cell kill on cancer stem cells and bulk of tumor as indicated by the ChemoID assay results.

Following are the steps involved with this phase of the study:

9.3.1 **Visit T1 (Pre-biopsy visit)**

- Physical Exam
- Vital Signs
- Scheduling for tumor biopsy for pathological diagnosis and for ChemoID drug response assay (at the same time)

  or

- Scheduling for peritoneal of pleural fluid biopsy for pathological diagnosis and for ChemoID drug response assay (at the same time) or
- Laboratory tests
- Better Health-Related Quality of Life (HRQOL) assessment based on questionnaires, addressing physical, psychological, emotional, and social issues.

9.3.2 **Visit T2 (post biopsy visit)**

- Physical Exam (if indicated)
- Vital Signs
- Laboratory tests (if indicated)
- Review of Pathology report
- Tamoxifen treatment is started.
- Study Coordinator calls ChemoID lab to verify that tumors sample provided is viable and growing.

9.3.3 **Visit T3 (*Randomization* visit)**

- Physical Exam (if indicated)
- Vital Signs
- Tamoxifen treatment is continued
- Eligible patients are ***randomized*** and enrolled in the study.
- Study coordinator calls the ChemoID lab to request the release of the ChemoID assay results **only** for patients enrolled in the active arm (ChemoID guide therapy).

9.3.4 **Visit T4**

- Physical Exam (if indicated)
- Vital Signs
- Laboratory tests including CA125 serum level (if indicated)
- Review of ChemoID Assay results (only patients assigned to Arm 2)
- Discussion of treatment plan with patient.
- Tamoxifen treatment continued
- Better Health-Related Quality of Life (HRQOL) assessment based on questionnaires, addressing physical, psychological, emotional, and social issues.

9.3.5 Visit 5 and further study treatment visits (Day 1 of each chemotherapy cycle)

- Physical Exam (if indicated)
- Vital Signs
- Laboratory tests including CA125 serum level (if indicated)
- Tamoxifen treatment is suspended
- Start study intervention by dispensing study drug according to study arms' assignment and their cohorts
- Or continued study interventions by dispensing study drug according to study arms' assignment and their cohorts
- Assess possible adverse events
- Better Health-Related Quality of Life (HRQOL) assessment based on questionnaires, addressing physical, psychological, emotional, and social issues.

9.4 Follow-up Phase

See Table 1 for the procedures and assessments to be performed during this phase of the study.

Participant is assessed at followed-up clinical visits at the beginning of each chemotherapy cycle and every 8 weeks (+/- 7 days) for the first year and every 12 weeks (+/- 7 days) after the first year as per standard of care with radiological imaging (**up to 18 months**) after standard-of-care according to the study arms. Drug compliance and adverse event assessment is also performed.

Outpatient visits should be completed as close to the scheduled visit dates as possible. The visit window is ± 7-14 days from the intended date of the visit. If needed, outpatient visit procedures may be completed over more than one day and, if so, the date of the history and physical exam will be considered the visit date.

Follow-up visits consist of a clinical evaluation as per standard-of-care management of the disease. Laboratory tests of blood counts, glucose level, and blood count, liver function tests are indicated if the participant is receiving chemotherapy, corticosteroids and anti-emetic drugs. CT scans preferably with IV contrast every 8 weeks ± 1 week is standard practice. Other imaging exams indicated are: PET/CT scan, MRI, chest X-Ray, and abdomen ultrasound

Following are the steps involved with this phase of the study:

9.4.1 Visit F1 (8-weeks Clinical and Radiological Imaging follow-up visit)

- Physical Exam
- Vital Signs

- Laboratory tests including serum levels of CA125
- Chest, abdomen and pelvis CT scan with or w/o contrast or other radiological imaging indicated.
- Continued study intervention according to study arms' assignment, if indicated
- Better Health-Related Quality of Life (HRQOL) assessment based on questionnaires, addressing physical, psychological, emotional, and social issues.

9.4.2 **Visit F2 (16-weeks follow-up visit)**

- Physical Exam
- Vital Signs
- Laboratory tests including serum levels of CA125
- Chest, abdomen and pelvis CT scan with or w/o contrast or other radiological imaging indicated.
- Continued study intervention according to study arms' assignment, if indicated
- Better Health-Related Quality of Life (HRQOL) assessment based on questionnaires, addressing physical, psychological, emotional, and social issues.

9.4.3 **Visit F3 (24-weeks follow-up visit)**

- Physical Exam
- Vital Signs
- Laboratory tests including serum levels of CA125
- Chest, abdomen and pelvis CT scan with or w/o contrast or other radiological imaging indicated.
- Continued study intervention according to study arms' assignment, if indicated
- Better Health-Related Quality of Life (HRQOL) assessment based on questionnaires, addressing physical, psychological, emotional, and social issues.

9.4.4 **Visit F4 (32-weeks follow-up visit)**

- Physical Exam

- Vital Signs
- Laboratory tests including serum levels of CA125
- Chest, abdomen and pelvis CT scan with or w/o contrast or other radiological imaging indicated.
- Continued study intervention according to study arms' assignment, if indicated
- Better Health-Related Quality of Life (HRQOL) assessment based on questionnaires, addressing physical, psychological, emotional, and social issues.

9.4.5 **Visit F5 (40-weeks follow-up visit)**

- Physical Exam
- Vital Signs
- Laboratory tests including serum levels of CA125
- Chest, abdomen and pelvis CT scan with or w/o contrast or other radiological imaging indicated.
- Continued study intervention according to study arms' assignment, if indicated
- Better Health-Related Quality of Life (HRQOL) assessment based on questionnaires, addressing physical, psychological, emotional, and social issues.

9.4.6 **Visit F6 (48-weeks follow-up visit)**

- Physical Exam
- Vital Signs
- Laboratory tests including serum levels of CA125
- Chest, abdomen and pelvis CT scan with or w/o contrast or other radiological imaging indicated.
- Continued study intervention according to study arms' assignment, if indicated
- Better Health-Related Quality of Life (HRQOL) assessment based on questionnaires, addressing physical, psychological, emotional, and social issues.

9.4.7 **Visit F7 (60-weeks follow-up visit)**

- Physical Exam
- Vital Signs
- Laboratory tests including serum levels of CA125
- Chest, abdomen and pelvis CT scan with or w/o contrast or other radiological imaging indicated.
- Continued study intervention according to study arms' assignment, if indicated
- Better Health-Related Quality of Life (HRQOL) assessment based on questionnaires, addressing physical, psychological, emotional, and social issues.

9.4.8 **Visit F8 (72-weeks follow-up visit)**

- Physical Exam
- Vital Signs
- Laboratory tests including serum levels of CA125
- Chest, abdomen and pelvis CT scan with or w/o contrast or other radiological imaging indicated.
- Continued study intervention according to study arms' assignment, if indicated
- Better Health-Related Quality of Life (HRQOL) assessment based on questionnaires, addressing physical, psychological, emotional, and social issues.

9.4.9 **Visit F9 (84-weeks follow-up visit)**

- Physical Exam
- Vital Signs
- Laboratory tests including serum levels of CA125
- Chest, abdomen and pelvis CT scan with or w/o contrast or other radiological imaging indicated.
- Continued study intervention according to study arms' assignment, if indicated
- Better Health-Related Quality of Life (HRQOL) assessment based on questionnaires, addressing physical, psychological, emotional, and social issues.

9.4.10 **Visit F10 (96-weeks - 18 months-follow-up visit – End of study Visit)**

- Physical Exam
- Vital Signs
- Laboratory tests including serum levels of CA125
- Chest, abdomen and pelvis CT scan with or w/o contrast or other radiological imaging indicated.
- Continued study intervention according to study arms' assignment, if indicated
- Better Health-Related Quality of Life (HRQOL) assessment based on questionnaires, addressing physical, psychological, emotional, and social issues.

9.4.11 **Follow-up phone calls: (Follow-up phone calls)**

- 6 and 12 months following Visit F10 there will be a phone call to assess survival status.

9.5 Unscheduled Visits

Unscheduled visits will be handled as part of standard-of-care visits. Participant will be assesses by:

- Physical Exam
- Vital Signs
- Laboratory tests including serum levels of CA125
- Chest, abdomen and pelvis CT scan with or w/o contrast or other radiological imaging indicated.
- Better Health-Related Quality of Life (HRQOL) assessment based on questionnaires, addressing physical, psychological, emotional, and social issues.

9.6 Concomitant Medication

N/A

9.7 Rescue Medication Administration

N/A

9.8 Subject Completion/Withdrawal

Subjects may withdraw from the study at any time without prejudice to their care. They may also be discontinued from the study at the discretion of the Investigator for lack of adherence to study treatment or visit schedules, and AEs. The Investigator or the Sponsor may also withdraw subjects who violate the study plan, or to protect the subject for reasons of safety or for administrative reasons. It will be documented whether or not each subject completes the clinical study. If the Investigator becomes aware of any serious, related adverse events after the subject completes or withdraws from the study, they will be recorded in the source documents and on the CRF.

9.8.1 Early Termination Study Visit

Subjects who withdraw from the study will have all procedures enumerated for Follow-up Visit 12 (end of study visit) as the early termination visit.

10 STUDY EVALUATIONS AND MEASUREMENTS

All laboratory exams and imaging monitoring procedure, measurements and therapeutic interventions listed in Section 4 will be done following standard-of-care procedure.

Better Health-Related Quality of Life (HRQOL) will be assessed using standardized self-reported, validated questionnaires, addressing physical, psychological, emotional, and social issues.

10.1 Screening and Monitoring Evaluations and Measurements

10.1.1 Medical Record Review

Include a listing of the variables that will be abstracted from the medical chart (paper or electronic).

- Age
- Gender
- Weight
- Surgical report
- Pathology report
- Steroid and other medication doses over the course of treatment
- ChemoID drug response assay results
- CA125 serum levels (baseline and over the course of treatment)
- Chemotherapy regimens including doses

- All chest, abdomen and pelvis imaging including but not limited to DICOM images of CT scans PET/CT scan, MRI, chest X-Ray, and abdomen ultrasound, as well as reports

- Clinical assessment of disease at baseline and during the course of therapy from GYN-oncologic progress notes

- Health-Related Quality of Life (HRQOL) questionnaires addressing physical, psychological, emotional, and social issues.

10.1.2 **Physical Examination**

Medical history, height and weight, physical examination by physical exam, demographic characteristics (age, gender, race) will be performed and collected.

10.1.3 **Vital Signs**

Body temperature, heart rate, and respiration rate will be recorded.

Blood pressure will be measured with an automated device or with an aneroid sphygmomanometer on the right arm with patient sitting.

10.1.4 **Laboratory Evaluations**

Blood sampling will be performed for the following laboratory evaluations:

10.1.4.1 ***Hematology***

Hematology testing will be performed at the Institutional laboratory of the Principal Investigator.

Table 10.1.4.1.1: Clinical Laboratory Tests

Category	Tests
Hematology	RBC, hemoglobin, hematocrit, platelet count, ANC or WBC with differential, serum glucose levels
Liver function tests	Complete metabolic panel
Renal function tests	Complete metabolic panel
Tumor markers	CA-125

10.1.4.2 ***Pregnancy Testing***

A urine pregnancy test will be performed for female subjects ≥ 18 years of age who are physically capable of becoming pregnant.

10.1.5 **Other Evaluations, Measures**

Other evaluations will concern review of chest/abdomen/pelvis Imaging by CT scan preferentially with contrast, and/or PET/CT scan, MRI, chest X-Ray, and abdomen ultrasound, pathology report, ChemoID drug response assay results, Better Health-Related Quality of Life (HRQOL) assessment based on questionnaires, addressing physical, psychological, emotional, and social issues.

10.2 Efficacy Evaluations

Under standard-of-care, response to therapy will be evaluated according to the RECIST version 1.1 criteria (41). Furthermore, an assessment of ECOG performance status will be performed. The ECOG performance status is a scale used to assess how a patient's disease is progressing, assess how the disease affects the daily living abilities of the patient, and determine appropriate treatment and prognosis.

10.3 Safety Evaluation

Subject safety will be monitored by adverse events, vital signs, physical examinations, and clinical laboratory data. However, the ChemoID assay is a low risk assay for the patients because the drugs prescribed to the participants are part of the standard-of-care for the treated disease. Any side effects from administering chemotherapies are not associated with the assay per se. Any morbidity associated with tissue sampling is also part of standard-of-care and its expected risks are not associated with the assay.

11 STATISTICAL CONSIDERATIONS

11.1 Statistical Methods

11.1.1 Baseline Data

Initial analyses will involve data cleaning, variable development, and exploratory data analyses. We will use standard summaries to describe baseline characteristic distributions in terms of centrality, spread, shape, and possible outliers by treatment group and stratification variables.

11.1.2 Efficacy Analysis

The primary analysis will be based on an intention to treat approach and will include all subjects randomized at baseline. The primary efficacy outcome is objective response rate (ORR). This outcome will be compared between patients randomized to receive the ChemoID informed regimen versus the control arm treatments of Physician choice chemotherapy (see study-schematic above). ORR comparisons will be examined using logistic regression models with main effect adjustments for number of prior platinum treatments, and BRCA status. Models examining adjustments for sex, race, age, and tumor stage will be constructed, as well as models examining moderating effects of these variables (subpopulation investigations).

Secondary analyses will include Cox Proportional Hazard Models for Progression Free Survival (PFS) outcomes. Generalized Linear Models (GLMs) will be used for analyses on objective tumor response (RECIST v1.1 Criteria), CA125 and ECOG/KPS/GOG Performance Status, Duration of Response (DOR), and QOL outcomes. Generalized Linear Mixed Models (GLMMs) will be used for analyses of changes in repeated outcome measures to incorporate within-person associations and examine distributions of participant-specific outcomes. Huber-White robust standard errors will be used, and multiple variance structures will be investigated to examine sensitivity of primary analyses to the choice of association model.

11.1.3 **Safety Analysis**

All evaluable subjects entered into the study at Baseline will be included in the safety analysis. The frequencies of AEs/SAEs by type, body system, severity and relationship to study drug will be summarized. AE/SAE Probabilities and confidence intervals will be compared between the treatment groups.

11.2 Sample Size and Power

For our primary analyses comparing objective response rate (ORR), with N=220, a 1:1 ratio between treatment groups, an overall alpha rate of 0.05 and beta rate of 0.2 and interim analysis described below, we will have over 80% power to detect an improvement in ORR from 10% to 25%, giving a detectable odd ratio of 0.33 at the final analysis.

11.3 Interim Analysis

We have a priori planned interim analyses at total sample sizes of 75, 100, and 150. The stopping guidelines for either benefit or futility are based upon Obrien-Fleming spending functions. At enrollment of 75, a p-value of 0.00005 would need to be observed to conclude the trial and declare either a success or no success. Likewise, at sample sizes of 100 and 150, the corresponding stopping p-values would need to be 0.0039 and 0.0184, respectively. At full collection (N=220), due to the interim analyses and to preserve the experiment-wise error rates, analyses would have to achieve p-values less than 0.0412 to be considered statistically significant.

12 STUDY MEDICATION (STUDY DEVICE OR OTHER STUDY INTERVENTION)

12.1 Description

This clinical study utilizes only FDA approved chemotherapies that are currently used for the treatment of recurrent EOC (**see Table 4.1.1**).

13 SAFETY MANAGEMENT

The ChemoID assay is a low risk assay for the patients. Any side effects from administering chemotherapies are not associated with the assay per se. Any morbidity associated with the sampling is part of standard-of-care and its expected risks are not associated with the assay.

13.1 Clinical Adverse Events and Serious Events

Clinical adverse events due to standard-of-care drugs (AEs) will be monitored throughout the study.

This study will utilize the NCI Common Terminology Criteria for Adverse Events

(CTCAE) version 5.0 for CTEP-AERS (CTEP Adverse Event Reporting System) CAERs reporting of adverse events (AEs), located on the CTEP web site,

http://ctep.cancer.gov/protocolDevelopment/electronic_applications/ctc.htm. All appropriate treatment areas should have access to a copy of the CTCAE version 5.0.

13.1.1 Definition of an Adverse Event (AE)

Any untoward medical occurrence associated with the use of a drug in humans, whether or not considered drug related. Therefore, an AE can be any unfavorable and unintended sign (including an abnormal laboratory finding), symptom, or disease temporally associated with the use of a medicinal (investigational) product, whether or not considered related to the medicinal (investigational) product (attribution of unrelated, unlikely, possible, probable, or definite). (International Conference on Harmonisation [ICH], E2A, E6).

For multi-modality trials, adverse event reporting encompasses all aspects of protocol treatment including radiation therapy, surgery, device, and drug.

Due to the risk of intrauterine exposure of a fetus to potentially teratogenic agents, the pregnancy of a study participant must be reported via CTEP-AERS in an expedited manner.

13.2 Adverse Event Reporting

Since the study procedures are not greater than minimal risk, SAEs are not expected. If any unanticipated problems related to the research involving risks to subjects or others happen during the course of this study (including SAEs) they will be reported to the IRB in accordance with ASRI-WPAHS IRB SOP 011: Unanticipated Problems Involving Risks to Subjects. AEs that are not serious but that are notable and could involve risks to subjects will be summarized in narrative or other format and submitted to the IRB at the time of continuing review.

14 STUDY ADMINISTRATION

14.1 Treatment Assignment Methods

Eligible participants (Female participants with poor prognosis recurrent platinum-resistant epithelial ovarian cancer) will be *randomly* assigned to the two study arms according to their main characteristic:

- **Arm 1**: participants will be treated with control treatment (chemotherapy chosen by the Physician from the provided list)
- **Arm 2**: participants will be treated with ChemoID-guided standard-of-care chemotherapy drugs from the provided list.

14.1.1 Randomization

Participants will be randomized using a statistical random number generator embedded in the REDCap EDC. Study investigators will be kept blind to the schedule. A password-protected web-portal will be available for study-personnel to access patient randomization assignments. Backup CSV files will be automatically updated each time an assignment is made and held for records.

14.1.2 Blinding

Regarding **ARM 1** (participants treated with standard-of-care chemotherapy chosen by the Physician from the provided list), the investigators, trial personnel, and participants will be blinded to the ChemoID drug response assay results.

Investigators, trial personnel, and participants will receive from the ChemoID lab the test results **only** for subject assigned to **ARM-2** of the study (participants treated with ChemoID-guided standard-of-care chemotherapy drugs).

14.1.3 Unblinding

N/A

14.2 Data Collection and Management

Primary records (source documents) and case report forms (CRF) will be collected in paper format and subsequently entered to REDCap, a secure cloud–based clinical data management systems used to electronically capture, manage, and report clinical research data. REDCap provides web-based case report forms, real-time data entry validation, audit trails, and the ability to set up a calendar to schedule and track critical clinical study events such as radiological imaging, participant visits, etc. In addition, the software allows identifying and protecting fields that contain Protected Health Information (PHI) data by employing user rights that are set by the study administrator to control who can view, modify, or add data and/or forms. All activities are system logged, and are available in a full audit trail. Confidentiality will be maintained by keeping a master list containing patient health information (PHI) by using a subject identification (ID) number that have only a study ID number. REDCap also offer a data export utility, which enables to export data in an automated manner into formats that are compatible with commonly used statistical analysis packages such as SAS, SPSS, Stata, and R.

14.3 Confidentiality

All data and records generated during this study will be kept confidential in accordance with Institutional policies and HIPAA on subject privacy and that the Investigator and other site personnel will not use such data and records for any purpose other than conducting the study. ChemoID personnel are HIPAA trained and certified. No identifiable data will be used for future study without first obtaining IRB approval. The investigator will obtain a data use agreement between the provider (the PI) of the data and any recipient researchers (including others at the *Investigator Institution*) before sharing a limited dataset (PHI limited to dates and zip codes).

14.4 Regulatory and Ethical Considerations

This study will be conducted in accordance with Good Clinical Practice (GCP) requirements described in the current revision of International Conference on Harmonisation of Technical Requirements of Pharmaceuticals for Human Use (ICH) Guidelines and all applicable regulations, including current United States Code of Federal Regulations (CFR), Title 21, Parts 11, 50, 54, 56, and 312 and Title 45, Part 164. Compliance with these regulations and guidelines also constitutes compliance with the ethical principles described in the current revision of the Declaration of Helsinki. This study will also be carried out in accordance with local legal requirements.

Prior to initiating this study, this protocol will be reviewed and approved by the appropriate local IRB. The composition and conduct of this committee will conform to the United States CFR.

The IRB will also review and approve the site's informed consent form (ICF), other written information provided to the patient that may be used for patient recruitment.

If it is necessary to amend the protocol or the ICF during the study, the Investigator will be responsible for ensuring that the IRB reviews and approves these amended documents. An IRB approval of the amended protocol and/or ICF must be obtained in writing before implementation of the amended procedures and before new patients are consented to participate in the study using the amended version of the ICF.

14.4.1 **Data and Safety Monitoring Plan**

The ChemoID drug response assay is a low risk assay for the patients. Any side effects from administering chemotherapies are not associated with the test per se. Any morbidity associated with the sampling is part of standard-of-care and its expected risks are not associated with the drug response assay. This is a prospective randomized clinical study in which the risk of the participants is low also because all the drugs used are already FDA approved to treat the specific disease. This clinical study is not evaluating the efficacy of the drugs, but the ability of the ChemoID drug response assay to predict standard-of-care drugs that will provide better outcomes to the patients who are treated with ChemoID-guided therapy vs. subjects treated with standard-of-care drugs empirically chosen by the Physician.

Interim analyses will be conducted at times coincident with regularly scheduled meetings of the *Investigator Institution*-appointed Data and Safety Monitoring Board (DSMB) at approximately six-month intervals. The DSMB Chair will be notified each time an SAE occurs. The DSMB will determine if an evaluation of unblinded AE data (including SAEs) when 20 EOC patients have completed 1 month of follow-up (Visit 1) is necessary. Other safety data, such as laboratory data will also be evaluated by the DSMB as appropriate. Monitoring of key safety endpoints will be conducted as described above, and if rates significantly exceed pre-set thresholds, ChemoID Sponsor will be notified and information will be supplied to the DSMB. All SAEs, at least possibly related, will also be sent to the DSMB chair. The Project Officer (or designee) will be responsible for reviewing the SAE materials to determine if the documents are complete. If there are any concerns regarding the type or frequency of the event, the Project Officer will request that the DSMB Executive Secretary notify the DSMB Chair. The DSMB Chair will review the SAE materials, determine if the information is complete, determine if additional DSMB review is required and make recommendations to ChemoID Sponsor concerning continuation of the study. The data coordinating center (DCC) at the *Investigator Institution* will prepare semi-annual summary reports of all

AEs/SAEs for the Project Officer and DSMB Chair. Semi-annual reports will be made available on a secure website and the Project Officer and DSMB Chair will be notified by e-mail when the materials are posted.

14.4.2 **Risk Assessment**

Risks for this study are not greater than minimal because the chemotherapy drugs used for treatments of participants in all the study arms are all FDA approved agents to treat recurrent EOC. Physicians treating participants enrolled in study arms in which the treatment is guided by the ChemoID drug response assay will choose treatments from those indicated as high-cell kill from the test, but always taking into consideration the performance status and general clinical condition of the patients.

The adaptive design of the study regarding Arms 1 and 2 will minimize the risks of harm to participants by providing the principal investigator the ability to suspend the study after 18 months, if higher than standard-of-care risk to therapeutic intervention will be identified.

14.4.3 **Potential Benefits of Trial Participation**

The information obtained from the ChemoID drug response assay will be used to understand if the test can help to achieve better outcomes in recurrent and poor prognosis EOC patients.

Collectively, results of clinical response to empirical therapy or to chemotherapy agents suggested by ChemoID drug response assay will be used to determine the predictive value of the test to clinical response.

The potential direct benefits include better response to chemotherapy and clinical outcome as well as the possible avoidance of potentially ineffective, costly and morbidity producing chemotherapies. Indirect benefits include reducing the chance of ineffective chemotherapy, unnecessary hospitalizations, and lowering the health-care cost.

14.4.4 **Risk-Benefit Assessment**

Selection of effective chemotherapy is extremely important not only when therapy is first initiated but for recurrent disease as well. In fact, administration of ineffective anticancer therapy is associated with unnecessary toxicity and the development of more aggressive cancer cell clones that are resistant to subsequent therapies. The ability to initially choose the most effective chemotherapy may help to avoid the physical, emotional, and financial burden to patients of ineffective therapy, thereby improving their quality of life. Each time patients are treated, they have a chance of relapse, and their cancer will likely become more resistant to therapy. Presently used anticancer drugs have a high rate of failure, and cell culture chemotherapy testing has been used to identify which drugs are more likely to be effective against a particular tumor type. Measuring the response of the tumor cells to drug exposure is valuable in any situation in which there is a choice between two or more treatments. The ChemoID drug response assay is the first and only drug response assay available in the clinics that examine CSCs response from solid tumors.

Results from previous ChemoID clinical studies indicate that the CSC drug response assay may be a very useful tool for optimizing treatment selection when first-line or second-line therapy fails and

when there are multiple clinically acceptable and equivalent treatments available. Furthermore, the ChemoID studies suggest that individualized functional drug response assays provide more treatment options with improved outcomes for many more patients than are currently achieved by empiric population-based treatment. These compelling data suggest also that it is reasonable to prospectively utilize functional testing with the ChemoID drug response assay to assist clinicians in the optimal prioritization of therapy for recurrent EOC patients.

14.5 Recruitment Strategy

Patients coming to gynecological clinical visit consult for symptoms and/or imaging findings related to the presence of a recurring ovarian cancer resistant to platinum therapy will be approached as eligible subjects for the study. Following a discussion on their medical treatment options, candidates will sign an informed consent to be enrolled in the study. Subjects will be in the study if the pathology report of their tumor biopsy will confirm the presence of relapsed epithelial ovarian cancer.

14.6 Informed Consent/Assent and HIPAA Authorization

Before being admitted to the clinical study, all patients will consent in writing to participate. An informed consent form (ICF) will be given to each patient, which will contain all United States federally required elements, all ICH-required elements, and Health Insurance Portability and Accountability Act (HIPAA) authorization information in language that is understandable to the patient.

The process of obtaining the informed consent will be in compliance with all federal regulations, ICH requirements, and local laws.

The Investigator will review the study with each patient. The review will include the nature, scope, procedures, and possible consequences of the patient's participation in the study. The ICF and review will be in a form understandable to the patient. The Investigator or designee and the patient must both sign and date the ICF after review and before the patient can participate in the study. The patient will receive a copy of the signed and dated form, and the original will be retained in the site study files. The Investigator or his/her designee will emphasize to the patient that study participation is entirely voluntary and that consent regarding study participation may be withdrawn at any time without penalty or loss of benefits to which the patient is otherwise entitled.

If the ICF is amended during the study, the Investigator must follow all applicable regulatory requirements pertaining to approval of the amended ICF by the IRB/IEC. The site must use the amended consent form for all new patients and repeat the consent process with the amended ICF for any ongoing patients.

Patients' names will remain confidential and will not be included in the database. Only patient number, patient initials, and birth date will be recorded in the data system. If the patient name appears on any other document collected (e.g., hospital discharge summary), the name will be obliterated before the document is transmitted. All study findings will be stored in paper format databases. The patients will give explicit permission for representatives of the Sponsor, regulatory authorities, and the IRB to inspect their medical records to verify the information collected.

Patients will be informed that all personal information made available for inspection will be handled in the strictest confidence and in accordance with all state, local, and federal data protection/privacy laws, including, without limitation, the HIPAA.

All participants in the will provide written authorization to disclose private health information either as a part of the written ICF or as a separate authorization form. The authorization will contain all required elements specified by 45 CFR 164, and will contain a waiver of patient access to study-related private health information until the conclusion of the clinical study. The authorization will remain valid and in full force and effect until the first to occur of (1) the expiration of 3 years after the study therapy is approved for the indication being studied, or (2) the expiration of 3 years after the research program is discontinued. Individual patient medical information obtained during this study is confidential and its disclosure to third parties (other than those mentioned in this Section 9.7) is strictly prohibited. In addition, medical information obtained during this study may be provided to the patient's personal physician or to other appropriate medical personnel when required in connection with the patient's continued health and welfare.

The Investigator will maintain a personal patient identification list (patient and treatment numbers with the corresponding patient names) to enable records to be identified. Confidentiality will be maintained by keeping a master list containing patient health information (PHI) and subject identification (ID) number separate from paper data forms that have only a study ID number. The master list will be on a separate computer, removable disk drive or in a locked file cabinet and therefore, this form of data is considered “coded”.

15 PUBLICATION OF CLINICAL DATA

Patient data collected will be examined and analyzed independently by a group of radiologists and biostatisticians. Complete trial data and end of study statistics will be provided to the principal investigators of the multicenter clinical study and the publication write up will be done in collaboration between the investigators.

16 REFERENCES

1. Hanker LC, Loibl S, Burchardi N, Pfisterer J, Meier W, Pujade-Lauraine E, Ray-Coquard I, Sehouli J, Harter P, du Bois A, Ago, group Gs. The impact of second to sixth line therapy on survival of relapsed ovarian cancer after primary taxane/platinum-based therapy. Ann Oncol. 2012;23(10):2605-12.

2. Foley OW, Rauh-Hain JA, del Carmen MG. Recurrent epithelial ovarian cancer: an update on treatment. Oncology (Williston Park). 2013;27(4):288-94, 98.

3. Pujade-Lauraine E, Combe P. Recurrent ovarian cancer. Ann Oncol. 2016;27 Suppl 1:i63-i5.

4. McGee J, Bookman M, Harter P, Marth C, McNeish I, Moore KN, Poveda A, Hilpert F, Hasegawa K, Bacon M, Gatsonis C, Brand A, Kridelka F, Berek J, Ottevanger N, Levy T, Silverberg S, Kim BG, Hirte H, Okamoto A, Stuart G, Ochiai K, participants of the 5th Ovarian Cancer Consensus C. Fifth Ovarian Cancer Consensus Conference: individualized therapy and patient factors. Ann Oncol. 2017;28(4):702-10.

5. Ahmed N, Abubaker K, Findlay JK. Ovarian cancer stem cells: Molecular concepts and relevance as therapeutic targets. Mol Aspects Med. 2014;39:110-25.

6. Aimola P, Desiderio V, Graziano A, Claudio PP. Stem cells in cancer therapy: From their role in pathogenesis to their use as therapeutic agents. Drug News Perspect. 2010;23(3):175-83.

7. Ayub TH, Keyver-Paik MD, Debald M, Rostamzadeh B, Thiesler T, Schroder L, Barchet W, Abramian A, Kaiser C, Kristiansen G, Kuhn W, Kubler K. Accumulation of ALDH1-positive cells after neoadjuvant chemotherapy predicts treatment resistance and prognosticates poor outcome in ovarian cancer. Oncotarget. 2015;6(18):16437-48. PMCID: PMC4599280.

8. Burgos-Ojeda D, Rueda BR, Buckanovich RJ. Ovarian cancer stem cell markers: prognostic and therapeutic implications. Cancer Lett. 2012;322(1):1-7. PMCID: PMC4431611.

9. Chen X, Zhang J, Zhang Z, Li H, Cheng W, Liu J. Cancer stem cells, epithelial-mesenchymal transition, and drug resistance in high-grade ovarian serous carcinoma. Hum Pathol. 2013;44(11):2373-84. PMCID: PMC3797876.

10. Cioffi M, D'Alterio C, Camerlingo R, Tirino V, Consales C, Riccio A, Ierano C, Cecere SC, Losito NS, Greggi S, Pignata S, Pirozzi G, Scala S. Identification of a distinct population of CD133(+)CXCR4(+) cancer stem cells in ovarian cancer. Sci Rep. 2015;5:10357. PMCID: PMC4650662.

11. Davidson B, Holth A, Hellesylt E, Tan TZ, Huang RY, Trope C, Nesland JM, Thiery JP. The clinical role of epithelial-mesenchymal transition and stem cell markers in advanced-stage ovarian serous carcinoma effusions. Hum Pathol. 2015;46(1):1-8.

12. Dyall S, Gayther SA, Dafou D. Cancer stem cells and epithelial ovarian cancer. J Oncol. 2010;2010:105269. PMCID: PMC3034964.

13. He QZ, Luo XZ, Wang K, Zhou Q, Ao H, Yang Y, Li SX, Li Y, Zhu HT, Duan T. Isolation and characterization of cancer stem cells from high-grade serous ovarian carcinomas. Cell Physiol Biochem. 2014;33(1):173-84.

14. Hu L, McArthur C, Jaffe RB. Ovarian cancer stem-like side-population cells are tumourigenic and chemoresistant. Br J Cancer. 2010;102(8):1276-83. PMCID: PMC2856005.

15. Wiechert A, Saygin C, Thiagarajan PS, Rao VS, Hale JS, Gupta N, Hitomi M, Nagaraj AB, DiFeo A, Lathia JD, Reizes O. Cisplatin induces stemness in ovarian cancer. Oncotarget. 2016;7(21):30511-22. PMCID: PMC5058697.

16. Zeimet AG, Reimer D, Sopper S, Boesch M, Martowicz A, Roessler J, Wiedemair AM, Rumpold H, Untergasser G, Concin N, Hofstetter G, Muller-Holzner E, Fiegl H, Marth C, Wolf D, Pesta M, Hatina J. Ovarian cancer stem cells. Neoplasma. 2012;59(6):747-55.

17. Howard CM, Valluri J, Alberico A, Julien T, Mazagri R, Marsh R, Alastair H, Cortese A, Griswold M, Wang W, Denning K, Brown L, Claudio PP. Analysis of Chemopredictive Assay for Targeting Cancer Stem Cells in Glioblastoma Patients. Transl Oncol. 2017;10(2):241-54. PMCID: PMC5310181.

18. Howard CM, Alberico A, Valluri J, Griswold M, Claudio PP. Analysis of chemo-predictive assay for targeting cancer stem cells in 41 glioblastoma patients. J Clin Oncol2017. p. (suppl; abstr e13544).

19. Cortese A, Pantaleo G, Amato M, Lawrence L, Mayes V, Brown L, Sarno MR, Valluri J, Claudio PP. A new complementary procedure for patients affected by head and neck cancer: Chemo-predictive assay. Int J Surg Case Rep. 2016;26:42-6. PMCID: PMC4963245.

20. Claudio PP, Mathis SE, Nande R, Lawrence L, Alberico A, Julien TJ, Mazagri RS, Marsh R, Muizelaar P, Denning KL, Valluri J. ChemoID Assay for Glioblastoma. ASCO Annual Meeting; Chicago2015.

21. Mathis SE, Alberico A, Nande R, Neto W, Lawrence L, McCallister DR, Denvir J, Kimmey GA, Mogul M, Oakley G, 3rd, Denning KL, Dougherty T, Valluri JV, Claudio PP. Chemo-predictive assay for targeting cancer stem-like cells in patients affected by brain tumors. PLoS One. 2014;9(8):e105710. PMCID: PMC4140819.

22. Claudio PP, Mathis SE, Nande R, Alberico A, Neto W, Lawrence L, Denvir J, Kimmey G, Chowdhari A, Tirona MRBT, Mogul MJ, Julien TJ, Mazagri RS, Oakley G, Denning KL, Dougherty T, Brown L, Valluri J. Novel chemosensitivity assay for targeting cancer stem-like cells in brain tumors. ASCO Annual Meeting; Chicago2014.

23. Claudio PP, Valluri J, E Mathis SE, Alberico A, Alberico T, Denvir J, Kimmey GA, Mogul MJ, Sehgal R, Chowdhary AA, Mozayen M, Tirona MRBT, Matt LB, Oakley G, Denning KL, Dougherty T. Chemopredictive assay for patients with primary brain tumors. ASCO Annual Meeting; Chicago2013.

24. DeEulis T, Howard CM, Valluri J, Claudio PP. Efficacy of ChemoID guided drug selection for palliative chemotherapy in advanced recurrent high-grade ovarian adenocarcinoma: Case Study. Transl Med Rep. 2017;1(1):70-5.

25. Claudio PP, editor. Chemosensitivity assay for targeting cancer stem-like cells in malignant brain tumors. Cancer Stem Cell Conference; 2014 August; Cleveland, OH.

26. Claudio PP, editor. Prospective Analysis of Chemo-Predictive Assay for Targeting Cancer Stem Cells in Glioblastoma Patients. Cancer Stem Cell Conference 2016 September; Cleveland, OH.

27. Cannistra SA. Cancer of the ovary. N Engl J Med. 2004;351(24):2519-29.

28. du Bois A, Luck HJ, Meier W, Adams HP, Mobus V, Costa S, Bauknecht T, Richter B, Warm M, Schroder W, Olbricht S, Nitz U, Jackisch C, Emons G, Wagner U, Kuhn W, Pfisterer J, Arbeitsgemeinschaft Gynakologische Onkologie Ovarian Cancer Study G. A randomized clinical trial of cisplatin/paclitaxel versus carboplatin/paclitaxel as first-line treatment of ovarian cancer. J Natl Cancer Inst. 2003;95(17):1320-9.

29. Ozols RF, Bundy BN, Greer BE, Fowler JM, Clarke-Pearson D, Burger RA, Mannel RS, DeGeest K, Hartenbach EM, Baergen R, Gynecologic Oncology G. Phase III trial of carboplatin and paclitaxel compared with cisplatin and paclitaxel in patients with optimally resected stage III ovarian cancer: a Gynecologic Oncology Group study. J Clin Oncol. 2003;21(17):3194-200.

30. Ledermann JA. Primary chemotherapy: the future for the management of advanced ovarian cancer? Int J Gynecol Cancer. 2010;20(11 Suppl 2):S17-9.

31. Markman M. The genetics, screening, and treatment of epithelial ovarian cancer: an update. Cleve Clin J Med. 2000;67(4):294-8.

32. Markman M, Bookman MA. Second-line treatment of ovarian cancer. Oncologist. 2000;5(1):26-35.

33. Matsuo K, Eno ML, Im DD, Rosenshein NB. Chemotherapy time interval and development of platinum and taxane resistance in ovarian, fallopian, and peritoneal carcinomas. Arch Gynecol Obstet. 2010;281(2):325-8.

34. Moore KN, Secord AA, Geller MA, Miller DS, Cloven N, Fleming GF, Wahner Hendrickson AE, Azodi M, DiSilvestro P, Oza AM, Cristea M, Berek JS, Chan JK, Rimel BJ, Matei DE, Li Y, Sun K, Luptakova K, Matulonis UA, Monk BJ. Niraparib monotherapy for late-line treatment of ovarian cancer (QUADRA): a multicentre, open-label, single-arm, phase 2 trial. Lancet Oncol. 2019.

35. Stockler MR, Hilpert F, Friedlander M, King MT, Wenzel L, Lee CK, Joly F, de Gregorio N, Arranz JA, Mirza MR, Sorio R, Freudensprung U, Sneller V, Hales G, Pujade-Lauraine E. Patient-reported outcome results from the open-label phase III AURELIA trial evaluating bevacizumab-containing therapy for platinum-resistant ovarian cancer. J Clin Oncol. 2014;32(13):1309-16. PMCID: PMC4876313.

36. Pujade-Lauraine E, Hilpert F, Weber B, Reuss A, Poveda A, Kristensen G, Sorio R, Vergote I, Witteveen P, Bamias A, Pereira D, Wimberger P, Oaknin A, Mirza MR, Follana P, Bollag D, Ray-Coquard I. Bevacizumab combined with chemotherapy for platinum-resistant recurrent ovarian cancer: The AURELIA open-label randomized phase III trial. J Clin Oncol. 2014;32(13):1302-8.

37. Aghajanian C, Blank SV, Goff BA, Judson PL, Teneriello MG, Husain A, Sovak MA, Yi J, Nycum LR. OCEANS: a randomized, double-blind, placebo-controlled phase III trial of chemotherapy with or without bevacizumab in patients with platinum-sensitive recurrent epithelial ovarian, primary peritoneal, or fallopian tube cancer. J Clin Oncol. 2012;30(17):2039-45. PMCID: PMC3646321.

38. Aghajanian C, Goff B, Nycum LR, Wang YV, Husain A, Blank SV. Final overall survival and safety analysis of OCEANS, a phase 3 trial of chemotherapy with or without bevacizumab in patients with platinum-sensitive recurrent ovarian cancer. Gynecol Oncol. 2015;139(1):10-6. PMCID: PMC4993045.

39. Coleman RL, Brady MF, Herzog TJ, Sabbatini P, Armstrong DK, Walker JL, Kim BG, Fujiwara K, Tewari KS, O'Malley DM, Davidson SA, Rubin SC, DiSilvestro P, Basen-Engquist K, Huang H, Chan JK, Spirtos NM, Ashfaq R, Mannel RS. Bevacizumab and paclitaxel-carboplatin chemotherapy and secondary cytoreduction in recurrent, platinum-sensitive ovarian cancer (NRG Oncology/Gynecologic Oncology Group study GOG-0213): a multicentre, open-label, randomised, phase 3 trial. Lancet Oncol. 2017;18(6):779-91.

40. Rutherford T, Orr J, Jr., Grendys E, Jr., Edwards R, Krivak TC, Holloway R, Moore RG, Puls L, Tillmanns T, Schink JC, Brower SL, Tian C, Herzog TJ. A prospective study evaluating the clinical relevance of a chemoresponse assay for treatment of patients with persistent or recurrent ovarian cancer. Gynecol Oncol. 2013;131(2):362-7.

41. Rustin GJ, Vergote I, Eisenhauer E, Pujade-Lauraine E, Quinn M, Thigpen T, du Bois A, Kristensen G, Jakobsen A, Sagae S, Greven K, Parmar M, Friedlander M, Cervantes A, Vermorken J, Gynecological Cancer I. Definitions for response and progression in ovarian cancer clinical trials incorporating RECIST 1.1 and CA 125 agreed by the Gynecological Cancer Intergroup (GCIG). Int J Gynecol Cancer. 2011;21(2):419-23.

APPENDIX

1. Case Report Form (CRF) for recording data
2. Informed Consent (IC) and HIPAA form
3. Better Health-Related Quality of Life (HRQOL) Questionnaire
4. Adverse Event (AE) form
5. Concomitant Medications (CM) form
